# Strong Bases Design: Key Techniques and Stability Issues

**DOI:** 10.3390/ijms25168716

**Published:** 2024-08-09

**Authors:** Andrey V. Kulsha, Oleg A. Ivashkevich, Dmitry A. Lyakhov, Dominik Michels

**Affiliations:** 1 Chemical Department, Belarusian State University, 14 Leningradskaya Str., 220006 Minsk, Belarus; kulshaav@bsu.by; 2 Research Institute for Physical Chemical Problems, Belarusian State University, 14 Leningradskaya Str., 220006 Minsk, Belarus; 3 Computer, Electrical and Mathematical Science and Engineering Division, 4700 King Abdullah University of Science and Technology, Thuwal 23955-6900, Saudi Arabia; dmitry.lyakhov@kaust.edu.sa (D.A.L.); dominik.michels@kaust.edu.sa (D.M.)

**Keywords:** ab initio calculations, basicity, DLPNO-CCSD(T), stability, superbases

## Abstract

Theoretical design of molecular superbases has been attracting researchers for more than twenty years. General approaches were developed to make the bases potentially stronger, but less attention was paid to the stability of the predicted structures. Hence, only a small fraction of the theoretical research has led to positive experimental results. Possible stability issues of extremely strong bases are extensively studied in this work using quantum chemical calculations on a high level of theory. Several step-by-step design examples are discussed in detail, and general recommendations are given to avoid the most common stability problems. New potentially stable structures are theoretically studied to demonstrate the future prospects of molecular superbases design.

## 1. Introduction

**Figure 1:**
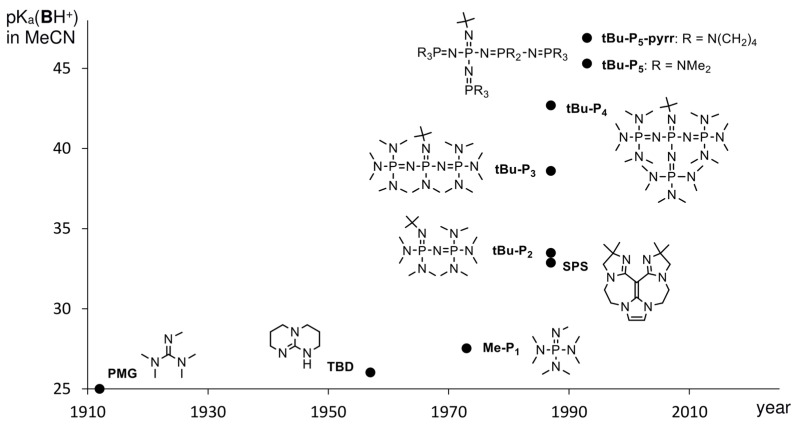
Discovery timeline of the strongest molecular bases with nitrogen basicity centers.

The history of strong molecular organic bases goes back more than a century. After the synthesis of pentamethylguanidine (PMG) by Schenck [1] in 1912, progressively stronger molecular bases with nitrogen basicity centers such as 1,5,7-triazabicyclo[4.4.0]dec-5-ene [2] (TBD) or MeN=P(NMe_2_)_3_ [3] (Me-P_1_) have been discovered. However, that strength growth has been uneven: the jump of about 20 orders of magnitude was performed as soon as Reinhard Schwesinger applied the “homologization” concept to amidines [4,5] and phosphazenes [6,7] (Figure 1).

**Scheme 1:**
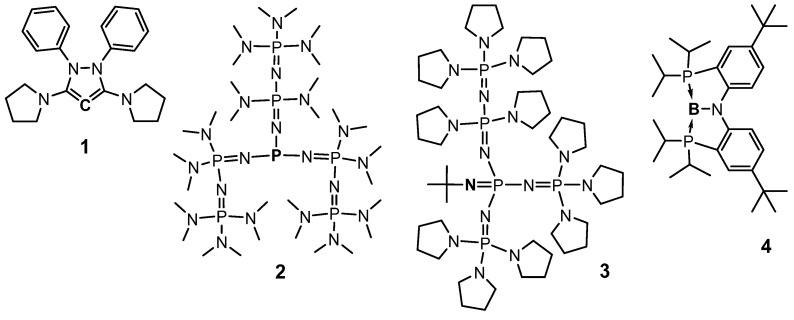
The most basic known carbene **1** [15], phosphine **2** [17], azene **3** [7], and borylene **4** [20]. Protonation sites are shown in bold.

Since then, Schwesinger’s proton sponge (SPS) and polyphosphazenes from P_2_ to P_5_ [8] have come to be used as benchmarks to assess the strength of newly synthesized molecular bases. Numerous applications of strong molecular bases in organic synthesis [9,10,11] led to increased interest in various kinds of molecular bases [12,13], including carbenes [14,15], phosphines [16,17,18,19], and even borylenes [20]. However, none of them exceeded the strength of tBu-P_5_-pyrr **3** significantly (Scheme 1). If metal-free ionic bases are taken into account, then Schwesinger’s phosphazenium fluorides [21,22] expand the scale by several orders of magnitude, with [P(N=P(NMe_2_)_3_)_4_]^+^F^−^ as the absolute record holder.

Consequently, a challenge arose to design new molecular bases. To reach the high basicity of an arbitrary molecule **B** being designed, one should either stabilize its protonated form **B**H^+^ or destabilize its neutral form **B**. Key techniques to do this are summarized as a general five-step design algorithm.

*Step 1*:  choosing the basicity center. That should be a negatively charged atom with a lone electron pair available for protonation. The trivalent nitrogen atom has been a common choice for more than a century; however, the generally weaker acidity of C–H bond compared to N–H bond makes carbene bases generally stronger. Nevertheless, a lot depends on further steps, making it possible to design strong bases with other types of basicity centers [16,17,18,19,20,21,22].
*Step 2*:  steric loading of the basicity center by groups with lone electron pairs, which destabilize **B** to a greater extent than **B**H^+^. That is implemented, for example, in various classes of “proton sponges” [23,24,25,26,27], Verkade bases [28,29], cyclic aminopyridines [30,31], rotaxane or catenane superbases [32,33,34], and adamanzanes [34,35,36].
*Step 3*:  “homologization principle”: the parent electron-rich unit such as amidine [4,5], guanidine [37], phosphazene [6,7,18], or cyclopropenimine [38] moiety is repeated several times to build a large conjugated structure that delocalizes the positive charge in **B**H^+^.
*Step 4*:  adding donor groups such as OMe, NMe_2_, N(CH_2_)_4_, N=C(NMe_2_)_2_, N=P(NMe_2_)_3_ to the conjugated framework [12,13,39] to increase the electron density on the protonation site in **B**.
*Step 5*:  adjusting the structure to additionally stabilize **B**H^+^ with hydrogen bonds or other intramolecular interactions [40,41].

Although the guide generally works for various types of bases, not all the steps may be necessary at the same time. For example, protonated carbene bases are usually bad hydrogen bond donors, which makes *Step 5* almost useless for them. Another point is that every step has its own limitations. Thus, an excess of donor group introduction at *Step 4* may lead to conjugation breaking, while too large steric hindrance provided at *Step 3* may turn the base kinetically inhibited.

The availability of quantum chemical research methods has stimulated theoretical studies of various potentially superbasic structures in the last 25 years [42,43,44,45,46,47,48,49,50,51,52,53,54,55,56,57,58,59,60,61,62,63,64,65,66,67,68,69,70,71,72,73,74,75,76,77,78,79,80,81,82,83,84]. Although many of these predicted structures were theoretically expected to surpass the current basicity champions, none of them actually did. We have previously shown that these inconsistencies may be quite predictable by the same quantum chemistry methods as those used for the design [85]. The goals of this article are to develop general rules to avoid the most common problems while designing new superbases and to provide specific examples of such a design.

The strength of a given base **B** may be measured and computed in the gas phase [86,87,88,89,90] as well as in the solution [89,90,91,92,93,94,95]. In the latter case, p*K*_a_(**B**H^+^) values for solvents like THF, DMSO, or MeCN are commonly used. These values become formal for the strongest bases due to solvent degradation [8,22], but that is not the case for hexamethylphosphoramide (HMPA) because of its exceptional stability in highly basic media [85,96]. Indeed, HMPA withstands the presence of deprotonated toluene [97] and deprotonated THF [98], not to mention its ability to solvate electrons [99]. Although HMPA had been suspected of being carcinogenic [100], it was doubted later [101]. Thus, computed p*K*_a_(**B**H^+^) values in HMPA are used in this work to compare the basicity of the considered structures. According to our previous studies [85,96,102], good linear correlation with experimental p*K*_a_ values in other solvents suggests that computed p*K*_a_ values should be quite accurate with respect to each other, although some systematic bias may still appear. To give an idea of the basicity scale in HMPA, a few calculated p*K*_a_(**B**H^+^) anchor points are given in Table 1.

## 2. Results and Discussion

### 2.1. Flawed Design Examples

To illustrate the possible problems arising upon molecular superbase design, a few sample structures are considered.

**Scheme 2:**
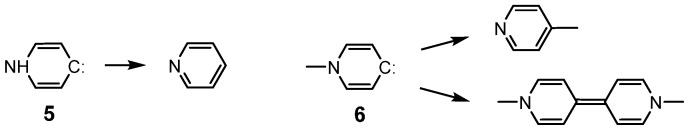
Sample superbasic structures and their degradation ways.

Structure **5** (Scheme 2) might have been quite a strong carbene base with computed p*K*_a_(**B**H^+^) = 38.84. However, the obvious problem is its self-deprotonation leading to pyridine. The reason behind it is that the acidity of the NH group in **5** is too high to make it a stable, strong base. Surprisingly, a lot of structures proposed in the literature suffer from the same problem, and that can be shown with quantum chemical computations as it is done by us for the structure **5**. For example, an unstable tautomer of 4-aminobenzamidine is proposed as a superbase in ref. [44]; certain “croissant” structures from ref. [58] contain too acidic imidazole moieties; proton sponges from ref. [59] could tautomerize due to the acidity of NH_2_ groups; allenes from ref. [66] contain CH-acidic cyclopentadiene moieties; allenes from ref. [84] irreversibly lose the allene moiety upon protonation. In azaphosphiridines from ref. [71], proton migration from endocyclic nitrogen atom to endocyclic carbon atom could lead to ring opening; similar ring-opening tautomerization is possible for the tetrahedrane scaffolds from ref. [74]. The tautomerization of dendritic allenes from ref. [72] is less obvious but has been addressed by us earlier [85].

On the other hand, some authors explicitly warn about the self-deprotonation problem [45,53], e.g., “peralkylation of potential superbases is strongly recommended in order to prevent intramolecular self-protonation of the most basic sites” [53]. Thus, one could propose structure **6** (Scheme 2) to avoid self-protonation. However, unfortunately, **6** can at least undergo either self-demethylation to 4-methylpyridine or dimerization to the neutral form of methylviologen [103]. Such inter- or intramolecular rearrangements should always be considered as other possible degradation ways for the structure being designed. Unlike self-deprotonation, they are generally less obvious, but the common routes are either dimerization or nucleophilic attack of positively charged Lewis acidic sites by negatively charged basicity centers. Carbenes [63,65], silylenes [60,69], and germylenes [67] are the most susceptible to these degradation routes due to their high reactivity [104,105,106,107]. Two explicit degradation ways for certain structures from ref. [63] have already been considered by us [85], while a similar process has been recently observed in the experiment [108].

**Scheme 3:**
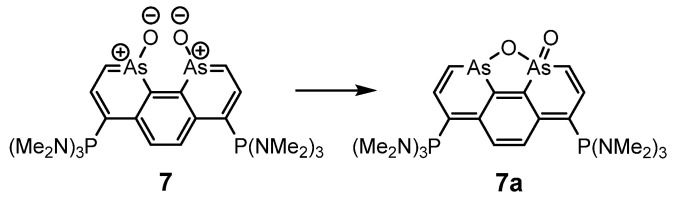
Degradation of the superbase **7** proposed in ref. [68].

Another illustrative example of intramolecular nucleophilic attack is the isomerization of 4,5-diarsaphenanthrene dioxide derivatives from ref. [68]. For structure **7** (Scheme 3), our calculations predict the isomerization to be exergonic by 73 kJ/mol in the HMPA solution and by 128 kJ/mol in the gas phase. Generally, the most basic molecules may become unstable due to the presence of even such weak Lewis acids as cyclopropene groups [64,65,73,75,80]; the corresponding degradation has already been confirmed experimentally [38].

**Scheme 4:**
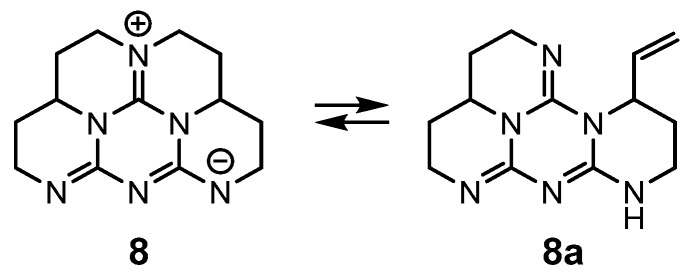
The proposed “homologue” of TBD and its degradation.

Structure **8** (Scheme 4) is a product of applying the “homologization principle” to TBD. At first glance, **8** should be quite stable, and its mesoionic nature suggests high basicity of the outer nitrogen atoms. Indeed, our calculations give p*K*_a_(**B**H^+^) = 21.27 in HMPA for **8**H^+^ and a gas-phase dipole moment of more than 12 Debye for **8**. However, closer analysis reveals its possible tautomerization to **8a** with the corresponding p*K*_taut_ = 0.2. Thus, self-deprotonation may be accompanied by intramolecular rearrangement. The experimental evidence of that was provided by Schwesinger: strongly basic media cause degradation of N(CH_2_)_4_ group to N(CH_2_)_2_CH=CH_2_ group [22], while NC(CH_3_)_3_ group decomposes to NH group and isobutylene [8]. The dimethylamino group seems to be somewhat more stable; however, Lewis acidity of the nearby phosphorus(V) atom may induce CH_2_=NCH_3_ elimination [14].

**Scheme 5:**
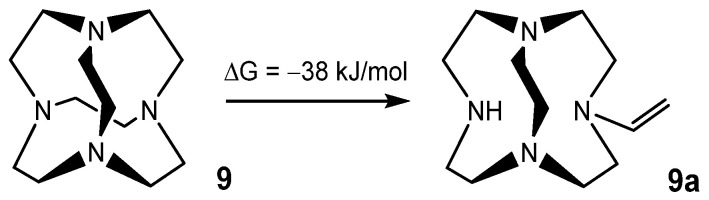
Degradation of hexaethylenetetramine **9** to its opened-cage isomer **9a**.

From the above, one can conclude that under extremely basic conditions, such common designing blocks as P(NMe_2_)_3_ donor groups, ethylene bridges, or even alkyl groups larger than methyl may become unstable. For example, our calculations show that hexaethylenetetramine **9** [35] could have been a strong base with p*K*_a_(**B**H^+^) = 21.05, but it appears to be unstable against self-deprotonation (Scheme 5).

**Scheme 6:**
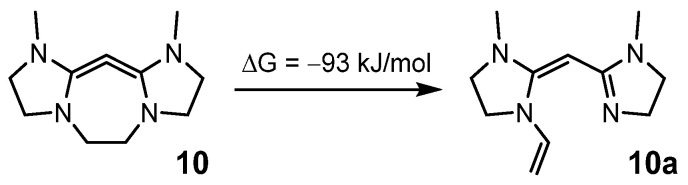
Degradation of the substituted tetraaminoallene **10**.

Another case is substituted tetraaminoallene **10**, the protonated form of which was synthesized by Schwesinger [4] back in 1987. It could have been a record-breaking base with p*K*_a_(**B**H^+^) = 45.19 if not for the degradation to **10a** (Scheme 6).

**Scheme 7:**
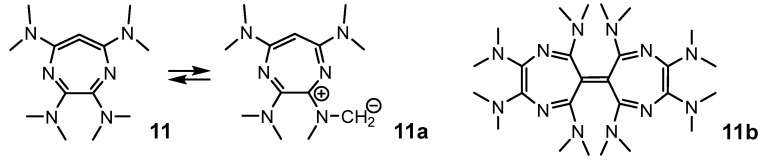
Another proposed superbase **11**, its tautomer **11a**, and dimer **11b**.

One could try to modify **10**, removing the ethylene bridges and making the seven-membered ring conjugated (Scheme 7). According to our calculations, the resulting structure **11** could still be record-breaking with p*K*_a_(**B**H^+^) = 42.71. It passes the self-deprotonation test, although the lowest-lying tautomer **11a** is quite close (p*K*_taut_ = 0.5), but unlike the phosphazene story, the barrier for CH_2_=NCH_3_ elimination from **11a** is reliably high (174 kJ/mol). The protonation site in **11** can be methylated by one of the adjacent NMe_2_ groups in **11**H^+^, but the corresponding barrier for CH_3_^+^ migration is high enough (133 kJ/mol), suggesting years of lifetime in the worst case (both **11** and **11**H^+^ concentrations are high). However, all these stability arguments are ruined by dimerization to **11b**, especially because electron-rich **11b** appears to have too high alkali-metal-like reactivity: the calculated sum of its 1st and 2nd ionization energies is just 9.7 eV. Thus, one should remember that even if dimerization or other structure rearrangement occurs to a minor extent, the high reactivity of the product may be of decisive importance for the original structure stability.

**Scheme 8:**
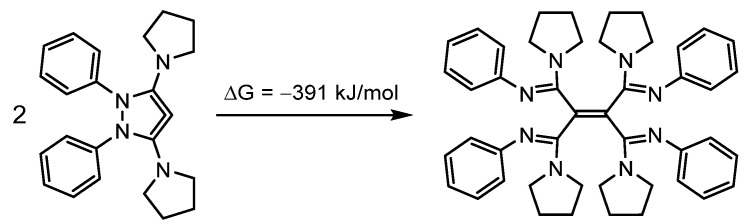
Predicted dimerization of the strongest known carbene superbase **1**.

Looking at the structure of the strongest currently known carbene base **1** (Scheme 1), one could try to improve its basicity [77,79]. However, quite low stability of similar structures [109,110] suggests that dimerization of **1** or its derivatives is possible. Our calculations reveal an interesting situation: while the direct dimerization is not favorable, it becomes quite exergonic if accompanied by N–N bond cleavage, which is essentially the reduction of the hydrazine moiety (Scheme 8). Such a reduction is facilitated by the presence of donor substituents attached to the phenyl rings, that is why more basic derivatives of **1** are still unknown. The dimerization mechanism may involve an open-shell singlet state [111] of **1**, giving an idea why it has not been isolated in pure form yet [15].

**Scheme 9:**
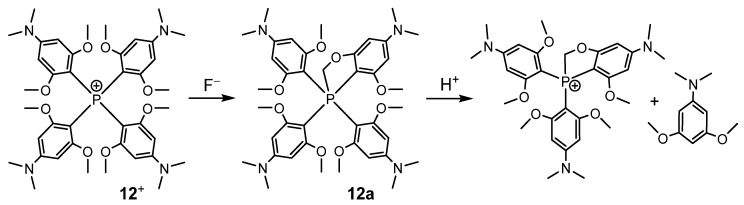
Proposed weakly coordinating cation **12**^+^ and its degradation.

The ultimate basicity of Schwesinger’s phosphazenium fluorides is explained by the large proton affinity of the “naked” fluoride anion and by the ability of the proton to attach two fluoride ions simultaneously (general trends for C, N, O, F basicity centers in molecular and ionic structures have been discussed by us earlier [85]). To improve the basicity, one could build a cation with lower fluoride ion affinity [112] than Schwesinger’s [P(N=P(NMe_2_)_3_)_4_]^+^. According to the recent study [113], donor-substituted tetraphenylphosphonium is worth trying. Since the fluoride anion is small enough to reach the Lewis acidic phosphorus atom in PPh_4_^+^ [114], *ortho*-substituted phenyl groups are preferable to provide the corresponding steric hindrance. Thus, tetrakis[2,6-dimethoxy-4-(dimethylamino)phenyl]phosphonium cation **12**^+^ (Scheme 9) could be suggested to build the potentially record-breaking new superbase **12**^+^F^−^.

Our calculations show that the fluoride ion affinity of **12**^+^ is 39 kJ/mol lower than that of [P(N=P(NMe_2_)_3_)_4_]^+^, but the following problem arises: the methoxy group can undergo deprotonation and attack the nearby phosphorus atom, leading to the neutral molecule **12a**. Indeed, the calculated Gibbs energy change for the reaction

**12**^+^F^−^ + **12**^+^F^−^ = **12a** + **12**^+^HF_2_^−^

is −18 kJ/mol, and the equilibrium might shift further to **12a** because the **12**^+^HF_2_^−^ ion pair seems to dissociate much more than **12**^+^F^−^ in the HMPA solution. Nevertheless, that could still be fine since **12a** appears to be quite a strong molecular base with computed p*K*_a_(**B**H^+^) = 46.39 if the protonation back to **12**^+^ is assumed. However, unfortunately, **12a** protonation proceeds with 3,5-dimethoxy-*N*,*N*-dimethylaniline elimination due to steric overloading of the phosphorus atom (Scheme 9). Considering HCN molecule as a protonation probe, we calculated the protonation barriers to differ by 34 kJ/mol (gas phase) or 9 kJ/mol (HMPA solution) in favor of the degradation route.

### 2.2. Successful Design Examples

Despite a plethora of stability problems arising at a high basicity level, one could still design potentially stable superbases [42,43,46,47,48,49,50,51]. Key techniques to improve the stability of the superbase **B** being designed are summarized as another general five-step guide.

*Step 6.*  Avoid the presence of groups in **B** that are either Brønsted or Lewis acidic, and also groups that tend to rearrange upon deprotonation.
*Step 7.*  Check that both **B** protonation and **B**H^+^ deprotonation are favored at the same sites; identify possible tautomers.
*Step 8.*  Perform a full conformational analysis of all tautomers of both **B** and **B**H^+^ in their ground and low-lying excited states to ensure their relaxation.
*Step 9.*  Check that neither **B** nor **B**H^+^ tends to dimerization or other rearrangements.
*Step 10.*  Look for as many degradation ways of **B** as possible, find the lowest-barrier one, and estimate the corresponding half-life under the conditions being proposed for practical usage, including the possible interaction with the solvent.

**Scheme 10:**
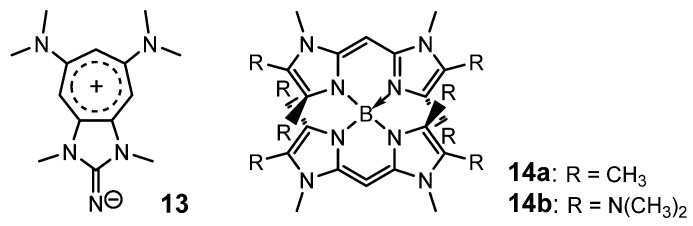
Presumably stable superbases designed by us [85,96].

Our previously designed [85,96] bases **13**, **14a**, and **14b** (Scheme 10) have passed all these tests. While **13** (p*K*_a_(**B**H^+^) = 33.74) is not stronger than tBu-P_4_ and therefore has fewer stability risks, bases **14a** and especially **14b** reach the practical limit of the basicity scale, going quite beyond the deadline of THF degradation. The basicity centers of both **14a** and **14b** are carbon atoms in the middle of CCC moieties, and the degree of their protonation varies from anion to trication depending on pH (Table 2). The closeness of p*K*_a_ values of neutral and monoprotonated forms makes both bases autoionize in HMPA: the autoionization degree is 2% for **14a** and 11% for **14b**.

Methyl group acidity is enough to make both bases tautomerize in the neutral form as well as in the deprotonated anionic form (see [85] for the details on **14a**). Although **14b** is a stronger base than **14a**, the tautomerization of **14b** is less pronounced (p*K*_taut_ = 2.87 and 1.33 for neutral and anionic forms, respectively) and corresponds to deprotonation of the methyl groups attached to endocyclic nitrogen atoms. The acidity of dimethylamino groups is negligible: the corresponding p*K*_taut_ values for neutral **14b** are 9.64 (peripheral NMe_2_ group) and 11.28 (NMe_2_ group closer to the boron atom), and the corresponding tautomers appear to be quite stable against CH_2_=NCH_3_ elimination (the kinetic barriers are 107 and 110 kJ/mol, respectively).

Gas-phase basicity of neutral **14b** was calculated to be 1337 kJ/mol, while the same level of theory gives a lower value of 1332 kJ/mol for the Cs_2_O molecule [85]. Since the latter has been recently proposed as a threshold for hyperbasicity to clarify the meaning of the “hyperbase” term [88], base **14b** appears to be the first molecular hyperbase that is presumably stable and synthetically achievable.

The lowest-barrier degradation path for **14b** was found to be CH_3_^+^ migration from the endocyclic nitrogen atom in the protonated form to the basicity center in the neutral form, like it was found for structure **11**. The calculated kinetic barrier of 127 kJ/mol in HMPA solution suggests a year-scale stability for **14b**, as it was previously shown for **14a** with a similar barrier of 126 kJ/mol [85].

**Scheme 11:**
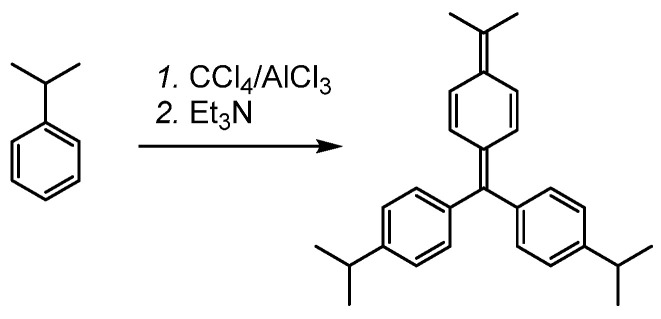
Proposed synthetic route of a new pH indicator for extremely basic media.

Possible synthetic routes to **14a** were addressed earlier [85]; the route to **14b** might be similar except for the NMe_2_ groups introduction, likely requiring the basicity site protection. Protonated forms **14a**H^+^ and **14b**H^+^ are both *D_2d_*-symmetric and rigid, allowing crystallization of the corresponding salts for XRC structure validation. It should also be noted that **14b**H^+^ has little strain and perfect steric hindrance of the boron atom, making it a very promising and thermally stable weak-coordinating cation. According to the calculations, it has 14 kJ/mol lower fluoride ion affinity than [P(N=P(NMe_2_)_3_)_4_]^+^, making **14b**·HF a potentially record-breaking base that could be easier to synthesize than **14b** itself. To track the deprotonation of **14a**H^+^ and **14b**H^+^ in the solution, a new pH indicator synthesizable from cumene and CCl_4_ (Scheme 11) has been proposed by us [102]. It is isoelectronic to crystal violet and possesses sharp color changes from orange-yellow neutral form to green anion (p*K*_a1_ = 45.60) and then to deep blue dianion (p*K*_a2_ = 50.62).

**Scheme 12:**
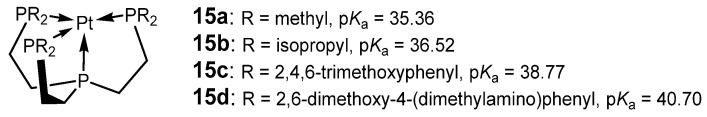
New presumably stable superbases **15a**–**d** and p*K*_a_ values of their protonated forms.

To add some variety to the protonation sites, we also introduce a new series of bases **15a**–**d** (Scheme 12) designed to have a metal atom as the basicity center. They look synthesizable because similar platinum complexes have been known since 1989 [115], and the ligand for **15a** has been known since 1974 [116,117]. The acid–base properties of that kind of metal complexes have already received great attention from both theoretical [118] and experimental [119] points of view, but surprisingly neither of **15a**–**d** has been considered yet. Their exceptional basicity is explained by a specific, like in Verkade bases [29], coordination of the central atom that allows donor substituents to push out the excess electron density to the axial direction. Indeed, the related complex [HPt(dmpe)_2_]^+^, where dmpe = Me_2_PCH_2_CH_2_PMe_2_, has a much lower calculated p*K*_a_ value of 23.21, which makes [Pt(dmpe)_2_] a bit weaker base than SPS (Table 1). The experimentally measured p*K*_a_ values in acetonitrile (32.88 for protonated SPS [120] and 31.1 for [HPt(dmpe)_2_]^+^ [121]) agree with the calculations.

Base **15b** seems to have greater applied significance: it is stronger than tBu-P_4_, has enough steric hindrance of phosphorus atoms, and seems to have no apparent problems with synthesis. Bases **15c** and **15d** are more of theoretical interest: additional steric loading of the basicity center with negatively charged oxygen atoms allows them to reach the level of the strongest known molecular bases (Scheme 1). It is interesting that the related complex Pt(PF_3_)_4_ requires one of the strongest superacids for the protonation [122], demonstrating quite a wide basicity range for the PtP_4_ moiety.

Compared to [Pt(dmpe)_2_], complexes **15a**–**d** are predicted to be less stable. The computed p*K* values in HMPA for the substitution reactions

[PtP(CH_2_CH_2_PR_2_)_3_] + 2dmpe = [Pt(dmpe)_2_] + P(CH_2_CH_2_PR_2_)_3_

are –8.00, –8.95, –14.52, and –15.16 for **15a**–**d**, respectively, showing a clear stability decrease trend. Calculations show that the Pt atom in **15b** is attacked by a PF_3_ molecule with quite a low barrier of 65 kJ/mol. Thus, ligand substitution seems to be the main degradation route for **15a**–**d** in the presence of competing ligands. On the other hand, the chelate effect provides good stability against bulky molecules with low affinity to the Pt atom because four Pt–P bonds must be broken to eliminate the ligand completely. That provides some theoretical evidence of **15a**–**d** stability in the HMPA solution because the HMPA molecule is quite large and has much lower affinity to the Pt atom than phosphine-type ligands. Anyway, even if the vulnerability of **15a**–**d** to small nucleophilic species limits their use as superbases in real applications, it may not diminish the theoretical interest in them as unusual examples of strong base design.

## 3. Methods

The values of p*K*_a_(**B**H^+^) were computed as ΔG/*RT*ln10 − 0.7557, where ΔG is the Gibbs energy change for the proton transfer from the protonated base **B**H^+^ to HMPA molecule in the solution, *R* is the molar gas constant (8.31446 J·mol^–1^·K^–1^), *T* is the temperature (298.15 K), and −0.7557 is the decimal logarithm of the numerical value of HMPA molar volume (dm^3^·mol^–1^) [123]. Gas-phase Gibbs energy of a given species was computed as the sum of gas-phase total energy on the DLPNO-CCSD(T) level of theory [124,125,126,127,128,129,130] and thermal correction on the PBE0 level of theory [131]. Solvation energy was computed on the PBE0 level of theory within the CPCM model [132] using UFF atomic radii [133] scaled by 1.1 and a dielectric constant of 31.6 for HMPA [134]. Geometry optimizations and vibrational frequency calculations were performed on the PBE0 level of theory. Gas-phase basicity calculations were adjusted for the basis set superposition error [135] (BSSE) using geometries of protonated structures. The details on basis sets and the corresponding effective core potentials (ECP) for heavy atoms are presented in Table 3. DLPNO-CCSD(T) calculations were performed within the ORCA 5.0 package [136], while the PBE0 calculations were performed within the Gaussian16 package [137].

The reliability of the chosen levels of theory and basis sets for the computations of basicity in the solution and in the gas phase was shown by us earlier [85,96,102]. Optimized gas phase geometries, DLPNO-CCSD(T) total energies, and Gibbs energies for all structures studied are available in the Supplementary Materials.

## 4. Conclusions

We have briefly reviewed the 25-year history of quantum chemical design of strong molecular bases. Although some of the theoretical predictions, like cyclopropenimines [42] from 1999, hydrogen-bonded guanidines [44] from 2002, or phosphazenyl phosphines [48] from 2006, have found excellent experimental confirmation [17,38,40], the vast majority of theoretically proposed structures remain far from real life. Unfortunately, this situation has led to a gradually growing discredit of quantum chemical methods of design.

Analysis of the existing approaches to strong base design allowed us to formulate a general five-step guide to superbasic molecule construction. More importantly, we have deeply analyzed the unsuccessful cases and identified the main instability reasons of theoretically predicted structures in superbasic media. Providing a few explicit examples of step-by-step design, we have illustrated possible stability problems with sample structures as well as with the examples from the literature. As a result, we have formulated another five-step guide to checking the stability of bases with quantum chemistry methods.

We have introduced new successful cases of strong base design. One of them, **15b**, is predicted to be stronger than tBu-P_4_, while having a metal atom as the protonation site. Another proposed base, **14b**, is predicted to be more basic than the Cs_2_O molecule in the gas phase, which makes **14b** a hyperbase. In the solution, it must surpass the strongest known molecular bases by more than ten orders of magnitude, setting up a practically reachable basicity limit. Meanwhile, the monoprotonated form of **14b** appears to be a robust, highly symmetric weak-coordinating cation.

In general, we can conclude that the lack of experimental confirmation of the theoretical strong base design is not a failure of quantum chemistry but a methodological flaw. Quantum chemistry does provide enough instruments to control the stability of the structures being designed; hence, it does remain a reliable assistant to govern the experiment in the effective direction. Thus, for example, the predicted route of **1** degradation could help to establish the conditions for its isolation in the pure form, while the elusive hexaethylenetetramine could probably be synthesized via derivatives of its predicted tautomer. Careful revising of previously proposed structures that turned out to be unstable might lead to stability improvements. Moreover, the design of stable superbases with new types of protonation sites could be a promising direction of future research.

Anyway, as the growth of available computing power makes quantum chemical research accessible literally to everyone, it becomes very important to follow an efficient methodology for such research. We sincerely hope that our contributions to this methodology will help to build a straight road from theoretical strong bases design to experiment—the criterion of truth.

## Figures and Tables

**Table 1:** Calculated p*K*_a_(**B**H^+^) values in HMPA for a few selected bases [85,102].

Base B	p*K*_a_(BH^+^)
pyridine	1.94
Et_3_N	8.88
TBD	17.01
SPS	25.78
tBu-P_4_	35.65
carbene **1**	39.28
phosphine **2**	40.59
azene **3**	40.82
borylene **4**	40.87

**Table 2:** Calculated p*K*_a_ values for various forms of **14a** and **14b** in HMPA.

Protonation Degree	14a	14b
neutral base	53.02	54.67
monoprotonated	49.04	52.24
diprotonated	10.35	13.01
triprotonated	5.03	9.48

**Table 3:** Basis sets and ECPs utilized for the calculations.

Atoms	Basis Set	ECP	Auxiliary Basis Set for DLPNO-CCSD(T)
H–F	cc-pVTZ [138]	–	cc-pVTZ/C [139]
P	cc-pVTZ [140]	–	cc-pVTZ/C [139]
As	cc-pVTZ-PP [141]	Stuttgart-Köln [142]	AutoAux [143]
Pt	cc-pVTZ-PP [144]	Stuttgart-Köln [144]	AutoAux [143]

## Data Availability

The original contributions presented in the study are included in the Supplementary Material, further inquiries can be directed to the corresponding author.

## Supplementary Materials

The following supporting information can be downloaded at: https://www.mdpi.com/article/10.3390/ijms25168716/s1.
