# Supplementary material for: Strong Bases Design: Key Techniques and Stability Issues
Source: Int J Mol Sci. 2024 Aug 9;25(16):8716. doi: 10.3390/ijms25168716 (PMC11354936; doi:10.3390/ijms25168716)
Supplement: Supplementary file 1 [file ijms-25-08716-s001.zip › ijms-3148431-supplementary.pdf]

Strong bases design: key techniques and stability issues

Andrey V. Kulsha[1], Oleg A. Ivashkevich[2], Dmitry A. Lyakhov[3], Dominik Michels[3]

[1]Belarusian State University, 4 Nezavisimosti avenue, 220030 Minsk, Republic of Belarus

[2]Research Institute for Physical Chemical Problems of the Belarusian State University, 14 Leningradskaya str., 220006 Minsk, Republic of Belarus

[3]Computer, Electrical and Mathematical Science and Engineering Division, 4700 King Abdullah University of Science and Technology, Thuwal 23955-6900, Saudi Arabia

This file contains optimized gas phase geometries, DLPNO-CCSD(T) total energies, and Gibbs energies for the following 76 structures:

1, neutral form
1, protonated form
2, neutral form
2, protonated form
3, neutral form
3, protonated form
4, neutral form
4, protonated form
5, neutral form
5, protonated form
7, neutral form
7a, isomer of **7**
8, neutral form
8a, tautomer of **8**
8, protonated form
9, neutral form
9a, tautomer of **9**
9, protonated form
10, neutral form
10a, isomer of **10**
10, protonated form
11, neutral form
11a, tautomer of **11**
11, protonated form
11a, transition state
11H^+·**11** complex
11H^+·**11** complex, transition state
11b, dimer of **11**
11b^{2+}, dication of **11b**
1, dimer
Schwesinger's $[P(N=P(NMe_2)_3)_4]^+$ cation
$[P(N=P(NMe_2)_3)_4]^+F^-$ ion pair
12$^+$ cation
12$^+F^-$ ion pair
12$^+HF_2^-$ ion pair
12a, neutral form
12a·HCN complex, transition state 1
12a·HCN complex, transition state 2
13, neutral form
13, protonated form
14a, deprotonated form
14a, neutral form
14a, protonated form
14a, diprotonated form
14a, triprotonated form
14b, deprotonated form
14b, neutral form
14b, protonated form
14b, diprotonated form
14b, triprotonated form
14b, deprotonated form, tautomer
14b, neutral form, tautomer 1
14b, neutral form, tautomer 2
14b, neutral form, tautomer 3
14b, neutral form, tautomer 2, transition state
14b, neutral form, tautomer 3, transition state
14bH^+·**14b** complex
14bH^+·**14b** complex, transition state
14bH^+F^- ion pair
15a, neutral form
15a, protonated form
15b, neutral form
15b, protonated form
15c, neutral form
15c, protonated form
15d, neutral form
15d, protonated form
$[Pt(dmpe)_2]$ complex
$[HPt(dmpe)_2]^+$ complex
dmpe free ligand
15a, free ligand
15b, free ligand
15c, free ligand
15d, free ligand
15b·PF_3 complex
15b·PF_3 complex, transition state

1, neutral form
Stoichiometry: $C_{23}H_{26}N_4$
Charge: 0
Multiplicity: 1
Point group: C_2
DLPNO-CCSD(T), Hartree: -1108.9792142
Gibbs in HMPA, Hartree: -1108.5941974
Nuclear coordinates, Å:

C	0.000000	0.000000	2.043070
C	0.028472	1.061179	1.153635
C	-0.028472	-1.061179	1.153635
C	0.458908	3.452806	0.595068
C	0.000000	2.769322	2.869318
C	0.967111	4.511835	1.567014
H	-0.370645	3.839160	-0.007183
H	1.229039	3.100020	-0.095152
C	0.100180	4.285545	2.801011
H	0.847716	2.323490	3.399743
H	-0.903669	2.401643	3.356150
H	2.015848	4.321793	1.809850
H	0.893784	5.520150	1.158523
H	0.522324	4.714658	3.709771
H	-0.889694	4.725049	2.649878
C	-0.458908	-3.452806	0.595068
C	0.000000	-2.769322	2.869318
C	-0.967111	-4.511835	1.567014
H	0.370645	-3.839160	-0.007183
H	-1.229039	-3.100020	-0.095152
C	-0.100180	-4.285545	2.801011
H	-0.847716	-2.323490	3.399743
H	0.903669	-2.401643	3.356150
H	-2.015848	-4.321793	1.809850
H	-0.893784	-5.520150	1.158523
H	-0.522324	-4.714658	3.709771
H	0.889694	-4.725049	2.649878
N	0.019703	2.371572	1.466869
N	-0.019703	-2.371572	1.466869
N	0.048580	0.708893	-0.212960
N	-0.048580	-0.708893	-0.212960
C	-0.866891	1.322703	-1.130921
C	-2.200675	1.506934	-0.784500
C	-0.408524	1.730136	-2.374966
C	-3.069353	2.104024	-1.683036
H	-2.542827	1.180484	0.190790
C	-1.283843	2.319852	-3.276109
H	0.634979	1.581919	-2.623675
C	-2.613222	2.510329	-2.930770
H	-4.107671	2.251983	-1.410545
H	-0.923565	2.636370	-4.247660
H	-3.294801	2.977442	-3.631525
C	0.866891	-1.322703	-1.130921
C	2.200675	-1.506934	-0.784500
C	0.408524	-1.730136	-2.374966
C	3.069353	-2.104024	-1.683036
H	2.542827	-1.180484	0.190790
C	1.283843	-2.319852	-3.276109
H	-0.634979	-1.581919	-2.623675
C	2.613222	-2.510329	-2.930770
H	4.107671	-2.251983	-1.410545
H	0.923565	-2.636370	-4.247660
H	3.294801	-2.977442	-3.631525

1, protonated form
Stoichiometry: $C_{23}H_{27}N_4^+$
Charge: +1
Multiplicity: 1
Point group: C_2
DLPNO-CCSD(T), Hartree: -1109.4729903
Gibbs in HMPA, Hartree: -1109.1088743
Nuclear coordinates, Å:

C	0.000000	0.000000	2.028783
C	-0.156961	1.105734	1.196014
C	0.156961	-1.105734	1.196014
H	0.000000	0.000000	3.102789
C	-0.245397	3.546388	0.701484
C	-0.513844	2.720394	2.970418
C	0.000000	4.668833	1.700612
H	-1.160321	3.714560	0.128321
H	0.575426	3.411156	-0.003720
C	-0.778676	4.216931	2.930474
H	0.417000	2.494975	3.503103
H	-1.321010	2.151226	3.435595
H	1.065171	4.738810	1.932622
H	-0.323678	5.634783	1.316228
H	-0.463784	4.713173	3.846927
H	-1.845952	4.405550	2.795352
C	0.245397	-3.546388	0.701484
C	0.513844	-2.720394	2.970418
C	0.000000	-4.668833	1.700612
H	1.160321	-3.714560	0.128321
H	-0.575426	-3.411156	-0.003720
C	0.778676	-4.216931	2.930474
H	-0.417000	-2.494975	3.503103
H	1.321010	-2.151226	3.435595
H	-1.065171	-4.738810	1.932622
H	0.323678	-5.634783	1.316228
H	0.463784	-4.713173	3.846927
H	1.845952	-4.405550	2.795352
N	-0.382596	2.362944	1.556978
N	0.382596	-2.362944	1.556978
N	-0.060002	0.707450	-0.114681
N	0.060002	-0.707450	-0.114681
C	-0.873589	1.247442	-1.168348
C	-2.257533	1.232689	-1.053823
C	-0.257280	1.753746	-2.301066
C	-3.029424	1.749283	-2.081695
H	-2.723129	0.821023	-0.165947
C	-1.036457	2.263582	-3.329165
H	0.823292	1.747078	-2.369402
C	-2.418953	2.264497	-3.217984
H	-4.108988	1.747948	-1.997693
H	-0.561628	2.662168	-4.216733
H	-3.025115	2.666956	-4.019997
C	0.873589	-1.247442	-1.168348
C	2.257533	-1.232689	-1.053823
C	0.257280	-1.753746	-2.301066
C	3.029424	-1.749283	-2.081695
H	2.723129	-0.821023	-0.165947
C	1.036457	-2.263582	-3.329165
H	-0.823292	-1.747078	-2.369402
C	2.418953	-2.264497	-3.217984
H	4.108988	-1.747948	-1.997693
H	0.561628	-2.662168	-4.216733
H	3.025115	-2.666956	-4.019997

2, neutral form
Stoichiometry: $C_{30}H_{90}N_{21}P_7$
Charge: 0
Multiplicity: 1
Point group: C_1
DLPNO-CCSD(T), Hartree: -4729.7625003
Gibbs in HMPA, Hartree: -4728.6167274
Nuclear coordinates, Å:

C	2.505985	-2.847913	-2.708535
N	1.760490	-1.686030	-2.302073
P	2.432239	-0.332476	-1.527958
N	3.667252	-1.030306	-0.718759
P	4.803317	-0.483849	0.204454
N	5.868501	0.691779	-0.331259
C	6.910297	0.371448	-1.284584
C	0.542007	-1.494181	-3.049388
N	3.198946	0.644683	-2.712227
C	4.107380	-0.013878	-3.621164
N	1.383222	0.572271	-0.827859
P	-0.137477	0.291129	-0.112648
N	-0.194259	1.470697	1.099981
P	-0.284641	3.023930	1.033876
N	1.178921	3.894723	0.931378
C	1.767231	4.146657	-0.367215
C	2.416699	1.664276	-3.375098
N	0.121466	-1.113486	0.802620
P	-0.839851	-2.246988	1.251493
N	0.075765	-3.645889	1.429713
C	1.427069	-3.755665	0.947660
N	-1.488783	-2.103228	2.829645
C	-0.488536	-1.883086	3.858316
N	-2.160731	-2.530025	0.348471
P	-3.216751	-3.435853	-0.309970
N	-4.540882	-3.957929	0.586855
C	-4.296131	-4.884874	1.678214
C	-2.610789	-1.201961	2.993817
N	-0.893998	3.525187	2.531513
C	-0.882775	2.661188	3.683614
N	-1.134126	3.799260	-0.123157
P	-2.615669	3.761924	-0.600494
N	-3.148814	5.353288	-0.713657
C	-2.191505	6.416548	-0.933221
C	-0.932278	4.931533	2.839326
N	5.838040	-1.779556	0.478070
C	6.977512	-1.636927	1.356021
N	4.237104	0.204087	1.615154
C	3.015135	-0.305325	2.217381
C	5.378983	-3.145354	0.356927
C	5.115047	0.834702	2.577512

C	5.418076	2.068588	-0.438276
N	-3.836156	3.071676	0.309778
C	-4.276857	3.722995	1.530225
N	-2.735870	2.946560	-2.049772
C	-1.568954	2.595027	-2.829275
C	-3.886122	1.621304	0.410463
C	2.173343	3.497274	1.903300
C	-3.986523	2.766338	-2.745971
C	-4.474436	5.673356	-1.192923
N	-3.945270	-2.580597	-1.545808
C	-3.322151	-1.407567	-2.122550
N	-2.593506	-4.908433	-0.831368
C	-1.268474	-4.914904	-1.419654
C	-4.948394	-3.190414	-2.387605
C	-3.424836	-5.986494	-1.323685
C	-5.595276	-3.013716	0.905037
C	-0.438314	-4.821618	2.076670
H	1.922829	-3.758200	-2.516622
H	3.434073	-2.910712	-2.146267
H	2.750070	-2.836881	-3.781815
H	-0.025634	-0.661160	-2.631924
H	-0.083150	-2.391547	-2.976862
H	0.728404	-1.307479	-4.118935
H	4.810272	0.714558	-4.041684
H	3.588472	-0.488809	-4.468810
H	4.680480	-0.779697	-3.099602
H	3.077724	2.461671	-3.734485
H	1.702467	2.080663	-2.669763
H	1.866054	1.275338	-4.246294
H	2.502688	0.509993	2.732650
H	3.232355	-1.095152	2.950398
H	2.322589	-0.682526	1.469809
H	7.801489	-2.261282	0.994002
H	6.752369	-1.948939	2.384498
H	7.323002	-0.604846	1.376154
H	4.592042	1.674722	3.045556
H	6.013103	1.217767	2.097078
H	5.412533	0.143923	3.377653
H	7.812719	0.947400	-1.052374
H	6.608819	0.617850	-2.308651
H	7.153320	-0.688044	-1.242646
H	5.131867	-3.578923	1.335207
H	6.163329	-3.762447	-0.095303
H	4.492445	-3.178991	-0.269240
H	6.245790	2.745270	-0.200658
H	4.600339	2.256982	0.252904
H	5.058422	2.287206	-1.448965
H	2.506539	4.950527	-0.275249
H	0.995316	4.468574	-1.063809
H	2.264677	3.258432	-0.777283
H	2.923608	4.289838	1.999731
H	2.679672	2.563924	1.623484
H	1.712642	3.350527	2.881361
H	-1.828563	2.741803	4.235127
H	-0.073723	2.917553	4.385475
H	-0.744461	1.634690	3.350627
H	-1.811767	5.159280	3.453606
H	-0.994475	5.515635	1.922503
H	-0.042199	5.263914	3.394144
H	-1.636342	1.549480	-3.144502
H	-0.674246	2.699618	-2.223102
H	-1.481981	3.226595	-3.723274
H	-4.054225	1.740708	-3.125835
H	-4.083048	3.443379	-3.604880
H	-4.828958	2.930458	-2.076526
H	-2.085371	6.661866	-1.999051
H	-1.220405	6.119661	-0.544544
H	-2.524349	7.321020	-0.413426
H	-4.503729	5.826064	-2.280480
H	-4.818299	6.599464	-0.720673
H	-5.177665	4.883720	-0.933724
H	-5.322799	3.462600	1.720867
H	-4.197180	4.803722	1.435583
H	-3.676280	3.405919	2.389975
H	-4.928636	1.299266	0.504698
H	-3.323680	1.265101	1.278437
H	-3.443935	1.160215	-0.469263
H	2.124779	-3.949868	1.774790
H	1.715443	-2.830471	0.453408
H	1.524871	-4.586407	0.234520
H	-0.938670	-2.052296	4.841575
H	-0.078521	-0.864735	3.831017
H	0.338184	-2.582623	3.734363
H	-3.094645	-1.395026	3.957216
H	-3.336904	-1.363055	2.199819
H	-2.305796	-0.146616	2.970794
H	-0.768720	-5.860292	-1.186371
H	-0.670958	-4.102108	-1.014766
H	-1.307886	-4.818525	-2.513208
H	-2.964764	-6.946604	-1.067155
H	-3.539309	-5.956843	-2.415482
H	-4.413021	-5.949010	-0.870558
H	-5.207863	-5.454162	1.882376
H	-4.004989	-4.361279	2.597366
H	-3.503355	-5.582401	1.416175
H	-6.539095	-3.552841	1.031942
H	-5.711813	-2.288858	0.102644
H	-5.390803	-2.469516	1.835512
H	-2.859646	-1.637259	-3.091285
H	-2.554426	-1.010401	-1.457322
H	-4.083054	-0.636668	-2.290033
H	-5.720925	-2.454574	-2.636709
H	-5.431534	-4.021557	-1.876012
H	-4.527503	-3.558833	-3.332433
H	0.220218	-5.133265	2.899238
H	-0.519072	-5.669067	1.381781
H	-1.420822	-4.618600	2.497778

2, protonated form
Stoichiometry: $C_{30}H_{91}N_{21}P_7^+$
Charge: +1
Multiplicity: 1
Point group: C_1
DLPNO-CCSD(T), Hartree: -4730.2748395
Gibbs in HMPA, Hartree: -4729.1342591
Nuclear coordinates, Å:

C	2.413723	-2.753372	-2.742400
N	1.733118	-1.544509	-2.344721
P	2.469813	-0.279099	-1.509594
N	3.629848	-1.027702	-0.684216
P	4.840155	-0.561524	0.207815
N	5.891478	0.612720	-0.322302
C	6.963306	0.322300	-1.254763
C	0.592912	-1.234758	-3.176922
N	3.223495	0.766956	-2.610920
C	4.142661	0.153388	-3.548075
N	1.406519	0.627812	-0.766159
P	0.073072	0.254012	0.070882
N	-0.297388	1.373523	1.166348
P	-0.308436	2.958290	1.100167
N	1.194073	3.711764	1.074642
C	1.839875	4.124706	-0.151356
C	2.483336	1.858707	-3.210234
N	0.146511	-1.210851	0.722889
P	-0.897951	-2.307779	1.159921
N	0.007585	-3.690834	1.385989
C	1.321649	-3.875339	0.816819
N	-1.581622	-2.099307	2.689481
C	-0.642200	-1.912807	3.781848
N	-2.142448	-2.469986	0.147256
P	-3.261706	-3.428518	-0.368505
N	-4.496465	-3.923747	0.638102
C	-4.247223	-4.894346	1.689025
C	-2.753451	-1.259555	2.832409
N	-0.977952	3.427332	2.564777
C	-1.065443	2.556321	3.713328
N	-1.068803	3.689229	-0.115932
P	-2.549839	3.837343	-0.602315
N	-2.880108	5.466124	-0.745011
C	-1.818398	6.446263	-0.860362
C	-0.981550	4.836080	2.891181
N	5.826437	-1.895419	0.381074
C	7.011950	-1.828457	1.212228
N	4.318021	0.071432	1.663048
C	3.156288	-0.522043	2.304640
C	5.358602	-3.247269	0.160185
C	5.238495	0.654773	2.621281
C	5.503397	2.011915	-0.320776
N	-3.835171	3.285672	0.302161
C	-4.270690	4.014954	1.480987
N	-2.733829	2.983684	-2.027945
C	-1.588786	2.706344	-2.872014
C	-4.063026	1.855369	0.421296
C	2.129869	3.290742	2.094131
C	-3.991246	2.974339	-2.747749
C	-4.160549	5.945484	-1.225997
N	-4.075216	-2.607344	-1.568591
C	-3.562921	-1.401043	-2.178485
N	-2.635536	-4.892920	-0.882262
C	-1.354694	-4.896673	-1.564994
C	-5.113269	-3.262411	-2.337034
C	-3.468808	-6.017157	-1.268282
C	-5.617383	-3.053257	0.940299
C	-0.531616	-4.860424	2.035465

H	1.747700	-3.615410	-2.621717
H	3.292002	-2.907728	-2.122946
H	2.729454	-2.723783	-3.794227
H	0.124952	-0.306843	-2.851054
H	-0.153890	-2.032657	-3.104142
H	0.869591	-1.131760	-4.235496
H	4.864405	0.897027	-3.898695
H	3.630405	-0.248542	-4.433936
H	4.691817	-0.657592	-3.071536
H	3.178722	2.652089	-3.502326
H	1.775182	2.258753	-2.490611
H	1.937914	1.549800	-4.113764
H	2.632106	0.244508	2.880350
H	3.447723	-1.325159	2.994800
H	2.459642	-0.915593	1.570286
H	7.816808	-2.410895	0.753572
H	6.832445	-2.237393	2.213697
H	7.358546	-0.801953	1.314359
H	4.730644	1.456774	3.165066
H	6.107022	1.079304	2.123175
H	5.582286	-0.080271	3.359189
H	7.882600	0.821285	-0.932142
H	6.720969	0.681243	-2.259869
H	7.148510	-0.747847	-1.306357
H	5.176173	-3.765954	1.109352
H	6.110773	-3.816512	-0.395238
H	4.434309	-3.230779	-0.408238
H	6.345952	2.630386	0.004443
H	4.667637	2.176127	0.354838
H	5.198504	2.332850	-1.321542
H	2.486527	4.984221	0.053838
H	1.090952	4.423189	-0.881340
H	2.451358	3.323348	-0.581765
H	2.829089	4.105327	2.306117
H	2.703047	2.406316	1.787812
H	1.605265	3.050655	3.019491
H	-2.032752	2.683514	4.212330
H	-0.283645	2.776317	4.453848
H	-0.961827	1.523682	3.391751
H	-1.877302	5.081524	3.470068
H	-0.986578	5.437702	1.983568
H	-0.105657	5.124637	3.486842
H	-1.727680	1.736627	-3.359858
H	-0.684448	2.667198	-2.271032
H	-1.464837	3.462586	-3.657589
H	-4.118558	2.006292	-3.242123
H	-4.033471	3.749809	-3.522225
H	-4.827143	3.116724	-2.065534
H	-1.659374	6.750601	-1.902034
H	-0.890958	6.036163	-0.469545
H	-2.081825	7.339841	-0.286285
H	-4.133982	6.188392	-2.294912
H	-4.436703	6.855683	-0.685197
H	-4.939877	5.205362	-1.055776
H	-5.352365	3.903387	1.597559
H	-4.038128	5.072549	1.384011
H	-3.782403	3.633886	2.383978
H	-5.133492	1.670795	0.550739
H	-3.529646	1.436404	1.280931
H	-3.723291	1.339883	-0.473563
H	2.040217	-4.140422	1.601946
H	1.651011	-2.958589	0.334307
H	1.323882	-4.688313	0.079523
H	-1.137453	-2.146781	4.727942
H	-0.265622	-0.883558	3.831327
H	0.209054	-2.581969	3.664957
H	-3.286888	-1.533009	3.747825
H	-3.419381	-1.397925	1.984609
H	-2.493495	-0.195210	2.895623
H	-0.807224	-5.809309	-1.312373
H	-0.759350	-4.040156	-1.259962
H	-1.479304	-4.872583	-2.655196
H	-2.969416	-6.950591	-0.992352
H	-3.645756	-6.041630	-2.350283
H	-4.429568	-5.982075	-0.760655
H	-5.135178	-5.517424	1.829645
H	-4.022136	-4.402687	2.642393
H	-3.412075	-5.538975	1.427030
H	-6.538095	-3.642395	0.986292
H	-5.729272	-2.292387	0.171766
H	-5.489117	-2.555376	1.907992
H	-3.146651	-1.599001	-3.174131
H	-2.784866	-0.974804	-1.551018
H	-4.373455	-0.672681	-2.293194
H	-5.937187	-2.565300	-2.519289
H	-5.514888	-4.117649	-1.796668
H	-4.745436	-3.606653	-3.310958
H	0.181462	-5.239803	2.776065
H	-0.733725	-5.669962	1.322862
H	-1.453367	-4.611312	2.556720
H	-0.991226	0.249467	-0.869743

3, neutral form
Stoichiometry: $C_{48}H_{97}N_{16}P_5$
Charge: 0
Multiplicity: 1
Point group: C_1
DLPNO-CCSD(T), Hartree: -4463.4702276
Gibbs in HMPA, Hartree: -4462.1285905
Nuclear coordinates, Å:

P	1.017741	-0.008995	-0.656968
N	2.151818	-1.204130	-0.544952
N	0.258263	-0.026759	-2.050109
N	-0.055468	-0.227624	0.568833
N	1.871331	1.379410	-0.327552
C	0.831841	0.241935	-3.355307
C	0.423593	1.650252	-3.816651
C	2.361461	0.149873	-3.450401
C	0.251541	-0.772148	-4.348762
H	-0.665318	1.742832	-3.816444
H	0.825111	2.395796	-3.126833
H	0.788675	1.874091	-4.825218
H	2.708164	-0.844350	-3.165581
H	2.695217	0.356585	-4.472574
H	2.831693	0.870692	-2.779447
H	0.573982	-0.567768	-5.375181
H	0.569866	-1.782835	-4.080906
H	-0.840048	-0.747878	-4.317151
P	3.440736	-1.791157	0.045987
P	1.612079	2.815810	0.187803
P	-1.411752	-1.017539	0.623173
N	-2.723832	-0.189738	0.170282
P	-4.196893	-0.046237	-0.279187
N	-5.332737	-1.218475	0.088910
C	-6.184626	-1.148370	1.281460
C	-5.228857	-2.594743	-0.397309
C	-6.593838	-2.597376	1.542436
H	-5.662009	-0.721288	2.141107
C	-6.471194	-3.257912	0.175206
H	-5.200483	-2.622435	-1.488432
H	-5.893625	-3.062806	2.240286
H	-6.380379	-4.343236	0.225966
N	-4.328493	0.052333	-1.925918
C	-5.525881	-0.117327	-2.732052
C	-3.265928	0.662430	-2.738048
C	-4.946435	-0.463726	-4.094252
H	-6.174196	-0.887073	-2.315306
C	-3.735997	0.464317	-4.190172
H	-3.162739	1.728553	-2.512928
H	-4.629203	-1.509479	-4.097069
H	-2.945512	0.058135	-4.820354
N	-4.817854	1.327545	0.422560
C	-5.966909	2.053757	-0.108760
C	-4.282245	1.959425	1.622593
C	-6.259142	3.104462	0.958750
H	-5.719562	2.528474	-1.065807
C	-4.890421	3.353891	1.581447
H	-4.600110	1.432602	2.534156
H	-6.939084	2.693540	1.709940
H	-4.942250	3.818129	2.566750
N	-1.579149	-1.473817	2.239056
C	-2.757784	-2.227226	2.660831
C	-1.138947	-0.588260	3.309046
C	-3.000416	-1.811857	4.113815
H	-3.623442	-2.003145	2.031824
C	-1.643855	-1.283078	4.562665
H	-0.057817	-0.466030	3.284599
H	-3.375629	-2.632169	4.727452
H	-0.978533	-2.111378	4.821292
N	-1.528074	-2.526052	-0.114954
C	-0.534570	-3.548119	0.248136
C	-1.831414	-2.653594	-1.541821
C	-0.538468	-4.541754	-0.923500
H	0.459504	-3.112207	0.379668
C	-1.759739	-4.154523	-1.752971
H	-2.812047	-2.232988	-1.764277
H	-0.561410	-5.579502	-0.586700
H	-2.661091	-4.633363	-1.357840
H	-2.302118	0.197792	-2.518053
H	-4.034112	1.421406	-4.623668
H	-5.660104	-0.323208	-4.907376
H	-6.113466	0.810287	-2.798711
H	-4.317783	-3.082780	-0.030588
H	-7.341512	-3.019136	-0.442381

H	-7.592170	-2.671670	1.974487
H	-7.062288	-0.521341	1.088525
H	-2.569769	-3.303694	2.578540
H	-3.738095	-1.005146	4.155119
H	-1.702230	-0.614376	5.422894
H	-1.584067	0.415188	3.228048
H	-0.825132	-4.023288	1.190014
H	0.365532	-4.408723	-1.519716
H	-1.663937	-4.427188	-2.805261
H	-1.089980	-2.125231	-2.151460
H	-3.193165	1.955079	1.601055
H	-4.292574	4.000159	0.933213
H	-6.722244	4.000111	0.543708
H	-6.825540	1.397194	-0.282896
N	0.302440	3.640188	-0.423387
C	-0.989415	2.983088	-0.627980
C	0.194501	5.093272	-0.557048
C	-1.781755	4.034183	-1.387522
H	-0.863058	2.041061	-1.166675
C	-1.300026	5.334740	-0.756110
H	0.584259	5.625536	0.314046
H	-1.513815	4.005149	-2.447481
H	-1.497534	6.220188	-1.362123
N	2.849926	3.879525	-0.193443
C	3.286157	4.030357	-1.575607
C	3.956313	4.158184	0.724195
C	4.376725	5.081909	-1.468045
H	3.685049	3.091258	-1.982403
C	5.082243	4.681600	-0.176283
H	3.656058	4.906684	1.464374
H	5.040540	5.095485	-2.333354
H	5.795836	3.880894	-0.383171
N	1.523304	2.874405	1.851526
C	2.003382	1.774963	2.689545
C	0.858491	3.902141	2.633466
C	1.741326	2.262338	4.112178
H	1.450714	0.863285	2.452998
C	0.553582	3.203966	3.952830
H	1.505521	4.776716	2.802461
H	1.550557	1.439376	4.802449
H	-0.370404	2.628274	3.856727
H	-1.476428	2.750853	0.327787
H	-2.859899	3.888068	-1.305444
H	-1.788631	5.478751	0.212482
H	0.760221	5.447114	-1.426674
H	2.454631	4.332467	-2.215148
H	3.926448	6.073043	-1.367672
H	5.634229	5.500063	0.287093
H	4.278228	3.268844	1.274682
H	3.063802	1.567880	2.517593
H	2.605277	2.817961	4.488011
H	0.436300	3.902106	4.782987
H	-0.044799	4.254235	2.130867
N	3.237539	-2.607399	1.488206
C	2.334901	-2.110269	2.520120
C	3.760896	-3.906566	1.853691
C	2.035137	-3.335018	3.403987
H	2.800064	-1.314867	3.117766
C	2.610387	-4.525850	2.636985
H	4.031704	-4.476477	0.965060
H	2.533917	-3.235160	4.370829
H	2.928389	-5.340293	3.290143
N	4.696080	-0.740271	0.357892
C	5.612929	-0.811833	1.487020
C	5.119049	0.257276	-0.625762
C	6.428193	0.468817	1.358379
H	5.075259	-0.871261	2.435510
C	6.509665	0.665164	-0.150678
H	5.132451	-0.160543	-1.636979
H	7.402594	0.394656	1.843078
H	7.266058	-0.004770	-0.569892
N	4.087495	-2.915261	-1.006964
C	3.288119	-3.661730	-1.968778
C	5.510801	-3.197289	-1.154717
C	4.295175	-4.016605	-3.051566
H	2.868859	-4.576896	-1.524586
C	5.563931	-4.268467	-2.244887
H	6.060341	-2.300045	-1.460619
H	3.988387	-4.873797	-3.652084
H	5.522158	-5.263675	-1.793471
H	5.967112	-3.552546	-0.223757
H	6.479177	-4.211129	-2.835280
H	4.431936	-3.162909	-3.720572
H	2.457134	-3.048417	-2.317302
H	1.442776	-1.688868	2.050483
H	0.966873	-3.440035	3.595581
H	1.871714	-4.919956	1.935529
H	4.656840	-3.827342	2.490243
H	6.275330	-1.688096	1.430152
H	5.885094	1.300452	1.815559
H	6.771411	1.682646	-0.443551
H	4.416694	1.095522	-0.631512

3, protonated form
Stoichiometry: $C_{48}H_{98}N_{16}P_5^+$
Charge: +1
Multiplicity: 1
Point group: C_1
DLPNO-CCSD(T), Hartree: -4463.9781234
Gibbs in HMPA, Hartree: -4462.6466274
Nuclear coordinates, Å:

P	0.997725	-0.004772	-0.562169
N	2.050181	-1.215108	-0.620727
N	0.172735	-0.048480	-2.024770
N	-0.133150	-0.186739	0.570892
N	1.811211	1.382748	-0.387434
C	0.615030	0.322616	-3.375099
C	0.268415	1.785534	-3.662334
C	2.115001	0.120658	-3.542113
C	-0.119098	-0.578865	-4.366451
H	-0.811065	1.946214	-3.594451
H	0.755821	2.438690	-2.937228
H	0.586477	2.075629	-4.666686
H	2.397852	-0.913620	-3.347569
H	2.398733	0.374588	-4.565239
H	2.673925	0.760424	-2.859358
H	0.137519	-0.315046	-5.394895
H	0.144063	-1.624654	-4.199434
H	-1.202546	-0.483192	-4.257346
P	3.380731	-1.880647	-0.178768
P	1.702495	2.814350	0.222755
P	-1.480229	-1.021737	0.628060
N	-2.647404	-0.412772	-0.307837
P	-4.179985	-0.102194	-0.386770
H	-0.820833	0.082976	-1.884863
N	-5.180703	-1.329771	0.102752
C	-6.458089	-1.162927	0.796744
C	-4.994141	-2.716126	-0.346699
C	-6.816279	-2.584623	1.202429
H	-6.355495	-0.491043	1.650047
C	-6.303865	-3.406329	0.026286
H	-4.798655	-2.767765	-1.421847
H	-6.283131	-2.856292	2.116931
H	-6.161052	-4.460248	0.263526
N	-4.593100	0.223383	-1.954376
C	-5.860374	-0.128761	-2.594779
C	-3.782526	1.093285	-2.808816
C	-5.617678	0.218696	-4.056884
H	-6.100807	-1.182886	-2.450636
C	-4.711538	1.439877	-3.966766
H	-3.438777	1.979192	-2.268350
H	-5.094860	-0.603310	-4.552097
H	-4.166875	1.645443	-4.888045
N	-4.609452	1.234365	0.492837
C	-5.695684	2.148168	0.124294
C	-4.036603	1.576221	1.791251
C	-5.869693	3.026550	1.360346
H	-5.415810	2.745468	-0.750195
C	-4.488113	3.013230	2.002638
H	-4.427706	0.932059	2.590526
H	-6.598243	2.576070	2.039063
H	-4.499395	3.298636	3.054485
N	-1.854264	-1.099447	2.246526
C	-2.930203	-1.930569	2.782823
C	-1.138504	-0.398756	3.307903
C	-2.975077	-1.567096	4.264088
H	-3.879593	-1.733900	2.279372
C	-1.537792	-1.160898	4.562890
H	-0.066885	-0.400490	3.117622
H	-3.335164	-2.389622	4.882148
H	-0.911299	-2.050251	4.674977
N	-1.417886	-2.644643	0.197902
C	-0.464267	-3.531020	0.858483
C	-1.476721	-3.093993	-1.189690
C	-0.764540	-4.909732	0.255257
H	0.565772	-3.230332	0.633888
C	-1.520708	-4.613920	-1.054591
H	-2.344324	-2.672444	-1.693853
H	-1.378332	-5.507599	0.929716
H	-2.554432	-4.955970	-0.981028
H	-2.896047	0.557451	-3.160985
H	-5.303045	2.326303	-3.723616
H	-6.544665	0.404440	-4.598755
H	-6.697353	0.461209	-2.199189

H	-4.136844	-3.162979	0.163983
H	-7.012528	-3.347148	-0.803662
H	-7.883513	-2.708757	1.384527
H	-7.233687	-0.752561	0.136517
H	-2.705152	-2.995054	2.647197
H	-3.641260	-0.714903	4.423974
H	-1.435853	-0.562216	5.468440
H	-1.454253	0.650993	3.381304
H	-0.598144	-3.498894	1.941067
H	0.156997	-5.467145	0.082269
H	-1.076740	-5.108225	-1.919140
H	-0.581835	-2.779782	-1.741312
H	-2.952792	1.459116	1.771689
H	-3.817010	3.695300	1.474082
H	-6.225681	4.025504	1.109374
H	-6.619513	1.618756	-0.122712
N	0.399844	3.720727	-0.266677
C	-0.966737	3.209597	-0.292526
C	0.428508	5.177538	-0.444523
C	-1.720430	4.298764	-1.038015
H	-1.009659	2.234193	-0.775848
C	-1.045634	5.568406	-0.533418
H	0.929423	5.689748	0.381076
H	-1.563862	4.187837	-2.114264
H	-1.203372	6.433492	-1.177238
N	3.010982	3.719135	-0.243371
C	3.422492	3.833321	-1.642729
C	3.859358	4.517263	0.637661
C	4.469309	4.941894	-1.624233
H	3.846219	2.888893	-1.999771
C	5.072355	4.823980	-0.230131
H	3.367039	5.450577	0.943236
H	5.200607	4.835976	-2.425286
H	5.775830	3.988013	-0.192714
N	1.691610	2.807668	1.877340
C	2.381851	1.782151	2.663001
C	1.110418	3.839924	2.729688
C	2.338370	2.323961	4.088581
H	1.852651	0.830193	2.573581
C	1.078316	3.180970	4.101564
H	1.730738	4.746156	2.758397
H	2.328914	1.528596	4.834121
H	0.191839	2.547596	4.186528
H	-1.371402	3.085298	0.721348
H	-2.793947	4.276753	-0.846564
H	-1.427073	5.820918	0.459514
H	0.960376	5.441340	-1.364144
H	2.577026	4.074275	-2.293171
H	3.987354	5.916136	-1.739560
H	5.599008	5.721911	0.092604
H	4.121257	3.970118	1.544686
H	3.405058	1.623302	2.310957
H	3.213542	2.949558	4.282532
H	1.056278	3.907457	4.913793
H	0.118563	4.127648	2.376787
N	3.268730	-2.696648	1.262805
C	2.664301	-2.094213	2.450737
C	3.815398	-4.015540	1.555718
C	2.805419	-3.168433	3.533569
H	3.173953	-1.170028	2.742879
C	2.990055	-4.464574	2.751707
H	3.715733	-4.677642	0.695389
H	3.692760	-2.974595	4.140844
H	3.479692	-5.247989	3.330264
N	4.677818	-0.866827	0.002374
C	5.741066	-1.010671	0.994741
C	5.018105	0.141358	-1.007746
C	6.549576	0.267215	0.822381
H	5.334155	-1.117257	2.001311
C	6.457965	0.520991	-0.676950
H	4.920908	-0.260346	-2.020271
H	7.572338	0.164999	1.185093
H	7.155200	-0.133385	-1.206557
N	3.815190	-2.999718	-1.320325
C	2.858788	-3.768596	-2.110765
C	5.194497	-3.406811	-1.596627
C	3.707979	-4.291570	-3.258474
H	2.435941	-4.604363	-1.534980
C	5.044659	-4.566930	-2.580237
H	5.758793	-2.581015	-2.042459
H	3.277436	-5.172760	-3.734086
H	4.995211	-5.513691	-2.036321
H	5.727523	-3.717433	-0.692978
H	5.882633	-4.628712	-3.274208
H	3.820112	-3.515315	-4.019850
H	2.032249	-3.131971	-2.426014
H	1.621032	-1.838304	2.246188
H	1.948082	-3.190244	4.206638
H	2.025445	-4.846516	2.409126
H	4.880352	-3.971383	1.825359
H	6.373302	-1.886677	0.797808
H	6.074297	1.083376	1.373324
H	6.691761	1.547922	-0.958963
H	4.337658	0.991794	-0.919462

4, neutral form
Stoichiometry: $C_{32}H_{52}BNP_2$
Charge: 0
Multiplicity: 1
Point group: C_2
DLPNO-CCSD(T), Hartree: -2009.6954002
Gibbs in HMPA, Hartree: -2008.9986174
Nuclear coordinates, Å:

C	0.025079	1.247560	1.114740
C	-0.061152	2.319168	0.187429
C	0.189120	1.591838	2.462332
C	-0.025256	3.647350	0.612882
C	0.219005	2.915792	2.852710
H	0.337644	0.836970	3.216759
C	0.102508	3.982257	1.951188
H	-0.084377	4.430569	-0.132383
H	0.352724	3.122722	3.908585
C	-0.025079	-1.247560	1.114740
C	-0.189120	-1.591838	2.462332
C	0.061152	-2.319168	0.187429
C	-0.219005	-2.915792	2.852710
H	-0.337644	-0.836970	3.216759
C	0.025256	-3.647350	0.612882
C	-0.102508	-3.982257	1.951188
H	-0.352724	-3.122722	3.908585
H	0.084377	-4.430569	-0.132383
N	0.000000	0.000000	0.543623
B	0.000000	0.000000	-0.975874
P	0.140344	-1.745003	-1.507592
P	-0.140344	1.745003	-1.507592
C	0.138499	5.420616	2.456018
C	1.469349	5.683766	3.171096
C	-1.015125	5.646175	3.440907
C	0.000000	6.432936	1.322083
H	2.310992	5.543787	2.489259
H	1.611685	5.010675	4.018188
H	1.502807	6.709068	3.549735
H	-1.978969	5.480823	2.954563
H	-0.996039	6.670480	3.823276
H	-0.952888	4.969464	4.294827
H	0.023523	7.445967	1.729879
H	-0.945126	6.314486	0.786913
H	0.816651	6.349126	0.601180
C	-0.138499	-5.420616	2.456018
C	-1.469349	-5.683766	3.171096
C	1.015125	-5.646175	3.440907
C	0.000000	-6.432936	1.322083
H	-2.310992	-5.543787	2.489259
H	-1.611685	-5.010675	4.018188
H	-1.502807	-6.709068	3.549735
H	1.978969	-5.480823	2.954563
H	0.996039	-6.670480	3.823276
H	0.952888	-4.969464	4.294827
H	-0.023523	-7.445967	1.729879
H	0.945126	-6.314486	0.786913
H	-0.816651	-6.349126	0.601180
C	1.194170	2.549286	-2.535336
C	2.545916	2.395745	-1.859137
C	1.200585	1.947534	-3.935139
H	0.944395	3.612696	-2.598182
H	2.557489	2.852624	-0.868891
H	3.324697	2.866187	-2.465662
H	2.794286	1.339060	-1.738374
H	0.260862	2.107532	-4.467380
H	1.383151	0.869676	-3.884971
H	2.001012	2.386796	-4.535907
C	-1.194170	-2.549286	-2.535336
C	-2.545916	-2.395745	-1.859137
C	-1.200585	-1.947534	-3.935139
H	-0.944395	-3.612696	-2.598182
H	-2.557489	-2.852624	-0.868891
H	-3.324697	-2.866187	-2.465662
H	-2.794286	-1.339060	-1.738374
H	-0.260862	-2.107532	-4.467380
H	-1.383151	-0.869676	-3.884971
H	-2.001012	-2.386796	-4.535907
C	1.721127	-2.320618	-2.346530
C	2.902437	-1.984063	-1.448645
C	1.752827	-3.781890	-2.776065

H	1.774821	-1.685673	-3.239668
H	2.847178	-0.952730	-1.095892
H	3.842692	-2.120201	-1.988987
H	2.918289	-2.634003	-0.570759
H	0.981384	-4.027544	-3.506565
H	1.636743	-4.449520	-1.919057
H	2.719294	-4.013509	-3.232792
C	-1.721127	2.320618	-2.346530
C	-2.902437	1.984063	-1.448645
C	-1.752827	3.781890	-2.776065
H	-1.774821	1.685673	-3.239668
H	-2.847178	0.952730	-1.095892
H	-3.842692	2.120201	-1.988987
H	-2.918289	2.634003	-0.570759
H	-0.981384	4.027544	-3.506565
H	-1.636743	4.449520	-1.919057
H	-2.719294	4.013509	-3.232792

4, protonated form
Stoichiometry: $C_{32}H_{53}BNP_2^+$
Charge: +1
Multiplicity: 1
Point group: C_1
DLPNO-CCSD(T), Hartree: -2010.1829416
Gibbs in HMPA, Hartree: -2009.5167613
Nuclear coordinates, Å:

C	-1.135077	-0.982646	-0.387098
C	-2.275292	-0.161811	-0.289638
C	-1.311268	-2.345467	-0.120085
C	-3.531627	-0.681785	0.018857
C	-2.564569	-2.834937	0.186853
H	-0.470720	-3.023209	-0.128865
C	-3.712685	-2.034870	0.255244
H	-4.375913	-0.007164	0.085168
H	-2.649894	-3.896378	0.388476
C	1.334303	-0.898006	-0.604850
C	1.730923	-2.154261	-1.060339
C	2.319991	-0.057603	-0.063184
C	3.051005	-2.546228	-0.936291
H	1.024597	-2.808362	-1.552353
C	3.645169	-0.467711	0.044682
C	4.041801	-1.729191	-0.381528
H	3.322979	-3.526051	-1.310467
H	4.378477	0.213248	0.457094
N	0.054617	-0.349476	-0.696557
B	0.058271	1.184178	-0.711122
P	1.617118	1.523481	0.441708
P	-1.854935	1.553495	-0.562846
C	-5.064894	-2.655642	0.585273
C	-4.996482	-3.327170	1.962289
C	-5.411150	-3.707576	-0.475703
C	-6.180865	-1.615131	0.612592
H	-4.761615	-2.600288	2.742897
H	-4.239203	-4.112256	1.995407
H	-5.957555	-3.785929	2.204777
H	-5.478218	-3.257314	-1.468346
H	-6.374164	-4.169370	-0.246966
H	-4.665310	-4.503175	-0.517865
H	-7.128460	-2.103204	0.846113
H	-6.300083	-1.118988	-0.353736
H	-6.011008	-0.853098	1.377322
C	5.480104	-2.225825	-0.287386
C	6.001910	-2.541983	-1.694538
C	5.519597	-3.498242	0.567883
C	6.404821	-1.190877	0.347264
H	5.992323	-1.651700	-2.326899
H	5.404734	-3.310739	-2.187616
H	7.029632	-2.907363	-1.639427
H	5.161383	-3.302918	1.580936
H	6.543594	-3.871551	0.636921
H	4.906272	-4.294071	0.142089
H	7.417970	-1.592547	0.399581
H	6.100969	-0.941809	1.367025
H	6.451094	-0.269413	-0.238236
C	-2.621191	2.648086	0.705068
C	-2.367971	2.171472	2.126815
C	-2.192440	4.097130	0.501419
H	-3.693881	2.565217	0.501577
H	-2.655543	1.129959	2.271027
H	-2.950738	2.781714	2.819768
H	-1.318149	2.279150	2.401129
H	-2.419405	4.469139	-0.498475
H	-1.120851	4.218616	0.674971
H	-2.714067	4.736466	1.216119
C	2.713228	2.907039	-0.077296
C	3.043125	2.861903	-1.563902
C	2.119155	4.251247	0.331151
H	3.637480	2.743907	0.486850
H	3.439473	1.893684	-1.870191
H	3.796535	3.620959	-1.783904
H	2.165626	3.077730	-2.173780
H	1.920080	4.320194	1.401944
H	1.188762	4.447427	-0.206722
H	2.816376	5.051442	0.075966
C	1.494639	1.545313	2.287356
C	0.774683	0.290196	2.769996
C	2.848901	1.699965	2.969364
H	0.881415	2.423854	2.514515
H	-0.187090	0.137710	2.278928
H	0.594992	0.363926	3.844311
H	1.385415	-0.596634	2.593589
H	3.348254	2.635823	2.717215
H	3.514778	0.871136	2.720592
H	2.707832	1.687317	4.052223
C	-2.469695	2.116919	-2.214470
C	-2.088569	1.099244	-3.284394
C	-3.967335	2.396896	-2.232590
H	-1.921038	3.046851	-2.401229
H	-1.020823	0.880948	-3.291746
H	-2.358261	1.492815	-4.266128
H	-2.628342	0.161865	-3.138834
H	-4.263094	3.196240	-1.552650
H	-4.541945	1.500817	-1.987571
H	-4.261449	2.701707	-3.238895
H	0.435879	1.653786	-1.757924

5, neutral form
Stoichiometry: C_5H_5N
Charge: 0
Multiplicity: 1
Point group: C_{2v}
DLPNO-CCSD(T), Hartree: -247.7298841
Gibbs in HMPA, Hartree: -247.6867668
Nuclear coordinates, Å:

C	0.000000	1.175869	0.609315
C	0.000000	1.176765	-0.760220
C	0.000000	0.000000	-1.556450
C	0.000000	-1.176765	-0.760220
C	0.000000	-1.175869	0.609315
N	0.000000	0.000000	1.269749
H	0.000000	2.064138	1.231571
H	0.000000	2.153953	-1.238840
H	0.000000	-2.153953	-1.238840
H	0.000000	-2.064138	1.231571
H	0.000000	0.000000	2.275849

5, protonated form
Stoichiometry: $C_5H_6N^+$
Charge: +1
Multiplicity: 1
Point group: C_{2v}
DLPNO-CCSD(T), Hartree: -248.1971199
Gibbs in HMPA, Hartree: -248.2004975
Nuclear coordinates, Å:

C	0.000000	1.180259	0.662680
C	0.000000	1.203804	-0.711929
C	0.000000	0.000000	-1.405721
C	0.000000	-1.203804	-0.711929
C	0.000000	-1.180259	0.662680
N	0.000000	0.000000	1.299588
H	0.000000	0.000000	-2.488847
H	0.000000	2.067873	1.280205
H	0.000000	2.153912	-1.227906
H	0.000000	-2.153912	-1.227906
H	0.000000	-2.067873	1.280205
H	0.000000	0.000000	2.312454

7, neutral form
Stoichiometry: $C_{24}H_{42}N_8O_2P_2As_2$
Charge: 0
Multiplicity: 1
Point group: C_2
DLPNO-CCSD(T), Hartree: -2870.2402429
Gibbs in HMPA, Hartree: -2869.7167880
Gibbs in gas, Hartree: -2869.6642489
Nuclear coordinates, Å:

C	0.689191	3.626967	1.273597
C	0.329699	2.858648	0.152301
C	0.160911	1.425653	0.229615
C	0.138077	0.687946	1.428075
C	0.990197	3.173351	2.542479
C	0.041664	0.677986	-0.973137
C	-0.138077	-0.687946	1.428075
C	-0.160911	-1.425653	0.229615

C	-0.041664	-0.677986	-0.973137
C	-0.329699	-2.858648	0.152301
C	-0.689191	-3.626967	1.273597
C	-0.990197	-3.173351	2.542479
H	0.047786	1.224956	-1.905216
H	0.820380	4.689456	1.086947
H	1.329088	3.879005	3.292383
H	-0.047786	-1.224956	-1.905216
H	-0.820380	-4.689456	1.086947
H	-1.329088	-3.879005	3.292383
As	-0.429067	-1.518913	3.118310
As	0.429067	1.518913	3.118310
O	-1.343592	-0.638163	4.162601
O	1.343592	0.638163	4.162601
P	-0.097729	4.915069	-1.490605
N	-0.556579	4.855865	-3.095751
N	1.229116	5.906293	-1.219146
N	-1.343592	5.791792	-0.800695
C	-0.113678	3.786791	-3.965712
C	-1.019503	6.026290	-3.806561
C	2.549403	5.308324	-1.143861
C	1.251938	7.283351	-1.668719
C	-1.193409	6.534561	0.437040
C	-2.694428	5.285229	-0.983304
H	0.707553	4.112095	-4.617203
H	0.220551	2.944852	-3.366774
H	-0.942212	3.462928	-4.604069
H	-1.403522	6.771116	-3.112360
H	-0.227478	6.484928	-4.412479
H	-1.832394	5.744517	-4.484031
H	3.063111	5.333295	-2.113444
H	3.159879	5.860772	-0.423561
H	2.478027	4.274368	-0.815141
H	1.682544	7.382036	-2.673458
H	0.247786	7.703769	-1.680264
H	1.861614	7.882577	-0.985388
H	-1.492226	5.936304	1.304720
H	-0.161939	6.847941	0.575689
H	-1.827089	7.426678	0.403963
H	-3.395964	6.122873	-1.039289
H	-2.762480	4.710861	-1.904992
H	-2.995380	4.639528	-0.151018
P	0.097729	-4.915069	-1.490605
N	0.556579	-4.855865	-3.095751
N	-1.229116	-5.906293	-1.219146
N	1.343592	-5.791792	-0.800695
C	0.113678	-3.786791	-3.965712
C	1.019503	-6.026290	-3.806561
C	-2.549403	-5.308324	-1.143861
C	-1.251938	-7.283351	-1.668719
C	1.193409	-6.534561	0.437040
C	2.694428	-5.285229	-0.983304
H	-0.707553	-4.112095	-4.617203
H	-0.220551	-2.944852	-3.366774
H	0.942212	-3.462928	-4.604069
H	1.403522	-6.771116	-3.112360
H	0.227478	-6.484928	-4.412479
H	1.832394	-5.744517	-4.484031
H	-3.063111	-5.333295	-2.113444
H	-3.159879	-5.860772	-0.423561
H	-2.478027	-4.274368	-0.815141
H	-1.682544	-7.382036	-2.673458
H	-0.247786	-7.703769	-1.680264
H	-1.861614	-7.882577	-0.985388
H	1.492226	-5.936304	1.304720
H	0.161939	-6.847941	0.575689
H	1.827089	-7.426678	0.403963
H	3.395964	-6.122873	-1.039289
H	2.762480	-4.710861	-1.904992
H	2.995380	-4.639528	-0.151018
N	0.147937	3.420959	-1.077530
N	-0.147937	-3.420959	-1.077530

7a, isomer of **7**

Stoichiometry: $C_{24}H_{42}N_8O_2P_2As_2$

Charge: 0

Multiplicity: 1

Point group: C_1

DLPNO-CCSD(T), Hartree: -2870.2897622

Gibbs in HMPA, Hartree: -2869.7447624

Gibbs in gas, Hartree: -2869.7131842

Nuclear coordinates, Å:

C	-3.223024	1.127759	0.850826
C	-2.627308	0.068982	0.053379
C	-1.344921	0.198175	-0.487018
C	-0.640745	1.435035	-0.464265
C	-2.852162	2.414021	1.064854
C	-0.569229	-0.919132	-0.949534
C	0.712517	1.511280	-0.541237
C	1.506105	0.339620	-0.688844
C	0.783199	-0.850956	-1.043660
C	2.848046	0.317410	-0.300465
C	3.391465	1.400744	0.511353
C	2.846256	2.591018	0.841344
H	-1.082573	-1.852423	-1.140814
H	-4.120226	0.808794	1.376489
H	-3.441354	3.065871	1.695609
H	1.354771	-1.735660	-1.294160
H	4.369699	1.176664	0.931158
H	3.405636	3.263248	1.483074
As	1.414055	3.300898	-0.227632
As	-1.470678	3.086421	-0.017665
O	-0.026103	3.712032	0.836545
O	-1.946840	4.148031	-1.150987
P	-4.756852	-1.571117	0.025360
N	-5.451051	-1.377482	1.548518
N	-4.805063	-3.156974	-0.495237
N	-5.905406	-0.827169	-0.949568
C	-4.597402	-1.575115	2.704659
C	-6.847150	-1.644076	1.826780
C	-3.624524	-3.990263	-0.545078
C	-6.048970	-3.889891	-0.533786
C	-5.770906	-1.013632	-2.386006
C	-6.378369	0.509604	-0.623865
H	-4.671234	-2.598788	3.095165
H	-3.559580	-1.379394	2.442109
H	-4.892298	-0.888348	3.504048
H	-7.453310	-1.489085	0.936763
H	-7.008323	-2.669205	2.185055
H	-7.202737	-0.961891	2.605928
H	-3.607374	-4.711285	0.282409
H	-3.605891	-4.554359	-1.484094
H	-2.739262	-3.363703	-0.485099
H	-6.179918	-4.534482	0.345387
H	-6.895563	-3.208238	-0.596059
H	-6.071028	-4.532258	-1.420546
H	-5.085577	-0.281912	-2.829105
H	-5.397659	-2.011840	-2.604878
H	-6.750648	-0.894205	-2.856904
H	-7.374480	0.651308	-1.053621
H	-6.445947	0.644110	0.452965
H	-5.719107	1.286498	-1.027318
P	4.916493	-1.413679	-0.011627
N	6.238071	-0.377832	-0.081247
N	5.138444	-2.816704	-0.886810
N	5.011484	-1.962251	1.574823
C	6.294320	0.582302	-1.168718
C	7.549709	-0.738248	0.418368
C	4.487411	-3.042820	-2.159862
C	6.282955	-3.667592	-0.652252
C	4.153621	-3.078220	1.942002
C	5.138393	-1.003862	2.660609
H	6.885655	0.204652	-2.012790
H	5.292835	0.813724	-1.523899
H	6.758985	1.508328	-0.817805
H	7.468799	-1.456166	1.231851
H	8.185133	-1.168973	-0.365984
H	8.054017	0.155672	0.799093
H	5.177033	-2.883045	-2.998283
H	4.128022	-4.076081	-2.211346
H	3.644296	-2.365387	-2.261638
H	7.088428	-3.488385	-1.376039
H	6.675974	-3.517271	0.351754
H	5.985559	-4.717622	-0.742519
H	3.148879	-2.743619	2.225792
H	4.061318	-3.774299	1.111317
H	4.593816	-3.603912	2.793753
H	5.589773	-1.500152	3.524496
H	5.776582	-0.172623	2.370285
H	4.165627	-0.602721	2.969096
N	-3.256891	-1.149761	-0.037042
N	3.592555	-0.792466	-0.556841

8, neutral form

Stoichiometry: $C_{13}H_{18}N_6$

Charge: 0

Multiplicity: 1

Point group: C_2

DLPNO-CCSD(T), Hartree: -833.2491703

Gibbs in HMPA, Hartree: -833.0079007

Nuclear coordinates, Å:

C	-0.142776	1.161635	1.626636
C	-0.049942	2.422298	-0.584040
C	0.817829	2.296254	-1.814263

C	0.281947	1.179505	-2.665813
H	-1.088991	2.596601	-0.898413
H	0.811981	3.230221	-2.377613
H	1.847918	2.095572	-1.507778
H	-0.636196	1.492822	-3.177477
H	0.998489	0.890387	-3.441714
C	0.000000	0.000000	-0.514236
C	0.049942	-2.422298	-0.584040
C	-0.281947	-1.179505	-2.665813
C	-0.817829	-2.296254	-1.814263
H	1.088991	-2.596601	-0.898413
H	-0.998489	-0.890387	-3.441714
H	0.636196	-1.492822	-3.177477
H	-0.811981	-3.230221	-2.377613
H	-1.847918	-2.095572	-1.507778
C	0.367400	3.555008	0.322368
C	-0.469606	3.508456	1.581521
H	-0.148697	4.293970	2.272937
H	-1.515690	3.749720	1.330147
H	1.425889	3.446512	0.579465
H	0.245656	4.497945	-0.217820
C	0.142776	-1.161635	1.626636
C	-0.367400	-3.555008	0.322368
H	-1.425889	-3.446512	0.579465
H	-0.245656	-4.497945	-0.217820
C	0.469606	-3.508456	1.581521
H	0.148697	-4.293970	2.272937
H	1.515690	-3.749720	1.330147
N	-0.385688	2.249950	2.253672
N	0.385688	-2.249950	2.253672
N	0.000000	0.000000	-1.867740
N	0.000000	1.158271	0.155808
N	0.000000	-1.158271	0.155808
N	0.000000	0.000000	2.243787

8a, tautomer of **8**
Stoichiometry: $C_{13}H_{18}N_6$
Charge: 0
Multiplicity: 1
Point group: C_1
DLPNO-CCSD(T), Hartree: -833.2661119
Gibbs in HMPA, Hartree: -833.0083066
Nuclear coordinates, Å:

C	-0.900128	-1.545082	-0.114708
C	-2.366692	0.509636	-0.045949
C	-2.511406	1.342146	1.195268
C	-2.854469	2.618945	1.206725
H	-2.388406	1.167024	-0.917112
H	-2.369004	0.802378	2.129340
H	-2.977559	3.176129	0.284655
H	-3.007856	3.155008	2.135195
C	0.077800	0.614123	-0.220905
C	2.474024	0.811255	0.088777
C	1.065181	2.680819	-0.716586
C	2.351201	1.912472	-0.945734
H	2.516282	1.271488	1.085196
H	0.867678	3.355542	-1.554371
H	1.164177	3.324512	0.166768
H	3.220389	2.571618	-0.884233
H	2.349022	1.460476	-1.942508
C	-3.528583	-0.483817	-0.103723
C	-3.182544	-1.703946	-0.923746
H	-3.994721	-2.430666	-0.894076
H	-3.013146	-1.435662	-1.976028
H	-3.778631	-0.816302	0.906881
H	-4.399836	0.037164	-0.502139
C	1.361110	-1.418388	0.266928
C	3.700036	-0.054682	-0.092486
H	3.749149	-0.404135	-1.128768
H	4.592017	0.547180	0.097362
C	3.620512	-1.249330	0.836530
H	4.478479	-1.909335	0.682272
H	3.686121	-0.912574	1.881286
N	-2.014181	-2.294401	-0.320154
N	2.419962	-2.018588	0.640995
N	-0.087033	1.830825	-0.547621
N	-1.066751	-0.182871	-0.067964
N	1.272474	-0.027900	0.033946
N	0.227626	-2.150252	0.022745
H	-1.795098	-3.262436	-0.485706

8, protonated form
Stoichiometry: $C_{13}H_{19}N_6^+$
Charge: +1
Multiplicity: 1
Point group: C_1
DLPNO-CCSD(T), Hartree: -833.7022887
Gibbs in HMPA, Hartree: -833.4834284
Nuclear coordinates, Å:

C	-1.112868	-1.509693	-0.133646
C	-2.429339	0.627301	-0.151023
C	-2.306646	1.908407	0.637051
C	-1.150003	2.705557	0.105367
H	-2.592071	0.863833	-1.209722
H	-3.225903	2.487051	0.545112
H	-2.164577	1.675420	1.695621
H	-1.380698	3.128917	-0.877597
H	-0.908785	3.538167	0.769656
C	0.040891	0.554174	-0.001668
C	2.476661	0.549371	0.165320
C	1.277139	2.670777	-0.136534
C	2.411534	1.824551	-0.639958
H	2.619017	0.791976	1.225234
H	1.058500	3.491190	-0.823774
H	1.513084	3.112553	0.836550
H	3.349727	2.370839	-0.543043
H	2.277940	1.581972	-1.697420
C	-3.566189	-0.242365	0.340951
C	-3.586618	-1.550679	-0.409229
H	-4.313982	-2.235540	0.027084
H	-3.872093	-1.392082	-1.455654
H	-3.451790	-0.426013	1.412699
H	-4.502377	0.296558	0.191502
C	1.172910	-1.600963	0.122675
C	3.590879	-0.369661	-0.281737
H	3.511178	-0.543309	-1.359063
H	4.543716	0.130418	-0.098121
C	3.503437	-1.686383	0.455134
H	4.243280	-2.391515	0.072210
H	3.738784	-1.553000	1.518355
N	-2.278146	-2.148488	-0.314182
N	2.205975	-2.290201	0.330123
N	0.051534	1.886424	-0.005880
N	-1.151079	-0.109369	-0.045556
N	1.178572	-0.149815	0.049027
N	-0.036268	-2.197600	-0.052320
H	-2.167779	-3.146473	-0.394328

9, neutral form
Stoichiometry: $C_{12}H_{24}N_4$
Charge: 0
Multiplicity: 1
Point group: T
DLPNO-CCSD(T), Hartree: -689.4343057
Gibbs in HMPA, Hartree: -689.1008377
Nuclear coordinates, Å:

N	-1.041590	1.041590	1.041590
N	1.041590	-1.041590	1.041590
N	-1.041590	-1.041590	-1.041590
N	1.041590	1.041590	-1.041590
C	0.312337	0.704311	-2.222848
H	-0.485441	1.442197	-2.341389
H	0.923701	0.787301	-3.139833
C	-0.704311	2.222848	0.312337
H	-1.442197	2.341389	-0.485441
H	-0.787301	3.139833	0.923701
C	-2.222848	0.312337	0.704311
H	-2.341389	-0.485441	1.442197
H	-3.139833	0.923701	0.787301
C	-0.312337	0.704311	2.222848
H	0.485441	1.442197	2.341389
H	-0.923701	0.787301	3.139833
C	2.222848	-0.312337	0.704311
H	2.341389	0.485441	1.442197
H	3.139833	-0.923701	0.787301
C	0.312337	-0.704311	2.222848
H	-0.485441	-1.442197	2.341389
H	0.923701	-0.787301	3.139833
C	0.704311	-2.222848	0.312337
H	0.787301	-3.139833	0.923701
H	1.442197	-2.341389	-0.485441
C	-0.312337	-0.704311	-2.222848
H	-0.923701	-0.787301	-3.139833
H	0.485441	-1.442197	-2.341389
C	-0.704311	-2.222848	-0.312337
H	-1.442197	-2.341389	0.485441
H	-0.787301	-3.139833	-0.923701
C	-2.222848	-0.312337	-0.704311
H	-3.139833	-0.923701	-0.787301
H	-2.341389	0.485441	-1.442197
C	0.704311	2.222848	-0.312337
H	0.787301	3.139833	-0.923701
H	1.442197	2.341389	0.485441
C	2.222848	0.312337	-0.704311

H 2.341389 -0.485441 -1.442197
H 3.139833 0.923701 -0.787301

9a, tautomer of **9**
Stoichiometry: $C_{12}H_{24}N_4$
Charge: 0
Multiplicity: 1
Point group: C_1
DLPNO-CCSD(T), Hartree: -689.4373722
Gibbs in HMPA, Hartree: -689.1154233
Nuclear coordinates, Å:
H 0.031281 -0.271742 -1.181091
N -1.644291 0.750220 -0.036550
N 0.350458 -1.143075 1.150145
N 0.640922 -0.861595 -1.737829
N 1.305733 1.285094 0.076670
C 1.850008 1.220304 -1.252789
H 1.186611 1.784168 -1.915427
H 2.843197 1.694243 -1.333297
C -1.064206 2.068752 -0.205075
H -1.006906 2.249334 -1.281594
H -1.717729 2.857826 0.202000
C -2.547795 0.335482 -0.987011
H -2.428930 0.847361 -1.937145
C -1.745445 0.182778 1.287591
H -1.316709 0.888736 1.999728
H -2.800980 0.071650 1.571060
C 1.195198 -0.324550 1.964424
H 0.563846 0.234578 2.659422
H 1.877720 -0.913252 2.600121
C -1.052803 -1.178669 1.434179
H -1.519417 -1.879646 0.739766
H -1.278991 -1.551009 2.451089
C 0.953253 -2.200767 0.384195
H 0.707460 -3.201163 0.780384
H 2.036498 -2.100615 0.492932
C 1.939428 -0.213550 -1.778279
H 2.328106 -0.177806 -2.803904
H 2.690092 -0.763021 -1.194733
C 0.587054 -2.167284 -1.113911
H -0.434207 -2.539631 -1.241969
H 1.234351 -2.871175 -1.648177
C -3.487263 -0.611475 -0.880522
H -4.095131 -0.843930 -1.742537
H -3.669254 -1.177838 0.022364
C 0.325916 2.272485 0.409799
H 0.633636 3.295187 0.128322
H 0.235628 2.293542 1.498686
C 2.040323 0.669924 1.142725
H 2.882445 0.133985 0.700757
H 2.485614 1.405127 1.834236

9, protonated form
Stoichiometry: $C_{12}H_{25}N_4^+$
Charge: +1
Multiplicity: 1
Point group: C_3
DLPNO-CCSD(T), Hartree: -689.8614128
Gibbs in HMPA, Hartree: -689.5758912
Nuclear coordinates, Å:
H 0.000000 0.000000 0.464300
N 1.175570 1.152255 -0.560379
N 0.410097 -1.594201 -0.560379
N 0.000000 0.000000 1.548368
N -1.585667 0.441946 -0.560379
C -2.128162 0.864575 0.700301
H -1.962736 1.938921 0.804206
H -3.211385 0.712788 0.789286
C 0.205972 2.101712 -1.055125
H 0.000000 2.817479 -0.254738
H 0.589535 2.699915 -1.892789
C 1.812825 1.410755 0.700301
H 2.660523 0.730319 0.804206
H 2.222985 2.424747 0.789286
C 1.799983 0.245003 -1.494127
H 1.290698 0.362381 -2.452381
H 2.852741 0.493194 -1.685018
C -0.687812 -1.681333 -1.494127
H -0.331518 -1.298968 -2.452381
H -0.999252 -2.717143 -1.685018
C 1.717150 -1.229233 -1.055125
H 2.440008 -1.408739 -0.254738
H 2.043428 -1.860510 -1.892789
C 0.315337 -2.275330 0.700301
H 0.988400 -3.137535 0.789286
H -0.697787 -2.669240 0.804206
C -1.443514 0.117530 1.861944
H -1.587630 0.614183 2.821515
H -1.839231 -0.894561 1.946987
C 0.619973 -1.308885 1.861944
H 1.694328 -1.145540 1.946987
H 0.261917 -1.682020 2.821515
C 0.823541 1.191355 1.861944
H 1.325713 1.067837 2.821515
H 0.144903 2.040101 1.946987
C -1.112170 1.436329 -1.494127
H -1.853488 2.223949 -1.685018
H -0.959180 0.936587 -2.452381
C -1.923122 -0.872479 -1.055125
H -2.440008 -1.408739 -0.254738
H -2.632963 -0.839405 -1.892789

10, neutral form
Stoichiometry: $C_{11}H_{18}N_4$
Charge: 0
Multiplicity: 1
Point group: C_2
DLPNO-CCSD(T), Hartree: -647.7732679
Gibbs in HMPA, Hartree: -647.5337083
Nuclear coordinates, Å:
N 0.469127 -1.486003 -1.022767
C 0.274821 -1.177693 0.313593
C 0.000000 0.000000 0.947568
C -0.274821 1.177693 0.313593
N -0.469127 1.486003 -1.022767
C -1.051681 2.796959 -1.178540
C 1.051681 -2.796959 -1.178540
N -0.458487 2.365127 0.994016
N 0.458487 -2.365127 0.994016
C -0.567008 3.479957 0.091655
C 0.567008 -3.479957 0.091655
H 0.409279 3.965248 -0.069221
H -1.263811 4.234698 0.463163
H -0.699850 3.287906 -2.088595
H -2.151242 2.756455 -1.208810
H 2.151242 -2.756455 -1.208810
H 0.699850 -3.287906 -2.088595
H 1.263811 -4.234698 0.463163
H -0.409279 -3.965248 -0.069221
C 0.000000 2.544168 2.336583
H 0.043260 1.551444 2.787731
H 1.003602 2.991307 2.371794
H -0.686282 3.187987 2.893151
C 0.000000 -2.544168 2.336583
H -0.043260 -1.551444 2.787731
H -1.003602 -2.991307 2.371794
H 0.686282 -3.187987 2.893151
C -0.588374 0.485548 -2.036879
H -1.520593 -0.091195 -1.949393
H -0.610169 1.002875 -2.999736
C 0.588374 -0.485548 -2.036879
H 1.520593 0.091195 -1.949393
H 0.610169 -1.002875 -2.999736

10a, isomer of **10**
Stoichiometry: $C_{11}H_{18}N_4$
Charge: 0
Multiplicity: 1
Point group: C_1
DLPNO-CCSD(T), Hartree: -647.8061108
Gibbs in HMPA, Hartree: -647.5690314
Nuclear coordinates, Å:
N -1.628202 1.265971 -0.402093
C -1.498826 0.005051 -0.161981
C -0.286000 -0.760432 -0.041534
C 1.040102 -0.433836 0.004818
N 1.706360 0.773124 -0.007505
C 3.138037 0.573346 0.089505
C -3.048925 1.541912 -0.533265
N 2.010317 -1.421131 0.096575
N -2.714497 -0.693624 -0.090550
C 3.290912 -0.880000 -0.298587
C -3.744381 0.320148 0.058279
H 3.457531 -0.986327 -1.381226
H 4.107562 -1.378122 0.226181
H 3.663837 1.254206 -0.581156
H 3.481473 0.763514 1.113736
H -3.299979 1.661471 -1.595501
H -3.322342 2.471683 -0.030653
H -4.662698 0.036792 -0.460339
H -3.983432 0.470214 1.123830
C 1.728236 -2.793821 -0.211815
H 0.998436 -3.207293 0.486020
H 1.349950 -2.931220 -1.234241

H 2.648160 -3.367669 -0.100122
C -2.889837 -1.890934 0.687253
H -2.200787 -2.674859 0.375305
H -2.762112 -1.723264 1.767520
H -3.899304 -2.271524 0.522029
H -0.452237 -1.824736 -0.012875
C 1.170536 2.032688 0.186499
H 0.090984 2.067549 0.064336
C 1.899296 3.117631 0.455378
H 2.975381 3.110583 0.571196
H 1.396832 4.068110 0.563666

10, protonated form
Stoichiometry: $C_{11}H_{19}N_4^+$
Charge: +1
Multiplicity: 1
Point group: C_2
DLPNO-CCSD(T), Hartree: -648.2690972
Gibbs in HMPA, Hartree: -648.0612544
Nuclear coordinates, Å:
N 0.177128 -1.548719 -1.052528
C 0.143207 -1.249748 0.266508
C 0.000000 0.000000 0.865485
C -0.143207 1.249748 0.266508
N -0.177128 1.548719 -1.052528
C -0.554579 2.945558 -1.233489
C 0.554579 -2.945558 -1.233489
N -0.270440 2.384481 0.983347
N 0.270440 -2.384481 0.983347
C -0.181514 3.544915 0.110619
C 0.181514 -3.544915 0.110619
H 0.839125 3.945454 0.106612
H -0.864088 4.330241 0.433039
H -0.011957 3.390779 -2.066694
H -1.630450 3.028976 -1.427453
H 1.630450 -3.028976 -1.427453
H 0.011957 -3.390779 -2.066694
H 0.864088 -4.330241 0.433039
H -0.839125 -3.945454 0.106612
C 0.000000 2.499070 2.394352
H -0.597399 1.785043 2.961295
H 1.059280 2.348693 2.626829
H -0.288662 3.497067 2.719095
C 0.000000 -2.499070 2.394352
H 0.597399 -1.785043 2.961295
H -1.059280 -2.348693 2.626829
H 0.288662 -3.497067 2.719095
H 0.000000 0.000000 1.942570
C -0.466303 0.597315 -2.095656
H -1.507092 0.254884 -2.047154
H -0.337661 1.122523 -3.043050
C 0.466303 -0.597315 -2.095656
H 1.507092 -0.254884 -2.047154
H 0.337661 -1.122523 -3.043050

11, neutral form
Stoichiometry: $C_{13}H_{24}N_6$
Charge: 0
Multiplicity: 1
Point group: C_2
DLPNO-CCSD(T), Hartree: -836.7522270
Gibbs in HMPA, Hartree: -836.4428140
Nuclear coordinates, Å:
C -0.225319 0.710885 -0.935676
C 0.225319 -0.710885 -0.935676
C -0.533699 -1.060164 1.246878
C 0.533699 1.060164 1.246878
N 0.879731 -1.188391 -2.049104
N -0.879731 1.188391 -2.049104
C -1.732080 0.317176 -2.830206
H -2.746635 0.263494 -2.414095
H -1.803690 0.700759 -3.849810
H -1.316586 -0.686205 -2.873434
C 1.732080 -0.317176 -2.830206
H 2.746635 -0.263494 -2.414095
H 1.803690 -0.700759 -3.849810
H 1.316586 0.686205 -2.873434
N 1.484338 1.883869 1.787744
N -1.484338 -1.883869 1.787744
C 2.070471 1.547991 3.053583
H 3.070553 1.110361 2.940853
H 2.160508 2.446031 3.673561
H 1.423262 0.816870 3.540145
C -2.070471 -1.547991 3.053583
H -2.160508 -2.446031 3.673561
H -1.423262 -0.816870 3.540145
H -3.070553 -1.110361 2.940853
C 2.007515 3.042733 1.124413
H 1.425954 3.251376 0.230997
H 1.958958 3.915086 1.786606
H 3.058778 2.905525 0.837250
C -2.007515 -3.042733 1.124413
H -1.425954 -3.251376 0.230997
H -1.958958 -3.915086 1.786606
H -3.058778 -2.905525 0.837250
C 0.000000 0.000000 1.919997
N 0.000000 1.511768 0.044030
N 0.000000 -1.511768 0.044030
C -1.309274 2.567868 -2.009106
H -1.642722 2.853589 -3.007441
H -2.129895 2.726260 -1.299121
H -0.484800 3.208549 -1.703501
C 1.309274 -2.567868 -2.009106
H 1.642722 -2.853589 -3.007441
H 2.129895 -2.726260 -1.299121
H 0.484800 -3.208549 -1.703501

11a, tautomer of **11**
Stoichiometry: $C_{13}H_{24}N_6$
Charge: 0
Multiplicity: 1
Point group: C_1
DLPNO-CCSD(T), Hartree: -836.7559577
Gibbs in HMPA, Hartree: -836.4417364
Nuclear coordinates, Å:
C 1.237291 0.431901 -0.380490
C 0.919649 -0.782027 0.390963
C -1.304605 -1.020812 -0.225971
C -0.815480 1.406409 0.127025
N 1.691992 -1.211948 1.383592
N 2.392865 0.473643 -1.083383
C 3.159129 -0.738057 -1.286303
H 2.629205 -1.452369 -1.925126
H 4.098568 -0.473590 -1.770408
H 3.404878 -1.199704 -0.322717
C 2.859622 -0.682864 1.832377
H 3.205783 -1.040018 2.787331
H 3.104664 0.320139 1.518139
N -1.224944 2.613792 0.632748
N -2.291244 -1.834107 -0.733465
C -2.536254 2.750621 1.202123
H -2.586944 3.696483 1.741715
H -3.334049 2.749059 0.445363
H -2.734450 1.944037 1.910253
C -3.632855 -1.367917 -0.947801
H -4.246987 -1.370612 -0.035033
H -3.630627 -0.358699 -1.358267
H -4.119903 -2.024558 -1.671352
C -0.565110 3.848183 0.285202
H 0.433205 3.635175 -0.081127
H -1.114004 4.395316 -0.493330
H -0.494396 4.490800 1.166555
C -2.102857 -3.261334 -0.779128
H -1.038550 -3.477670 -0.793052
H -2.553640 -3.764524 0.087656
H -2.565726 -3.665175 -1.683090
C -1.671282 0.324236 0.126706
N 0.415432 1.430838 -0.465936
N -0.122117 -1.555353 -0.063302
C 2.637321 1.591865 -1.963192
H 3.700378 1.629024 -2.200563
H 2.066722 1.513865 -2.895951
H 2.341702 2.517322 -1.472908
C 1.258536 -2.452404 2.025712
H 2.027668 -2.756726 2.728985
H 0.312873 -2.290164 2.540431
H 1.107284 -3.220898 1.270492
H -2.706160 0.498644 0.370889

11, protonated form
Stoichiometry: $C_{13}H_{25}N_6^+$
Charge: +1
Multiplicity: 1
Point group: C_2
DLPNO-CCSD(T), Hartree: -837.2459968
Gibbs in HMPA, Hartree: -836.9649680
Nuclear coordinates, Å:
C -0.292947 0.687687 -1.048167
C 0.292947 -0.687687 -1.048167
C -0.418986 -1.167275 1.155235
C 0.418986 1.167275 1.155235
N 1.121884 -1.074671 -2.023877
N -1.121884 1.074671 -2.023877
C -1.604484 0.204968 -3.076027

H   -2.694906   0.160872   -3.040049
H   -1.308432   0.580832   -4.058226
H   -1.221568   -0.804368   -2.952803
C   1.604484   -0.204968   -3.076027
H   2.694906   -0.160872   -3.040049
H   1.308432   -0.580832   -4.058226
H   1.221568   0.804368   -2.952803
N   1.131385   2.101062   1.810679
N   -1.131385   -2.101062   1.810679
C   1.623140   1.861987   3.148457
H   2.452202   2.541442   3.340150
H   0.854282   2.040339   3.908104
H   1.988054   0.839885   3.246473
C   -1.623140   -1.861987   3.148457
H   -0.854282   -2.040339   3.908104
H   -1.988054   -0.839885   3.246473
H   -2.452202   -2.541442   3.340150
C   1.344860   3.434378   1.289184
H   0.795508   3.559573   0.362255
H   1.000006   4.174992   2.014961
H   2.407653   3.605808   1.100486
C   -1.344860   -3.434378   1.289184
H   -0.795508   -3.559573   0.362255
H   -1.000006   -4.174992   2.014961
H   -2.407653   -3.605808   1.100486
C   0.000000   0.000000   1.814732
N   0.000000   1.500921   -0.087828
N   0.000000   -1.500921   -0.087828
C   -1.701339   2.405068   -1.951119
H   -2.268077   2.584700   -2.861835
H   -2.363565   2.497817   -1.088093
H   -0.917987   3.157209   -1.860002
C   1.701339   -2.405068   -1.951119
H   2.268077   -2.584700   -2.861835
H   2.363565   -2.497817   -1.088093
H   0.917987   -3.157209   -1.860002
H   0.000000   0.000000   2.892366

11a, transition state
Stoichiometry: $C_{13}H_{24}N_6$
Charge: 0
Multiplicity: 1
Point group: C_1
DLPNO-CCSD(T), Hartree: -836.6841690
Gibbs in HMPA, Hartree: -836.3755924
Nuclear coordinates, Å:
C   -0.607091   -1.096436   -0.276969
C   -0.946555   0.026344   0.611984
C   0.676274   1.518370   -0.232358
C   1.685785   -0.678676   0.200914
N   -2.981181   0.227449   1.050496
N   -1.502665   -1.866156   -0.959735
C   -2.630471   -1.250493   -1.627585
H   -2.385987   -1.035982   -2.676076
H   -3.491171   -1.923811   -1.613157
H   -2.898377   -0.324543   -1.129222
C   -3.696624   -0.736034   1.468053
H   -4.321049   -0.658171   2.361731
H   -3.640201   -1.696214   0.963122
N   2.690111   -1.428112   0.767110
N   0.844733   2.754593   -0.832038
C   3.861449   -0.777458   1.281433
H   4.412594   -1.484489   1.902890
H   4.538896   -0.423767   0.489748
H   3.581905   0.076800   1.900014
C   2.145899   3.368537   -0.859819
H   2.473621   3.748728   0.120148
H   2.892860   2.662960   -1.222536
H   2.121961   4.210526   -1.552890
C   2.840468   -2.834289   0.489386
H   1.917184   -3.219800   0.070326
H   3.656401   -3.017794   -0.223845
H   3.067404   -3.377968   1.410716
C   -0.277005   3.656211   -0.815089
H   -1.180376   3.133324   -1.124263
H   -0.463002   4.082597   0.182090
H   -0.087993   4.473035   -1.512753
C   1.815619   0.684973   0.056037
N   0.646544   -1.418761   -0.306638
N   -0.516588   1.166178   0.138661
C   -0.992227   -3.035561   -1.640022
H   -1.838481   -3.661731   -1.927930
H   -0.421389   -2.777508   -2.540100
H   -0.338898   -3.596917   -0.976019
C   -3.090746   1.501694   1.717499
H   -3.624012   1.429423   2.671895
H   -2.091485   1.903100   1.880338
H   -3.626614   2.196841   1.065627
H   2.770881   1.151930   0.234371

11H^+·**11** complex
Stoichiometry: $C_{26}H_{49}N_{12}^+$
Charge: +1
Multiplicity: 1
Point group: C_1
DLPNO-CCSD(T), Hartree: -1674.0250311
Gibbs in HMPA, Hartree: -1673.4016237
Nuclear coordinates, Å:
C   4.564680   -0.524510   0.957395
C   5.190165   0.101787   -0.244126
C   3.238111   1.305608   -0.735969
C   2.557460   -0.894978   -0.194652
N   6.440520   -0.281149   -0.624228
N   5.255332   -0.549967   2.133716
C   6.298313   0.406457   2.435426
H   5.903715   1.255694   3.005608
H   7.075223   -0.072629   3.034301
H   6.753050   0.783112   1.523411
C   7.012088   -1.556915   -0.252158
H   6.892881   -2.289884   -1.058148
H   8.079927   -1.441328   -0.055209
H   6.538708   -1.946865   0.644472
N   1.864657   -2.045455   -0.527898
N   2.955640   2.640501   -0.776233
C   1.194484   -2.084581   -1.801243
H   1.819278   -2.552147   -2.573639
H   0.267022   -2.661532   -1.726610
H   0.984749   -1.060200   -2.105317
C   1.603860   3.094053   -0.635178
H   1.416009   3.923969   -1.323182
H   0.944926   2.257388   -0.865308
H   1.388875   3.443342   0.383040
C   2.264595   -3.332371   -0.014637
H   2.599809   -3.239405   1.014742
H   1.411107   -4.014233   -0.050911
H   3.073910   -3.785117   -0.604392
C   3.963285   3.665039   -0.850830
H   4.923377   3.220532   -1.093618
H   3.702065   4.390121   -1.628009
H   4.055359   4.209976   0.096695
C   2.262961   0.328837   -0.736680
N   3.393037   -1.054673   0.895374
N   4.574752   1.009276   -0.919025
C   4.607819   -1.140915   3.282931
H   5.357994   -1.305371   4.056250
H   3.818832   -0.495909   3.686365
H   4.154079   -2.090910   3.008966
C   7.002307   0.326760   -1.811014
H   8.068908   0.105189   -1.840255
H   6.534117   -0.053496   -2.725233
H   6.855088   1.404214   -1.786345
C   -2.858708   -0.467776   0.072043
C   -3.196486   0.874598   -0.494171
C   -5.220709   1.020366   0.694098
C   -5.042336   -1.295816   -0.201279
N   -2.505991   1.326897   -1.542123
N   -1.714243   -0.629756   0.725280
C   -0.768499   0.443374   0.974889
H   -0.503126   0.422358   2.033800
H   0.150631   0.324583   0.382429
H   -1.220848   1.409236   0.761907
C   -1.451852   0.563991   -2.183849
H   -1.539080   0.703011   -3.262072
H   -0.451808   0.879715   -1.870259
H   -1.563729   -0.496850   -1.972235
N   -5.706904   -2.318312   -0.778497
N   -5.854134   1.840870   1.558098
C   -7.143305   -2.291570   -0.922446
H   -7.427495   -3.000773   -1.698641
H   -7.658999   -2.576016   0.001806
H   -7.481419   -1.300557   -1.224702
C   -7.019931   1.399292   2.286012
H   -7.928970   1.432965   1.674657
H   -6.880882   0.383113   2.655217
H   -7.164717   2.057036   3.141811
C   -5.059594   -3.555512   -1.154966
H   -4.003205   -3.505677   -0.913832
H   -5.512290   -4.395705   -0.620671
H   -5.170974   -3.730759   -2.228025
C   -5.521169   3.241941   1.691964
H   -4.681666   3.484002   1.049249
H   -6.377654   3.863555   1.414661
H   -5.253176   3.468633   2.726829
C   -5.759667   -0.241169   0.395376

N   -3.705378   -1.437816   -0.101933
N   -4.162097   1.551406   0.041319
C   -1.347708   -1.915317   1.285954
H   -0.329844   -2.154258   0.969997
H   -1.376153   -1.867695   2.377349
H   -2.041297   -2.676301   0.942305
C   -2.806407   2.615683   -2.131146
H   -1.916299   3.248603   -2.107003
H   -3.111439   2.485977   -3.171813
H   -3.608683   3.091297   -1.575905
H   -6.794767   -0.414887   0.638070

11H$^+$·**11** complex, transition state
Stoichiometry: $C_{26}H_{49}N_{12}^+$
Charge: +1
Multiplicity: 1
Point group: C_1
DLPNO-CCSD(T), Hartree: -1673.9788304
Gibbs in HMPA, Hartree: -1673.3509503
Nuclear coordinates, Å:
C   4.781799   -0.050123   -0.172435
C   3.899601   -0.674813   0.851238
C   2.062225   -0.670318   -0.620866
C   3.091694   1.462678   -0.892368
N   4.334956   -0.843348   2.122574
N   6.021371   -0.556851   -0.394314
C   6.399968   -1.901060   -0.017947
H   6.399752   -2.560678   -0.892277
H   7.406080   -1.902275   0.406848
H   5.713397   -2.308593   0.718817
C   5.485090   -0.154432   2.666563
H   5.167728   0.645730   3.342999
H   6.107951   -0.850932   3.232228
H   6.086068   0.281972   1.874288
N   3.056250   2.824037   -1.017506
N   1.291440   -1.630869   -1.190149
C   1.955350   3.489096   -1.668181
H   1.312300   4.028948   -0.964816
H   2.345747   4.216275   -2.385574
H   1.361022   2.755505   -2.206819
C   0.635907   -1.398839   -2.454251
H   -0.450761   -1.482684   -2.355981
H   0.894527   -0.403196   -2.806932
H   0.970235   -2.139446   -3.187480
C   4.252544   3.619776   -0.876150
H   4.916041   3.194042   -0.128464
H   4.805973   3.705219   -1.819635
H   3.970532   4.623188   -0.552376
C   1.227826   -2.985950   -0.702558
H   1.612479   -3.035142   0.311648
H   0.190851   -3.331829   -0.713417
H   1.814175   -3.662580   -1.334288
C   1.968013   0.666827   -1.015178
N   4.380475   0.969406   -0.846433
N   2.714670   -1.059569   0.517177
C   6.842442   0.056035   -1.417564
H   7.878399   -0.239246   -1.252114
H   6.539978   -0.259387   -2.421542
H   6.755632   1.138444   -1.365789
C   3.436541   -1.426337   3.093631
H   4.022900   -1.780068   3.941194
H   2.700812   -0.699549   3.454906
H   2.899183   -2.262169   2.650673
C   -2.488751   -0.308411   0.785912
C   -2.828573   1.065638   0.293778
C   -5.159158   0.730217   0.357228
C   -4.120272   -1.333977   -0.569608
N   -1.911447   1.789987   -0.267399
N   -1.665969   -0.450580   1.837761
C   -1.031073   0.650774   2.523892
H   -1.243375   0.578339   3.593259
H   0.053974   0.618673   2.385220
H   -1.402943   1.602651   2.158963
C   -0.128579   1.261248   -0.654841
H   0.302144   2.014971   -0.022038
H   -0.243823   0.255308   -0.294216
H   -0.259516   1.460337   -1.701653
N   -4.187519   -2.329133   -1.493874
N   -6.315535   1.246269   0.835113
C   -5.331647   -2.450403   -2.361812
H   -5.067356   -3.096332   -3.198443
H   -6.199570   -2.890775   -1.855208
H   -5.616694   -1.476111   -2.759810
C   -7.560041   0.518704   0.775059
H   -8.068237   0.636617   -0.189237
H   -7.397185   -0.542481   0.959979
H   -8.223308   0.899481   1.551532
C   -3.292867   -3.462695   -1.482246
H   -2.507460   -3.299547   -0.751911
H   -3.833184   -4.380375   -1.224664
H   -2.842495   -3.599625   -2.469047
C   -6.395111   2.593272   1.352030
H   -5.459731   3.109515   1.160273
H   -7.214165   3.129588   0.865433
H   -6.580674   2.588187   2.430253
C   -5.205051   -0.484652   -0.377543
N   -3.006237   -1.343091   0.207357
N   -4.068697   1.476524   0.514263
C   -1.371957   -1.760764   2.374741
H   -0.325381   -2.021215   2.190165
H   -1.543926   -1.760752   3.453441
H   -2.015638   -2.497946   1.904792
C   -2.284385   3.085338   -0.781922
H   -1.426079   3.522767   -1.293786
H   -3.118849   3.019345   -1.484505
H   -2.590571   3.759742   0.023328
H   -6.152467   -0.772359   -0.801837

11b, dimer of **11**
Stoichiometry: $C_{26}H_{48}N_{12}$
Charge: 0
Multiplicity: 3
Point group: C_2
DLPNO-CCSD(T), Hartree: -1673.5720104
Gibbs in gas, Hartree: -1672.9039174
Nuclear coordinates, Å:
C   -0.651132   3.580115   -0.380214
C   0.706368   3.736460   0.109029
C   1.135299   1.459000   -0.154878
C   -1.136633   1.515349   0.702285
N   1.216121   4.993400   0.378745
N   -1.257434   4.589365   -1.139567
C   -0.513703   5.059833   -2.287279
H   -0.535088   4.339229   -3.119681
H   -0.945501   5.999802   -2.637877
H   0.523625   5.243294   -2.013116
C   0.371570   5.989058   1.005664
H   0.341098   5.862232   2.096556
H   0.767240   6.984529   0.791993
H   -0.638881   5.926408   0.610248
N   -2.123938   0.927963   1.483422
N   2.118805   0.958126   -0.962200
C   -1.783030   0.142381   2.645930
H   -1.549486   0.775850   3.515359
H   -2.630958   -0.492789   2.909361
H   -0.939882   -0.508324   2.441821
C   1.904943   -0.049650   -1.968389
H   2.654265   -0.842016   -1.883321
H   0.927625   -0.507073   -1.864301
H   1.989610   0.393968   -2.969372
C   -3.366278   1.644951   1.630365
H   -3.732504   1.973220   0.659562
H   -4.097438   0.971553   2.081916
H   -3.273601   2.536408   2.266369
C   3.411190   1.590649   -1.043010
H   3.643706   2.092573   -0.108515
H   4.163508   0.821380   -1.238678
H   3.465918   2.327985   -1.855470
C   0.023643   0.738383   0.277662
N   -1.449857   2.700020   0.234361
N   1.449857   2.704190   0.353665
C   -2.670256   4.450538   -1.391874
H   -3.035102   5.373812   -1.845756
H   -2.899451   3.617311   -2.074531
H   -3.201723   4.273448   -0.459603
C   2.598709   5.067791   0.786664
H   2.924058   6.108246   0.722235
H   2.754801   4.715964   1.814729
H   3.213832   4.457005   0.129353
C   -0.023643   -0.738383   0.277662
C   -1.135299   -1.459000   -0.154878
C   1.136633   -1.515349   0.702285
N   -2.118805   -0.958126   -0.962200
N   -1.449857   -2.704190   0.353665
N   2.123938   -0.927963   1.483422
N   1.449857   -2.700020   0.234361
C   -1.904943   0.049650   -1.968389
C   -3.411190   -1.590649   -1.043010
C   -0.706368   -3.736460   0.109029
C   1.783030   -0.142381   2.645930
C   3.366278   -1.644951   1.630365
C   0.651132   -3.580115   -0.380214
H   -2.654265   0.842016   -1.883321
H   -0.927625   0.507073   -1.864301

H	-1.989610	-0.393968	-2.969372
H	-3.643706	-2.092573	-0.108515
H	-4.163508	-0.821380	-1.238678
H	-3.465918	-2.327985	-1.855470
N	-1.216121	-4.993400	0.378745
H	1.549486	-0.775850	3.515359
H	2.630958	0.492789	2.909361
H	0.939882	0.508324	2.441821
H	3.732504	-1.973220	0.659562
H	4.097438	-0.971553	2.081916
H	3.273601	-2.536408	2.266369
N	1.257434	-4.589365	-1.139567
C	-0.371570	-5.989058	1.005664
C	-2.598709	-5.067791	0.786664
C	0.513703	-5.059833	-2.287279
C	2.670256	-4.450538	-1.391874
H	-0.341098	-5.862232	2.096556
H	-0.767240	-6.984529	0.791993
H	0.638881	-5.926408	0.610248
H	-2.924058	-6.108246	0.722235
H	-2.754801	-4.715964	1.814729
H	-3.213832	-4.457005	0.129353
H	0.535088	-4.339229	-3.119681
H	0.945501	-5.999802	-2.637877
H	-0.523625	-5.243294	-2.013116
H	3.035102	-5.373812	-1.845756
H	2.899451	-3.617311	-2.074531
H	3.201723	-4.273448	-0.459603

11b^{2+}, dication of **11b**
Stoichiometry: $C_{26}H_{48}N_{12}^{2+}$
Charge: +2
Multiplicity: 1
Point group: D_2
DLPNO-CCSD(T), Hartree: -1673.2234310
Gibbs in gas, Hartree: -1672.5485520
Nuclear coordinates, Å:

C	-0.363754	0.652578	-3.690714
C	0.363754	-0.652578	-3.690714
C	-0.420976	-1.139130	-1.498321
C	0.420976	1.139130	-1.498321
N	1.204574	-0.981727	-4.676892
N	-1.204574	0.981727	-4.676892
C	-1.619731	0.069565	-5.724897
H	-2.701898	-0.071276	-5.677553
H	-1.371786	0.476318	-6.707489
H	-1.144503	-0.900665	-5.611517
C	1.619731	-0.069565	-5.724897
H	2.701898	0.071276	-5.677553
H	1.371786	-0.476318	-6.707489
H	1.144503	0.900665	-5.611517
N	1.229849	2.112118	-1.033657
N	-1.229849	-2.112118	-1.033657
C	2.212588	1.942727	0.015764
H	3.205179	2.149188	-0.393288
H	2.028556	2.640764	0.835128
H	2.197580	0.935797	0.412590
C	-2.212588	-1.942727	0.015764
H	-2.028556	-2.640764	0.835128
H	-2.197580	-0.935797	0.412590
H	-3.205179	-2.149188	-0.393288
C	1.356631	3.373038	-1.743529
H	0.404672	3.663134	-2.179299
H	1.675923	4.134767	-1.032362
H	2.101574	3.315874	-2.542235
C	-1.356631	-3.373038	-1.743529
H	-0.404672	-3.663134	-2.179299
H	-1.675923	-4.134767	-1.032362
H	-2.101574	-3.315874	-2.542235
C	0.000000	0.000000	-0.739614
N	-0.156566	1.434605	-2.686491
N	0.156566	-1.434605	-2.686491
C	-1.912238	2.247516	-4.623111
H	-2.177135	2.541223	-5.637758
H	-2.828723	2.164738	-4.032291
H	-1.276766	3.011042	-4.181462
C	1.912238	-2.247516	-4.623111
H	2.177135	-2.541223	-5.637758
H	2.828723	-2.164738	-4.032291
H	1.276766	-3.011042	-4.181462
C	0.000000	0.000000	0.739614
C	-0.420976	1.139130	1.498321
C	0.420976	-1.139130	1.498321
N	-1.229849	2.112118	1.033657
N	0.156566	1.434605	2.686491
N	1.229849	-2.112118	1.033657
N	-0.156566	-1.434605	2.686491
C	-2.212588	1.942727	-0.015764
C	-1.356631	3.373038	1.743529
C	0.363754	0.652578	3.690714
C	2.212588	-1.942727	-0.015764
C	1.356631	-3.373038	1.743529
C	-0.363754	-0.652578	3.690714
H	-2.028556	2.640764	-0.835128
H	-2.197580	0.935797	-0.412590
H	-3.205179	2.149188	0.393288
H	-0.404672	3.663134	2.179299
H	-1.675923	4.134767	1.032362
H	-2.101574	3.315874	2.542235
N	1.204574	0.981727	4.676892
H	3.205179	-2.149188	0.393288
H	2.028556	-2.640764	-0.835128
H	2.197580	-0.935797	-0.412590
H	0.404672	-3.663134	2.179299
H	1.675923	-4.134767	1.032362
H	2.101574	-3.315874	2.542235
N	-1.204574	-0.981727	4.676892
C	1.619731	0.069565	5.724897
C	1.912238	2.247516	4.623111
C	-1.619731	-0.069565	5.724897
C	-1.912238	-2.247516	4.623111
H	2.701898	-0.071276	5.677553
H	1.371786	0.476318	6.707489
H	1.144503	-0.900665	5.611517
H	2.177135	2.541223	5.637758
H	2.828723	2.164738	4.032291
H	1.276766	3.011042	4.181462
H	-2.701898	0.071276	5.677553
H	-1.371786	-0.476318	6.707489
H	-1.144503	0.900665	5.611517
H	-2.177135	-2.541223	5.637758
H	-2.828723	-2.164738	4.032291
H	-1.276766	-3.011042	4.181462

1, dimer
Stoichiometry: $C_{46}H_{52}N_8$
Charge: 0
Multiplicity: 1
Point group: D_2
DLPNO-CCSD(T), Hartree: -2218.1465616
Gibbs in HMPA, Hartree: -2217.3374812
Nuclear coordinates, Å:

C	0.000000	0.000000	-0.665853
C	1.260116	0.031862	-1.493975
C	-1.260116	-0.031862	-1.493975
C	2.873173	-1.347993	-2.765685
C	1.433646	-2.427929	-1.146094
C	2.792810	-2.839493	-3.053909
H	3.875774	-1.062463	-2.432236
H	2.632299	-0.727549	-3.628828
C	2.441504	-3.424949	-1.693461
H	0.424329	-2.641068	-1.507012
H	1.419904	-2.390186	-0.057801
H	1.989700	-3.040850	-3.767545
H	3.721850	-3.226643	-3.472597
H	2.029133	-4.432343	-1.747543
H	3.327969	-3.457967	-1.054474
C	-2.873173	1.347993	-2.765685
C	-1.433646	2.427929	-1.146094
C	-2.792810	2.839493	-3.053909
H	-3.875774	1.062463	-2.432236
H	-2.632299	0.727549	-3.628828
C	-2.441504	3.424949	-1.693461
H	-0.424329	2.641068	-1.507012
H	-1.419904	2.390186	-0.057801
H	-1.989700	3.040850	-3.767545
H	-3.721850	3.226643	-3.472597
H	-2.029133	4.432343	-1.747543
H	-3.327969	3.457967	-1.054474
N	1.889774	-1.143323	-1.702569
N	-1.889774	1.143323	-1.702569
N	1.543502	1.193692	-1.970792
N	-1.543502	-1.193692	-1.970792
C	2.736643	1.602013	-2.547739
C	3.960374	1.493410	-1.875083
C	2.716864	2.279878	-3.771558
C	5.119598	2.020364	-2.422304
H	3.984672	1.006056	-0.906808
C	3.880855	2.791945	-4.317358
H	1.769589	2.390715	-4.285729
C	5.092323	2.666185	-3.649561
H	6.054168	1.925276	-1.880807
H	3.840816	3.301866	-5.273235
H	5.999973	3.075170	-4.075553

```
C   -2.736643   -1.602013   -2.547739
C   -3.960374   -1.493410   -1.875083
C   -2.716864   -2.279878   -3.771558
C   -5.119598   -2.020364   -2.422304
H   -3.984672   -1.006056   -0.906808
C   -3.880855   -2.791945   -4.317358
H   -1.769589   -2.390715   -4.285729
C   -5.092323   -2.666185   -3.649561
H   -6.054168   -1.925276   -1.880807
H   -3.840816   -3.301866   -5.273235
H   -5.999973   -3.075170   -4.075553
C    0.000000    0.000000    0.665853
C   -1.260116    0.031862    1.493975
C    1.260116   -0.031862    1.493975
N   -1.889774   -1.143323    1.702569
N   -1.543502    1.193692    1.970792
N    1.889774    1.143323    1.702569
N    1.543502   -1.193692    1.970792
C   -2.873173   -1.347993    2.765685
C   -1.433646   -2.427929    1.146094
C   -2.736643    1.602013    2.547739
C    2.873173    1.347993    2.765685
C    1.433646    2.427929    1.146094
C    2.736643   -1.602013    2.547739
C   -2.792810   -2.839493    3.053909
H   -3.875774   -1.062463    2.432236
H   -2.632299   -0.727549    3.628828
C   -2.441504   -3.424949    1.693461
H   -0.424329   -2.641068    1.507012
H   -1.419904   -2.390186    0.057801
C   -3.960374    1.493410    1.875083
C   -2.716864    2.279878    3.771558
C    2.792810    2.839493    3.053909
H    3.875774    1.062463    2.432236
H    2.632299    0.727549    3.628828
C    2.441504    3.424949    1.693461
H    0.424329    2.641068    1.507012
H    1.419904    2.390186    0.057801
C    3.960374   -1.493410    1.875083
C    2.716864   -2.279878    3.771558
H   -1.989700   -3.040850    3.767545
H   -3.721850   -3.226643    3.472597
H   -2.029133   -4.432343    1.747543
H   -3.327969   -3.457967    1.054474
C   -5.119598    2.020364    2.422304
H   -3.984672    1.006056    0.906808
C   -3.880855    2.791945    4.317358
H   -1.769589    2.390715    4.285729
H    1.989700    3.040850    3.767545
H    3.721850    3.226643    3.472597
H    2.029133    4.432343    1.747543
H    3.327969    3.457967    1.054474
C    5.119598   -2.020364    2.422304
H    3.984672   -1.006056    0.906808
C    3.880855   -2.791945    4.317358
H    1.769589   -2.390715    4.285729
C   -5.092323    2.666185    3.649561
H   -6.054168    1.925276    1.880807
H   -3.840816    3.301866    5.273235
C    5.092323   -2.666185    3.649561
H    6.054168   -1.925276    1.880807
H    3.840816   -3.301866    5.273235
H   -5.999973    3.075170    4.075553
H    5.999973   -3.075170    4.075553
```

Schwesinger’s $[P(N{=}P(NMe_2)_3)_4]^+$ cation
Stoichiometry: $C_{24}H_{72}N_{16}P_5^+$
Charge: +1
Multiplicity: 1
Point group: S_4
DLPNO-CCSD(T), Hartree: -3535.3857610
Gibbs in HMPA, Hartree: -3534.4893586
Gibbs in gas, Hartree: -3534.4484750
Nuclear coordinates, Å:

```
P    0.000000    0.000000    0.000000
N   -0.974608    0.896439    0.946313
P   -1.122010    2.341722    1.526527
N    0.000000    2.687246    2.712498
N   -2.687024    2.466399    2.130723
N   -1.040602    3.668209    0.525147
C    0.860912    1.678785    3.284657
C   -0.147271    3.869025    3.538489
C   -3.159017    1.395940    2.988040
C   -3.305736    3.738075    2.456107
C   -1.988386    3.759259   -0.569524
C    0.198098    4.381947    0.279776
H    0.496978    1.347079    4.265677
H    0.920996    0.818140    2.622464
H    1.866922    2.088575    3.426768
H   -0.743631    4.626511    3.032923
H   -0.620189    3.638337    4.500750
H    0.836452    4.301247    3.746661
H   -2.884607    1.557266    4.039227
H   -4.249775    1.339279    2.935512
H   -2.748388    0.445601    2.657258
H   -3.174209    4.004875    3.511995
H   -2.894667    4.536921    1.844269
H   -4.380818    3.674878    2.263147
H   -1.560574    3.378696   -1.502473
H   -2.884950    3.183792   -0.344858
H   -2.276911    4.803158   -0.725259
H   -0.032191    5.429741    0.065594
H    0.839111    4.343878    1.156410
H    0.751679    3.961142   -0.563318
N   -0.896439   -0.974608   -0.946313
P   -2.341722   -1.122010   -1.526527
N   -2.466399   -2.687024   -2.130723
N   -2.687246    0.000000   -2.712498
N   -3.668209   -1.040602   -0.525147
C   -1.395940   -3.159017   -2.988040
C   -3.738075   -3.305736   -2.456107
C   -1.678785    0.860912   -3.284657
C   -3.869025   -0.147271   -3.538489
C   -4.381947    0.198098   -0.279776
C   -3.759259   -1.988386    0.569524
H   -1.557266   -2.884607   -4.039227
H   -0.445601   -2.748388   -2.657258
H   -1.339279   -4.249775   -2.935512
H   -4.536921   -2.894667   -1.844269
H   -4.004875   -3.174209   -3.511995
H   -3.674878   -4.380818   -2.263147
H   -1.347079    0.496978   -4.265677
H   -2.088575    1.866922   -3.426768
H   -0.818140    0.920996   -2.622464
H   -3.638337   -0.620189   -4.500750
H   -4.626511   -0.743631   -3.032923
H   -4.301247    0.836452   -3.746661
H   -3.961142    0.751679    0.563318
H   -4.343878    0.839111   -1.156410
H   -5.429741   -0.032191   -0.065594
H   -4.803158   -2.276911    0.725259
H   -3.183792   -2.884950    0.344858
H   -3.378696   -1.560574    1.502473
N    0.896439    0.974608   -0.946313
P    2.341722    1.122010   -1.526527
N    2.466399    2.687024   -2.130723
N    2.687246    0.000000   -2.712498
N    3.668209    1.040602   -0.525147
C    1.395940    3.159017   -2.988040
C    3.738075    3.305736   -2.456107
C    1.678785   -0.860912   -3.284657
C    3.869025    0.147271   -3.538489
C    4.381947   -0.198098   -0.279776
C    3.759259    1.988386    0.569524
H    1.557266    2.884607   -4.039227
H    0.445601    2.748388   -2.657258
H    1.339279    4.249775   -2.935512
H    4.536921    2.894667   -1.844269
H    4.004875    3.174209   -3.511995
H    3.674878    4.380818   -2.263147
H    1.347079   -0.496978   -4.265677
H    2.088575   -1.866922   -3.426768
H    0.818140   -0.920996   -2.622464
H    3.638337    0.620189   -4.500750
H    4.626511    0.743631   -3.032923
H    4.301247   -0.836452   -3.746661
H    3.961142   -0.751679    0.563318
H    4.343878   -0.839111   -1.156410
H    5.429741    0.032191   -0.065594
H    4.803158    2.276911    0.725259
H    3.183792    2.884950    0.344858
H    3.378696    1.560574    1.502473
N    0.974608   -0.896439    0.946313
P    1.122010   -2.341722    1.526527
N    0.000000   -2.687246    2.712498
N    2.687024   -2.466399    2.130723
N    1.040602   -3.668209    0.525147
C   -0.860912   -1.678785    3.284657
C    0.147271   -3.869025    3.538489
C    3.159017   -1.395940    2.988040
C    3.305736   -3.738075    2.456107
C    1.988386   -3.759259   -0.569524
C   -0.198098   -4.381947    0.279776
H   -0.496978   -1.347079    4.265677
```

H	-0.920996	-0.818140	2.622464
H	-1.866922	-2.088575	3.426768
H	0.743631	-4.626511	3.032923
H	0.620189	-3.638337	4.500750
H	-0.836452	-4.301247	3.746661
H	2.884607	-1.557266	4.039227
H	4.249775	-1.339279	2.935512
H	2.748388	-0.445601	2.657258
H	3.174209	-4.004875	3.511995
H	2.894667	-4.536921	1.844269
H	4.380818	-3.674878	2.263147
H	1.560574	-3.378696	-1.502473
H	2.884950	-3.183792	-0.344858
H	2.276911	-4.803158	-0.725259
H	0.032191	-5.429741	0.065594
H	-0.839111	-4.343878	1.156410
H	-0.751679	-3.961142	-0.563318

[P(N=P(NMe$_2$)$_3$)$_4$]$^+$F$^-$ ion pair

Stoichiometry: $C_{24}H_{72}N_{16}FP_5$

Charge: 0

Multiplicity: 1

Point group: C_2

DLPNO-CCSD(T), Hartree: -3635.2559897

Gibbs in HMPA, Hartree: -3634.3435950

Gibbs in gas, Hartree: -3634.3165537

Nuclear coordinates, Å:

P	0.000000	0.000000	0.199270
N	-0.864681	1.061174	-0.646372
P	-2.144552	1.235348	-1.536640
N	-3.052981	-0.084133	-1.986475
N	-1.831752	1.983909	-2.969789
N	-3.197108	2.236470	-0.686952
C	-2.434630	-1.063371	-2.866008
C	-4.111654	-0.600663	-1.155157
C	-0.593547	2.703491	-3.187696
C	-2.516777	1.703355	-4.220376
C	-4.283768	2.899660	-1.363733
C	-3.312397	2.201893	0.746525
H	-3.166985	-1.400817	-3.608413
H	-1.560822	-0.642082	-3.392463
H	-2.105126	-1.935184	-2.292771
H	-4.613137	0.203609	-0.619114
H	-4.854152	-1.097698	-1.787987
H	-3.749379	-1.329076	-0.418496
H	-0.804744	3.724643	-3.529154
H	-0.023094	2.745004	-2.263772
H	-0.012061	2.155641	-3.935462
H	-1.857119	1.093550	-4.848748
H	-3.452247	1.177886	-4.040707
H	-2.744806	2.649980	-4.724954
H	-4.419011	3.905448	-0.950725
H	-4.059976	2.998881	-2.424247
H	-5.239730	2.366220	-1.257770
H	-4.212244	1.666207	1.084140
H	-2.436546	1.714022	1.169198
H	-3.374454	3.222284	1.144347
N	1.002684	0.865825	1.173947
P	1.131814	2.321157	1.716966
N	2.709101	2.457435	2.284618
N	-0.008077	2.669146	2.900628
N	1.016652	3.689496	0.769381
C	3.446135	1.287640	2.708457
C	3.207029	3.680252	2.872686
C	-0.760633	1.626296	3.558949
C	0.000000	3.913489	3.636998
C	-0.253355	4.312549	0.451741
C	2.067542	4.059270	-0.155712
H	3.380222	1.130286	3.794153
H	3.059891	0.410879	2.197795
H	4.504180	1.410424	2.453740
H	2.681498	4.545047	2.473149
H	3.111520	3.688254	3.966736
H	4.269815	3.792944	2.634209
H	-0.317840	1.346385	4.524724
H	-1.778388	1.982843	3.753719
H	-0.821899	0.744522	2.925209
H	0.484403	3.809266	4.616672
H	0.513115	4.693432	3.078835
H	-1.029063	4.246470	3.811414
H	-0.584985	4.052074	-0.555968
H	-1.020428	3.989687	1.148876
H	-0.151889	5.402178	0.515767
H	2.263000	5.135637	-0.086113
H	2.986497	3.524952	0.072741
H	1.785834	3.836627	-1.189194
N	-1.002684	-0.865825	1.173947
P	-1.131814	-2.321157	1.716966
N	-2.709101	-2.457435	2.284618
N	0.008077	-2.669146	2.900628
N	-1.016652	-3.689496	0.769381
C	-3.446135	-1.287640	2.708457
C	-3.207029	-3.680252	2.872686
C	0.760633	-1.626296	3.558949
C	0.000000	-3.913489	3.636998
C	0.253355	-4.312549	0.451741
C	-2.067542	-4.059270	-0.155712
H	-3.380222	-1.130286	3.794153
H	-3.059891	-0.410879	2.197795
H	-4.504180	-1.410424	2.453740
H	-2.681498	-4.545047	2.473149
H	-3.111520	-3.688254	3.966736
H	-4.269815	-3.792944	2.634209
H	0.317840	-1.346385	4.524724
H	1.778388	-1.982843	3.753719
H	0.821899	-0.744522	2.925209
H	-0.484403	-3.809266	4.616672
H	-0.513115	-4.693432	3.078835
H	1.029063	-4.246470	3.811414
H	0.584985	-4.052074	-0.555968
H	1.020428	-3.989687	1.148876
H	0.151889	-5.402178	0.515767
H	-2.263000	-5.135637	-0.086113
H	-2.986497	-3.524952	0.072741
H	-1.785834	-3.836627	-1.189194
N	0.864681	-1.061174	-0.646372
P	2.144552	-1.235348	-1.536640
N	3.052981	0.084133	-1.986475
N	1.831752	-1.983909	-2.969789
N	3.197108	-2.236470	-0.686952
C	2.434630	1.063371	-2.866008
C	4.111654	0.600663	-1.155157
C	0.593547	-2.703491	-3.187696
C	2.516777	-1.703355	-4.220376
C	4.283768	-2.899660	-1.363733
C	3.312397	-2.201893	0.746525
H	3.166985	1.400817	-3.608413
H	1.560822	0.642082	-3.392463
H	2.105126	1.935184	-2.292771
H	4.613137	-0.203609	-0.619114
H	4.854152	1.097698	-1.787987
H	3.749379	1.329076	-0.418496
H	0.804744	-3.724643	-3.529154
H	0.023094	-2.745004	-2.263772
H	0.012061	-2.155641	-3.935462
H	1.857119	-1.093550	-4.848748
H	3.452247	-1.177886	-4.040707
H	2.744806	-2.649980	-4.724954
H	4.419011	-3.905448	-0.950725
H	4.059976	-2.998881	-2.424247
H	5.239730	-2.366220	-1.257770
H	4.212244	-1.666207	1.084140
H	2.436546	-1.714022	1.169198
H	3.374454	-3.222284	1.144347
F	0.000000	0.000000	-4.371497

12$^+$ cation

Stoichiometry: $C_{40}H_{56}N_4O_8P^+$

Charge: +1

Multiplicity: 1

Point group: S_4

DLPNO-CCSD(T), Hartree: -2715.3569907

Gibbs in HMPA, Hartree: -2714.5791210

Gibbs in gas, Hartree: -2714.5326747

Nuclear coordinates, Å:

P	0.000000	0.000000	0.000000
C	0.830899	-1.145531	1.132637
C	2.215274	-1.276912	1.333941
C	0.000000	-1.891354	1.987209
C	2.729419	-2.150643	2.282070
C	0.494530	-2.763090	2.939883
C	1.880941	-2.917940	3.093027
H	3.796396	-2.221539	2.406874
H	-0.188394	-3.311206	3.566808
C	1.145531	0.830899	-1.132637
C	1.891354	0.000000	-1.987209
C	1.276912	2.215274	-1.333941
C	2.763090	0.494530	-2.939883
C	2.150643	2.729419	-2.282070
C	2.917940	1.880941	-3.093027
H	3.311206	-0.188394	-3.566808
H	2.221539	3.796396	-2.406874
C	-0.830899	1.145531	1.132637
C	-2.215274	1.276912	1.333941

C	0.000000	1.891354	1.987209
C	-2.729419	2.150643	2.282070
C	-0.494530	2.763090	2.939883
C	-1.880941	2.917940	3.093027
H	-3.796396	2.221539	2.406874
H	0.188394	3.311206	3.566808
C	-1.145531	-0.830899	-1.132637
C	-1.891354	0.000000	-1.987209
C	-1.276912	-2.215274	-1.333941
C	-2.763090	-0.494530	-2.939883
C	-2.150643	-2.729419	-2.282070
C	-2.917940	-1.880941	-3.093027
H	-3.311206	0.188394	-3.566808
H	-2.221539	-3.796396	-2.406874
O	-1.319813	-1.696342	1.810218
O	1.696342	-1.319813	-1.810218
O	1.319813	1.696342	1.810218
O	-1.696342	1.319813	-1.810218
O	-3.026052	0.505187	0.588528
O	3.026052	-0.505187	0.588528
O	0.505187	3.026052	-0.588528
O	-0.505187	-3.026052	-0.588528
C	-4.420160	0.641178	0.713894
H	-4.765304	0.372479	1.717381
H	-4.749988	1.658745	0.482601
H	-4.850636	-0.048588	-0.009531
C	4.420160	-0.641178	0.713894
H	4.765304	-0.372479	1.717381
H	4.749988	-1.658745	0.482601
H	4.850636	0.048588	-0.009531
C	0.641178	4.420160	-0.713894
H	1.658745	4.749988	-0.482601
H	0.372479	4.765304	-1.717381
H	-0.048588	4.850636	0.009531
C	-0.641178	-4.420160	-0.713894
H	-1.658745	-4.749988	-0.482601
H	-0.372479	-4.765304	-1.717381
H	0.048588	-4.850636	0.009531
C	2.239572	2.405868	2.602479
H	2.124973	2.164586	3.663278
H	3.225607	2.092480	2.266661
H	2.138297	3.486379	2.462485
C	-2.239572	-2.405868	2.602479
H	-2.124973	-2.164586	3.663278
H	-3.225607	-2.092480	2.266661
H	-2.138297	-3.486379	2.462485
C	2.405868	-2.239572	-2.602479
H	2.092480	-3.225607	-2.266661
H	2.164586	-2.124973	-3.663278
H	3.486379	-2.138297	-2.462485
C	-2.405868	2.239572	-2.602479
H	-2.092480	3.225607	-2.266661
H	-2.164586	2.124973	-3.663278
H	-3.486379	2.138297	-2.462485
N	-2.389342	3.783372	4.015259
N	2.389342	-3.783372	4.015259
N	3.783372	2.389342	-4.015259
N	-3.783372	-2.389342	-4.015259
C	-1.505085	4.504361	4.896020
H	-0.809558	5.139245	4.337717
H	-2.093607	5.149771	5.543604
H	-0.917284	3.832974	5.532785
C	1.505085	-4.504361	4.896020
H	2.093607	-5.149771	5.543604
H	0.917284	-3.832974	5.532785
H	0.809558	-5.139245	4.337717
C	-4.504361	-1.505085	-4.896020
H	-3.832974	-0.917284	-5.532785
H	-5.149771	-2.093607	-5.543604
H	-5.139245	-0.809558	-4.337717
C	4.504361	1.505085	-4.896020
H	3.832974	0.917284	-5.532785
H	5.149771	2.093607	-5.543604
H	5.139245	0.809558	-4.337717
C	-3.814331	3.887515	4.203976
H	-4.321427	4.182152	3.279814
H	-4.256926	2.946072	4.550548
H	-4.021235	4.649729	4.951337
C	3.814331	-3.887515	4.203976
H	4.321427	-4.182152	3.279814
H	4.256926	-2.946072	4.550548
H	4.021235	-4.649729	4.951337
C	-3.887515	-3.814331	-4.203976
H	-2.946072	-4.256926	-4.550548
H	-4.182152	-4.321427	-3.279814
H	-4.649729	-4.021235	-4.951337
C	3.887515	3.814331	-4.203976
H	2.946072	4.256926	-4.550548
H	4.182152	4.321427	-3.279814
H	4.649729	4.021235	-4.951337

12^{+}F^{-} ion pair
Stoichiometry: $C_{40}H_{56}N_4O_8FP$
Charge: 0
Multiplicity: 1
Point group: C_2
DLPNO-CCSD(T), Hartree: -2815.2124269
Gibbs in HMPA, Hartree: -2814.4335677
Gibbs in gas, Hartree: -2814.3871509
Nuclear coordinates, Å:

P	0.000000	0.000000	0.300816
C	-0.775732	-1.196728	-0.813245
C	-2.153833	-1.480158	-0.895108
C	0.000000	-1.596525	-1.928747
C	-2.724235	-2.037863	-2.028652
C	-0.561851	-2.127990	-3.066117
C	-1.944605	-2.286902	-3.169401
H	-3.790142	-2.191634	-2.060535
H	0.058330	-2.303641	-3.926342
C	-1.224644	0.730030	1.430781
C	-1.793665	-0.096046	2.412417
C	-1.616639	2.076063	1.441331
C	-2.752460	0.356804	3.304462
C	-2.586026	2.546257	2.321089
C	-3.182344	1.690018	3.252906
H	-3.158973	-0.321656	4.035615
H	-2.856817	3.588142	2.290715
C	0.775732	1.196728	-0.813245
C	2.153833	1.480158	-0.895108
C	0.000000	1.596525	-1.928747
C	2.724235	2.037863	-2.028652
C	0.561851	2.127990	-3.066117
C	1.944605	2.286902	-3.169401
H	3.790142	2.191634	-2.060535
H	-0.058330	2.303641	-3.926342
C	1.224644	-0.730030	1.430781
C	1.793665	0.096046	2.412417
C	1.616639	-2.076063	1.441331
C	2.752460	-0.356804	3.304462
C	2.586026	-2.546257	2.321089
C	3.182344	-1.690018	3.252906
H	3.158973	0.321656	4.035615
H	2.856817	-3.588142	2.290715
O	1.327974	-1.393882	-1.847430
O	-1.349947	-1.366363	2.430864
O	-1.327974	1.393882	-1.847430
O	1.349947	1.366363	2.430864
O	2.929263	1.149067	0.163644
O	-2.929263	-1.149067	0.163644
O	-0.994795	2.902839	0.587894
O	0.994795	-2.902839	0.587894
C	4.324664	1.160872	0.024680
H	4.652507	0.548557	-0.821141
H	4.715457	2.177084	-0.098226
H	4.718352	0.736542	0.947775
C	-4.324664	-1.160872	0.024680
H	-4.652507	-0.548557	-0.821141
H	-4.715457	-2.177084	-0.098226
H	-4.718352	-0.736542	0.947775
C	-1.497926	4.196114	0.376442
H	-2.553774	4.173124	0.090318
H	-1.375428	4.831247	1.260733
H	-0.912934	4.609857	-0.442816
C	1.497926	-4.196114	0.376442
H	2.553774	-4.173124	0.090318
H	1.375428	-4.831247	1.260733
H	0.912934	-4.609857	-0.442816
C	-2.052414	1.351678	-3.076194
H	-1.496086	0.785052	-3.838795
H	-2.997496	0.865280	-2.840336
H	-2.254589	2.368322	-3.431148
C	2.052414	-1.351678	-3.076194
H	1.496086	-0.785052	-3.838795
H	2.997496	-0.865280	-2.840336
H	2.254589	-2.368322	-3.431148
C	-2.001052	-2.316614	3.231192
H	-1.524987	-3.269630	3.009163
H	-1.883374	-2.098346	4.297798
H	-3.065982	-2.379374	2.986964
C	2.001052	2.316614	3.231192
H	1.524987	3.269630	3.009163
H	1.883374	2.098346	4.297798
H	3.065982	2.379374	2.986964
N	2.496858	2.654049	-4.357246

N	-2.496858	-2.654049	-4.357246
N	-4.159082	2.144975	4.102555
N	4.159082	-2.144975	4.102555
C	1.734464	2.484964	-5.585173
H	1.126272	3.370954	-5.809461
H	2.435303	2.347707	-6.409521
H	1.087567	1.591902	-5.526064
C	-1.734464	-2.484964	-5.585173
H	-2.435303	-2.347707	-6.409521
H	-1.087567	-1.591902	-5.526064
H	-1.126272	-3.370954	-5.809461
C	4.624373	-1.303272	5.173316
H	3.829437	-1.044176	5.885384
H	5.410837	-1.821588	5.718386
H	5.049871	-0.371115	4.790383
C	-4.624373	1.303272	5.173316
H	-3.829437	1.044176	5.885384
H	-5.410837	1.821588	5.718386
H	-5.049871	0.371115	4.790383
C	3.902715	2.925683	-4.452779
H	4.228976	3.605191	-3.660202
H	4.521492	2.017698	-4.396869
H	4.106882	3.412038	-5.405773
C	-3.902715	-2.925683	-4.452779
H	-4.228976	-3.605191	-3.660202
H	-4.521492	-2.017698	-4.396869
H	-4.106882	-3.412038	-5.405773
C	4.478135	-3.547945	4.138912
H	3.633761	-4.167645	4.469914
H	4.793944	-3.906658	3.155368
H	5.305799	-3.708069	4.827191
C	-4.478135	3.547945	4.138912
H	-3.633761	4.167645	4.469914
H	-4.793944	3.906658	3.155368
H	-5.305799	3.708069	4.827191
F	0.000000	0.000000	-5.067600

12$^+HF_2^-$ ion pair
Stoichiometry: $C_{40}H_{57}N_4O_8F_2P$
Charge: 0
Multiplicity: 1
Point group: C_1
DLPNO-CCSD(T), Hartree: -2915.6102119
Gibbs in HMPA, Hartree: -2914.8249600
Nuclear coordinates, Å:

P	-0.447093	-0.005042	-0.000930
C	0.711642	0.423300	-1.318777
C	0.986435	1.719675	-1.795638
C	1.673462	-0.561815	-1.624640
C	2.155586	2.013016	-2.472790
C	2.831445	-0.284792	-2.317619
C	3.127688	1.025669	-2.702114
H	2.344979	3.028563	-2.774369
H	3.573831	-1.055190	-2.420222
C	-1.555881	1.368501	0.422118
C	-2.401675	1.845044	-0.589728
C	-1.682843	1.967161	1.682713
C	-3.262864	2.913350	-0.401725
C	-2.534470	3.046623	1.889475
C	-3.320232	3.550153	0.845960
H	-3.886792	3.242731	-1.215683
H	-2.601816	3.477967	2.873974
C	0.668270	-0.484176	1.338849
C	0.856664	-1.779765	1.847181
C	1.684987	0.453131	1.630740
C	1.997529	-2.126037	2.553435
C	2.843904	0.110319	2.293406
C	3.039685	-1.203145	2.734841
H	2.115215	-3.141100	2.891781
H	3.634784	0.835246	2.373896
C	-1.607629	-1.329657	-0.443988
C	-2.504824	-1.756513	0.546031
C	-1.731347	-1.929395	-1.704913
C	-3.413713	-2.781068	0.339374
C	-2.630580	-2.965886	-1.929642
C	-3.469327	-3.422227	-0.906222
H	-4.076673	-3.072355	1.136803
H	-2.693042	-3.398340	-2.913968
O	1.417927	-1.800754	-1.155991
O	-2.315564	1.189490	-1.761437
O	1.457474	1.702623	1.192100
O	-2.419000	-1.099179	1.716730
O	-0.101561	-2.697892	1.586036
O	0.077740	2.685798	-1.527752
O	-0.974176	1.435982	2.691968
O	-0.971165	-1.440843	-2.697087
C	0.092741	-4.028255	1.984217
H	0.994924	-4.460438	1.539599
H	0.155258	-4.122458	3.073937
H	-0.778276	-4.575297	1.625751
C	0.362230	4.011895	-1.884727
H	1.281061	4.373244	-1.412298
H	0.452617	4.130641	-2.970123
H	-0.480359	4.602628	-1.527421
C	-0.853362	2.142127	3.899730
H	-0.489286	3.160399	3.733486
H	-1.802275	2.182202	4.446098
H	-0.124309	1.592896	4.492739
C	-0.850491	-2.160036	-3.897239
H	-0.543747	-3.194215	-3.714500
H	-1.783571	-2.155306	-4.471516
H	-0.076795	-1.652071	-4.470061
C	2.515339	2.648255	1.253867
H	3.408918	2.282511	0.735257
H	2.129937	3.542276	0.766269
H	2.749071	2.890791	2.296404
C	2.503094	-2.712688	-1.074776
H	3.377792	-2.247134	-0.608334
H	2.142694	-3.542673	-0.469356
H	2.765658	-3.091309	-2.068484
C	-2.957194	1.709033	-2.895925
H	-2.664010	1.066296	-3.723459
H	-4.046927	1.685391	-2.791223
H	-2.632650	2.733768	-3.100907
C	-3.133664	-1.566635	2.829579
H	-2.837711	-0.930503	3.661248
H	-4.215244	-1.483093	2.680633
H	-2.876950	-2.605436	3.059227
N	4.217113	-1.568368	3.322560
N	4.328655	1.328509	-3.277310
N	-4.137929	4.633673	1.042303
N	-4.335842	-4.463867	-1.119777
C	5.383775	-0.725445	3.124234
H	5.244076	0.250451	3.596140
H	6.237390	-1.198808	3.606920
H	5.597043	-0.588740	2.058495
C	5.357444	0.306648	-3.294721
H	6.244130	0.718147	-3.774492
H	5.617720	-0.035714	-2.287427
H	5.031507	-0.550393	-3.890076
C	-5.314779	-4.796928	-0.118584
H	-6.017980	-3.976276	0.076188
H	-5.886959	-5.660262	-0.452414
H	-4.838894	-5.064616	0.829343
C	-5.066213	5.025384	0.014501
H	-5.804836	4.244246	-0.209338
H	-5.602768	5.914931	0.338484
H	-4.547584	5.274787	-0.915867
C	4.453961	-2.955604	3.621782
H	3.673489	-3.355509	4.274438
H	4.506806	-3.582767	2.721007
H	5.399701	-3.045842	4.153381
C	4.799100	2.693620	-3.212686
H	4.107549	3.376413	-3.710803
H	4.949499	3.011238	-2.174264
H	5.749189	2.761268	-3.740723
C	-4.486989	-5.009657	-2.442975
H	-4.887353	-4.280485	-3.160184
H	-3.532095	-5.374878	-2.831114
H	-5.170074	-5.855931	-2.404079
C	-4.295673	5.182087	2.363518
H	-4.749778	4.471188	3.066917
H	-3.334237	5.498502	2.777734
H	-4.935139	6.061062	2.311159
F	4.971820	1.356828	-0.137022
H	5.010610	0.228924	-0.113783
F	5.075325	-0.915603	-0.097927

12a, neutral form
Stoichiometry: $C_{40}H_{55}N_4O_8P$
Charge: 0
Multiplicity: 1
Point group: C_1
DLPNO-CCSD(T), Hartree: -2714.8402160
Gibbs in HMPA, Hartree: -2714.0489718
Nuclear coordinates, Å:

P	0.101599	-0.289068	-0.676649
C	-1.116286	-1.685381	-0.584917
C	-2.265732	-1.714563	-1.380126
C	-0.868440	-2.836879	0.167130
C	-3.167618	-2.773326	-1.358519
C	-1.739630	-3.921505	0.187357
C	-2.923155	-3.890816	-0.555186
H	-4.041470	-2.735490	-1.986662

```
H   -1.488785   -4.787465    0.776478
C   -0.642967    1.400973   -1.106063
C   -1.892806    1.831707   -0.647933
C    0.058371    2.314302   -1.895790
C   -2.423927    3.075389   -0.976794
C   -0.443544    3.567431   -2.234534
C   -1.707982    3.960513   -1.788534
H   -3.395530    3.349930   -0.601287
H    0.160704    4.235475   -2.825771
C    1.889084   -0.824566   -0.613260
C    2.784126   -1.055766    0.455709
C    2.469231   -0.886928   -1.881062
C    4.137037   -1.283593    0.244195
C    3.821056   -1.105273   -2.123725
C    4.685136   -1.286689   -1.050474
H    4.768667   -1.492763    1.090954
H    4.144271   -1.142020   -3.153039
C    0.034694    0.344611    1.223761
C    0.879492    1.418354    1.537330
C   -0.802166   -0.033121    2.280011
C    0.913621    2.068852    2.766649
C   -0.792271    0.589939    3.531047
C    0.076871    1.646060    3.799358
H    1.591351    2.893094    2.914560
H   -1.478007    0.255531    4.291193
O    0.277008   -2.844985    0.872140
O   -2.580005    0.960388    0.119582
O    1.699073   -0.720487   -2.963126
O    1.721046    1.802575    0.543078
O    2.287204   -1.088181    1.708501
O   -2.448945   -0.660758   -2.207188
O    1.288419    1.923151   -2.299550
O   -1.680186   -1.048368    2.060592
C    3.162370   -1.086502    2.802742
H    3.711547   -2.031822    2.892824
H    3.882253   -0.263660    2.744854
H    2.536038   -0.951235    3.683313
C   -3.611011   -0.583815   -2.985804
H   -3.672421   -1.404076   -3.709319
H   -4.514203   -0.585064   -2.365923
H   -3.549447    0.361539   -3.522419
C    1.958345    2.650913   -3.291873
H    1.336133    2.789823   -4.182219
H    2.284213    3.632714   -2.928707
H    2.833370    2.058978   -3.554847
C   -2.478985   -1.518861    3.105389
H   -1.883175   -1.866332    3.958055
H   -3.187082   -0.760542    3.462314
H   -3.038785   -2.359794    2.696154
C    0.325299   -0.687143   -2.614237
H   -0.083071   -1.680872   -2.816014
H   -0.162173    0.039346   -3.259543
C    0.559311   -3.920834    1.721158
H    0.676777   -4.858601    1.166815
H    1.501164   -3.675235    2.207575
H   -0.216883   -4.049821    2.483697
C   -3.795972    1.351730    0.691004
H   -4.130665    0.504286    1.286803
H   -3.676781    2.224945    1.341241
H   -4.553862    1.572474   -0.069868
C    2.533007    2.928393    0.708120
H    3.070247    3.049355   -0.231348
H    1.943372    3.830785    0.905394
H    3.261549    2.794151    1.516144
N    6.037105   -1.474340   -1.241337
N   -3.823278   -4.937165   -0.501380
N   -2.240511    5.185471   -2.145144
N    0.119606    2.245172    5.051573
C    6.525409   -1.705448   -2.575158
H    6.265394   -0.874098   -3.234748
H    7.611770   -1.778243   -2.550259
H    6.127389   -2.626507   -3.023967
C   -3.422396   -6.173725    0.116017
H   -4.260427   -6.868856    0.097256
H   -2.569185   -6.650265   -0.387852
H   -3.150243   -6.019592    1.163219
C    0.788297    3.512376    5.183356
H    0.342678    4.305014    4.563508
H    0.747416    3.829581    6.224860
H    1.843077    3.428828    4.911134
C   -3.402210    5.671151   -1.448897
H   -3.236235    5.796054   -0.369091
H   -3.687873    6.636656   -1.863760
H   -4.251941    4.996252   -1.581328
C    6.859007   -1.907742   -0.143120
H    6.808624   -1.200894    0.688813
H    6.581135   -2.900535    0.239270
H    7.896660   -1.949618   -0.471234
C   -4.891901   -4.991699   -1.463456
H   -5.525141   -4.103450   -1.396662
H   -4.535229   -5.075499   -2.500264
H   -5.520119   -5.854974   -1.249049
C   -0.947632    1.979296    5.978296
H   -1.933554    2.303798    5.611420
H   -1.010671    0.912655    6.206380
H   -0.744273    2.498885    6.914098
C   -1.390505    6.158508   -2.777350
H   -0.549357    6.475143   -2.143038
H   -0.977852    5.769646   -3.711616
H   -1.980494    7.039807   -3.024887
```

12a·HCN complex, transition state 1
Stoichiometry: $C_{41}H_{56}N_5O_8P$
Charge: 0
Multiplicity: 1
Point group: C_1
DLPNO-CCSD(T), Hartree: -2808.0946980
Gibbs in HMPA, Hartree: -2807.2973625
Gibbs in gas, Hartree: -2807.2718730
Nuclear coordinates, Å:

```
P    0.951044    1.048331    0.937298
C   -0.120897    2.399162    0.420188
C   -0.817848    3.094693    1.417722
C   -0.301759    2.828935   -0.900223
C   -1.702037    4.118173    1.129845
C   -1.176126    3.863591   -1.208189
C   -1.909540    4.503351   -0.204186
H   -2.216077    4.620215    1.932173
H   -1.286039    4.163602   -2.236342
C    0.152858   -0.251214    1.898539
C   -1.237292   -0.403922    2.060600
C    0.969287   -1.193317    2.536242
C   -1.764622   -1.474095    2.763682
C    0.459230   -2.268626    3.243958
C   -0.928747   -2.441705    3.339308
H   -2.833988   -1.566351    2.851676
H    1.132491   -2.974545    3.700456
C    2.419767    0.709677   -0.053138
C    2.673231    0.102581   -1.306772
C    3.553942    1.088794    0.680584
C    3.975976   -0.114499   -1.737108
C    4.856678    0.891051    0.262439
C    5.085889    0.261857   -0.962810
H    4.127450   -0.562604   -2.704217
H    5.651930    1.238063    0.903559
C   -0.912611   -0.869085   -0.996903
C   -0.427515   -2.157381   -1.186784
C   -1.956487   -0.566863   -1.859251
C   -0.909281   -3.082678   -2.124104
C   -2.490786   -1.426219   -2.831493
C   -1.951598   -2.708457   -2.981077
H   -0.493516   -4.076216   -2.193274
H   -3.311903   -1.118245   -3.460408
O    0.435490    2.220952   -1.831095
O   -2.016618    0.548593    1.540295
O    3.394144    1.671942    1.881483
O    0.632794   -2.513055   -0.369488
O    1.636453   -0.224514   -2.066941
O   -0.560392    2.701769    2.686378
O    2.299555   -0.984148    2.408882
O   -2.494700    0.705750   -1.712584
C    1.831018   -0.980273   -3.234648
H    2.375601   -0.412115   -3.998217
H    2.370295   -1.908772   -3.024051
H    0.832962   -1.223421   -3.593053
C   -1.382001    3.167595    3.727616
H   -1.257606    4.243013    3.890394
H   -2.434230    2.948355    3.526345
H   -1.068181    2.633027    4.622066
C    3.203128   -1.909884    2.953479
H    3.113230   -1.968145    4.043210
H    3.060297   -2.907103    2.526345
H    4.195350   -1.546153    2.693039
C   -3.615156    1.053776   -2.470370
H   -3.411707    1.054846   -3.550200
H   -4.466772    0.386558   -2.286229
H   -3.890150    2.066034   -2.168742
C    2.040055    1.908732    2.199277
H    1.849982    2.981402    2.165470
H    1.837649    1.531126    3.198704
C    0.125151    2.416884   -3.187329
H    0.335989    3.443572   -3.506014
H    0.766844    1.734807   -3.739640
H   -0.920154    2.169158   -3.386046
```

```
C   -3.409283    0.369302    1.500992
H   -3.797424    1.218687    0.943139
H   -3.680888   -0.551477    0.982064
H   -3.843596    0.361284    2.507511
C    1.119334   -3.820032   -0.426097
H    1.916215   -3.887780    0.315930
H    0.349378   -4.562926   -0.182232
H    1.540233   -4.071084   -1.409196
N    6.358545    0.024399   -1.405158
N   -2.805335    5.494730   -0.512341
N   -1.456533   -3.519028    3.993180
N   -2.426819   -3.582161   -3.963451
C    7.477942    0.547593   -0.665910
H    7.489952    0.166021    0.359220
H    8.402311    0.233343   -1.146383
H    7.473083    1.643896   -0.616551
C   -2.893671    5.983849   -1.863453
H   -3.678567    6.735430   -1.920277
H   -1.957073    6.440629   -2.209575
H   -3.154196    5.181373   -2.559437
C   -2.102442   -4.978732   -3.836755
H   -2.473031   -5.429800   -2.903041
H   -2.538804   -5.521506   -4.675323
H   -1.022036   -5.134766   -3.877268
C   -2.886069   -3.661493    4.124895
H   -3.381457   -3.658913    3.150772
H   -3.104195   -4.614171    4.603300
H   -3.327307   -2.865943    4.739452
C    6.579941   -0.505168   -2.725522
H    6.078105   -1.468001   -2.857129
H    6.228938    0.170506   -3.516215
H    7.646193   -0.668208   -2.869482
C   -3.428038    6.252434    0.540988
H   -3.995672    5.604136    1.214888
H   -2.703176    6.815876    1.143604
H   -4.127763    6.962176    0.104218
C   -3.703698   -3.292785   -4.561256
H   -4.529509   -3.264167   -3.833375
H   -3.683085   -2.332499   -5.081486
H   -3.928971   -4.057911   -5.304243
C   -0.588926   -4.443009    4.671325
H    0.116639   -4.917228    3.980186
H   -0.007023   -3.961842    5.468233
H   -1.188342   -5.230672    5.122708
H   -2.611086   -2.513460   -0.405132
C   -3.430127   -3.050094    0.059089
N   -4.285325   -3.602137    0.591320
```

12a·HCN complex, transition state 2

Stoichiometry: $C_{41}H_{56}N_5O_8P$

Charge: 0

Multiplicity: 1

Point group: C_1

DLPNO-CCSD(T), Hartree: -2808.0814938

Gibbs in HMPA, Hartree: -2807.2940445

Gibbs in gas, Hartree: -2807.2590838

Nuclear coordinates, Å:

```
P    0.108693    0.044646   -0.160606
C    1.804806   -0.619607   -0.059882
C    2.353198   -1.636258   -0.860452
C    2.695506    0.019487    0.812412
C    3.681308   -2.019058   -0.744047
C    4.023424   -0.355300    0.945442
C    4.539933   -1.389455    0.165970
H    4.057329   -2.790577   -1.393954
H    4.662434    0.214032    1.598290
C   -1.007904   -1.025683   -1.151074
C   -1.049953   -2.393362   -0.829174
C   -1.898262   -0.604824   -2.149627
C   -1.833311   -3.302884   -1.523006
C   -2.689864   -1.503117   -2.857094
C   -2.649159   -2.871146   -2.575444
H   -1.813414   -4.340818   -1.236388
H   -3.352823   -1.121912   -3.615505
C    0.021702    1.839463   -0.512388
C   -0.261812    2.770026    0.527764
C    0.109619    2.420880   -1.809461
C   -0.553829    4.101126    0.298681
C   -0.212222    3.764106   -2.033414
C   -0.564889    4.620124   -1.005583
H   -0.737489    4.746589    1.139796
H   -0.091528    4.102378   -3.050985
C   -0.749145   -0.260990    1.464087
C   -2.086986    0.153443    1.565180
C   -0.245978   -0.947391    2.577994
C   -2.872474   -0.066221    2.687221
C   -1.013280   -1.168001    3.719895
C   -2.334091   -0.720923    3.799531
H   -3.893461    0.276630    2.693177
H   -0.579905   -1.714905    4.539919
O    2.174784    1.034667    1.523823
O   -0.279102   -2.777157    0.204138
O    0.466850    1.808827   -2.923159
O   -2.568966    0.811522    0.492850
O   -0.228443    2.314723    1.797628
O    1.552140   -2.220405   -1.772868
O   -1.986605    0.713405   -2.380151
O    1.010409   -1.424927    2.506109
C   -0.679360    3.124663    2.847398
H   -0.021961    3.986256    3.011103
H   -1.699219    3.484475    2.675116
H   -0.667236    2.492858    3.734441
C    2.119055   -3.015081   -2.780869
H    2.899523   -2.474279   -3.323939
H    2.535719   -3.947979   -2.383823
H    1.304558   -3.254078   -3.462858
C   -2.542210    1.172635   -3.587465
H   -2.087793    0.673584   -4.447382
H   -3.628853    1.033455   -3.612388
H   -2.314457    2.235943   -3.628729
C    1.616104   -1.977601    3.642750
H    1.610439   -1.279005    4.485968
H    1.134401   -2.912786    3.950832
H    2.645905   -2.184207    3.355455
C    1.162503    0.548992   -2.809807
H    1.820778    0.528897   -3.690674
H    0.439770   -0.254358   -2.970244
C    3.045492    1.959850    2.132674
H    3.764090    2.350814    1.408060
H    2.411739    2.769399    2.489610
H    3.568792    1.515822    2.987028
C   -0.101601   -4.141397    0.471899
H    0.633077   -4.193309    1.273136
H   -1.031583   -4.613837    0.805588
H    0.281954   -4.673571   -0.404367
C   -3.906410    1.231581    0.466093
H   -4.044131    1.710521   -0.500978
H   -4.596506    0.386130    0.555248
H   -4.118315    1.956840    1.258740
N   -0.887213    5.937780   -1.238944
N    5.858272   -1.770231    0.275922
N   -3.398583   -3.766005   -3.299872
N   -3.083118   -0.916024    4.937699
C   -0.647963    6.489873   -2.547803
H   -1.182446    5.920622   -3.311343
H   -1.021746    7.512491   -2.576541
H    0.416361    6.501101   -2.819404
C    6.770372   -0.912479    0.991564
H    7.762440   -1.360918    0.979238
H    6.836513    0.092387    0.554948
H    6.471533   -0.809345    2.038052
C   -4.496804   -0.648274    4.907975
H   -5.035613   -1.275082    4.183912
H   -4.915508   -0.833896    5.895501
H   -4.695842    0.397996    4.660238
C   -3.501566   -5.131213   -2.857153
H   -3.969562   -5.224725   -1.867507
H   -4.102268   -5.692835   -3.570219
H   -2.518097   -5.607253   -2.810471
C   -0.956187    6.858239   -0.134211
H   -1.696687    6.534938    0.601267
H    0.005428    6.983917    0.383117
H   -1.271645    7.832701   -0.504389
C    6.418102   -2.660296   -0.707461
H    5.876939   -3.609641   -0.730371
H    6.410099   -2.238684   -1.722249
H    7.449813   -2.881852   -0.438892
C   -2.573454   -1.761444    5.984382
H   -2.424525   -2.801378    5.661124
H   -1.618433   -1.387204    6.362820
H   -3.275704   -1.761897    6.816197
C   -4.360084   -3.280512   -4.254101
H   -5.165636   -2.695345   -3.789134
H   -3.881138   -2.652702   -5.010090
H   -4.810142   -4.127209   -4.769282
H    2.387913    1.085911   -1.936409
C    3.322491    1.806042   -1.548219
N    4.188967    2.489608   -1.206852
```

13, neutral form

Stoichiometry: $C_{14}H_{21}N_5$

Charge: 0

Multiplicity: 1

Point group: C_S

```
DLPNO-CCSD(T), Hartree:  -818.3307370
Gibbs in HMPA, Hartree:  -818.0627293
Nuclear coordinates, Å:
C   -0.001426    1.164575    0.731868
C   -0.001426    1.164575   -0.731868
C    0.003008   -1.291549   -1.278362
C    0.003008   -1.291549    1.278362
C   -0.018555    3.399255    0.000000
N   -0.010597    2.433542   -1.116721
N   -0.010597    2.433542    1.116721
N   -0.028703    4.608419    0.000000
C   -0.021474    2.928710    2.455619
H   -0.915404    2.602608    2.996062
H    0.865413    2.604770    3.008497
H   -0.023411    4.017277    2.357536
C   -0.021474    2.928710   -2.455619
H    0.865413    2.604770   -3.008497
H   -0.915404    2.602608   -2.996062
H   -0.023411    4.017277   -2.357536
N    0.009794   -2.154601    2.347110
N    0.009794   -2.154601   -2.347110
C   -0.131277   -3.577893    2.161863
H   -0.286449   -4.046605    3.130907
H   -0.996848   -3.814331    1.536683
H    0.755514   -4.035715    1.706140
C   -0.131277   -3.577893   -2.161863
H    0.755514   -4.035715   -1.706140
H   -0.996848   -3.814331   -1.536683
H   -0.286449   -4.046605   -3.130907
C    0.171540   -1.674687    3.700005
H    1.042932   -1.021285    3.792737
H   -0.707131   -1.127622    4.060564
H    0.327236   -2.525867    4.358751
C    0.171540   -1.674687   -3.700005
H   -0.707131   -1.127622   -4.060564
H    1.042932   -1.021285   -3.792737
H    0.327236   -2.525867   -4.358751
C   -0.013829    0.084693    1.591129
C   -0.013829    0.084693   -1.591129
C    0.020730   -1.871978    0.000000
H   -0.054784    0.350884    2.635137
H   -0.054784    0.350884   -2.635137
H    0.106479   -2.942160    0.000000
```

13, protonated form
Stoichiometry: $C_{14}H_{22}N_5^+$
Charge: 1
Multiplicity: 1
Point group: C_S

```
DLPNO-CCSD(T), Hartree:  -818.8124824
Gibbs in HMPA, Hartree:  -818.5629786
Nuclear coordinates, Å:
C    0.000000    1.325640    0.000000
C   -1.213412    0.536694    0.000000
C   -0.353329   -1.815029    0.000000
C    1.800514   -0.415358    0.000000
C   -1.787427    2.737512    0.000000
N   -2.243481    1.421823    0.000000
N   -0.394410    2.633118    0.000000
N   -2.563722    3.728357    0.000000
C    0.504208    3.755459    0.000000
H    1.134863    3.747543    0.891664
H    1.134863    3.747543   -0.891664
H   -0.072996    4.676538    0.000000
C   -3.645550    1.091424    0.000000
H   -3.905127    0.517763   -0.892134
H   -3.905127    0.517763    0.892134
H   -4.197736    2.028093    0.000000
N    3.152433   -0.544163    0.000000
N   -0.780188   -3.103358    0.000000
C    3.775751   -1.852043    0.000000
H    4.854469   -1.729881    0.000000
H    3.503889   -2.428316    0.889414
H    3.503889   -2.428316   -0.889414
C    0.163009   -4.203368    0.000000
H    0.799768   -4.189023   -0.889529
H    0.799768   -4.189023    0.889529
H   -0.386350   -5.139708    0.000000
C    4.030191    0.611830    0.000000
H    3.889522    1.230623   -0.890399
H    3.889522    1.230623    0.890399
H    5.059962    0.268103    0.000000
C   -2.193024   -3.437539    0.000000
H   -2.701021   -3.056919    0.890104
H   -2.701021   -3.056919   -0.890104
H   -2.296277   -4.518223    0.000000
C    1.296010    0.921026    0.000000
C   -1.368507   -0.814411    0.000000
C    1.032910   -1.588228    0.000000
H    2.025071    1.715300    0.000000
H   -2.391807   -1.154024    0.000000
H    1.617993   -2.488185    0.000000
H   -2.098606    4.626542    0.000000
```

14a, deprotonated form
Stoichiometry: $C_{26}H_{36}BN_8^-$
Charge: −1
Multiplicity: 1
Point group: D_{2d}

```
DLPNO-CCSD(T), Hartree: -1472.7625008
Gibbs in HMPA, Hartree: -1472.3126458
Nuclear coordinates, Å:
B    0.000000    0.000000    0.000000
N    0.000000   -1.219428    0.947858
N   -1.219428    0.000000   -0.947858
N    1.219428    0.000000   -0.947858
N    0.000000    1.219428    0.947858
C    0.000000    0.000000    3.125806
C    0.000000    0.000000   -3.125806
C    0.000000    1.123861    2.327748
C    1.123861    0.000000   -2.327748
C    0.000000   -1.123861    2.327748
C   -1.123861    0.000000   -2.327748
C    0.000000    2.561676    0.578674
C    0.000000   -2.561676    0.578674
C   -2.561676    0.000000   -0.578674
C    2.561676    0.000000   -0.578674
N    2.429736    0.000000   -2.781661
N    0.000000   -2.429736    2.781661
N   -2.429736    0.000000   -2.781661
N    0.000000    2.429736    2.781661
C    0.000000   -3.309139    1.709055
C    3.309139    0.000000   -1.709055
C   -3.309139    0.000000   -1.709055
C    0.000000    3.309139    1.709055
C    0.000000   -2.751812    4.172870
H   -0.889466   -3.323337    4.461716
H    0.889466   -3.323337    4.461716
H    0.000000   -1.788106    4.691130
C   -2.751812    0.000000   -4.172870
H   -3.323337   -0.889466   -4.461716
H   -3.323337    0.889466   -4.461716
H   -1.788106    0.000000   -4.691130
C    2.751812    0.000000   -4.172870
H    3.323337    0.889466   -4.461716
H    3.323337   -0.889466   -4.461716
H    1.788106    0.000000   -4.691130
C    0.000000    2.751812    4.172870
H    0.889466    3.323337    4.461716
H   -0.889466    3.323337    4.461716
H    0.000000    1.788106    4.691130
C    4.777941    0.000000   -1.902224
H    5.121227   -0.880154   -2.458233
H    5.121227    0.880154   -2.458233
H    5.285206    0.000000   -0.937778
C    0.000000   -4.777941    1.902224
H    0.880154   -5.121227    2.458233
H   -0.880154   -5.121227    2.458233
H    0.000000   -5.285206    0.937778
C   -4.777941    0.000000   -1.902224
H   -5.121227    0.880154   -2.458233
H   -5.121227   -0.880154   -2.458233
H   -5.285206    0.000000   -0.937778
C    0.000000    4.777941    1.902224
H   -0.880154    5.121227    2.458233
H    0.880154    5.121227    2.458233
H    0.000000    5.285206    0.937778
C   -3.011428    0.000000    0.835427
H   -2.642953   -0.872630    1.380018
H   -2.642953    0.872630    1.380018
H   -4.101402    0.000000    0.885670
C    0.000000    3.011428   -0.835427
H    0.872630    2.642953   -1.380018
H   -0.872630    2.642953   -1.380018
H    0.000000    4.101402   -0.885670
C    0.000000   -3.011428   -0.835427
H   -0.872630   -2.642953   -1.380018
H    0.872630   -2.642953   -1.380018
H    0.000000   -4.101402   -0.885670
C    3.011428    0.000000    0.835427
H    2.642953    0.872630    1.380018
H    2.642953   -0.872630    1.380018
H    4.101402    0.000000    0.885670
```

14a, neutral form
Stoichiometry: $C_{26}H_{37}BN_8$
Charge: 0
Multiplicity: 1
Point group: C_s
DLPNO-CCSD(T), Hartree: -1473.3810878
Gibbs in HMPA, Hartree: -1472.8576307
Nuclear coordinates, Å:

B	-0.007512	0.042824	0.000000
N	1.281249	0.844548	0.000000
N	-0.091233	-0.931757	1.235779
N	-0.091233	-0.931757	-1.235779
N	-1.145506	1.039266	0.000000
C	0.239756	3.113949	0.000000
C	-0.114370	-3.022474	0.000000
C	-0.942659	2.412674	0.000000
C	-0.111257	-2.280085	-1.179406
C	1.297306	2.234433	0.000000
C	-0.111257	-2.280085	1.179406
C	-2.519234	0.776690	0.000000
C	2.598899	0.372965	0.000000
C	-0.110774	-0.551169	2.576098
C	-0.110774	-0.551169	-2.576098
H	-0.123572	-4.098815	0.000000
N	-0.132064	-2.755612	-2.446659
N	2.632273	2.579763	0.000000
N	-0.132064	-2.755612	2.446659
N	-2.207865	2.962219	0.000000
C	3.424047	1.446474	0.000000
C	-0.133148	-1.675327	-3.332125
C	-0.133148	-1.675327	3.332125
C	-3.167157	1.966407	0.000000
C	3.069353	3.943987	0.000000
H	2.156731	4.544506	0.000000
H	3.662127	4.177993	0.889298
H	3.662127	4.177993	-0.889298
C	-0.158274	-4.152905	2.771792
H	-1.043212	-4.634852	2.346860
H	0.730756	-4.660558	2.387319
H	-0.184613	-4.273219	3.852051
C	-0.158274	-4.152905	-2.771792
H	0.730756	-4.660558	-2.387319
H	-1.043212	-4.634852	-2.346860
H	-0.184613	-4.273219	-3.852051
C	-2.426122	4.378081	0.000000
H	-1.430498	4.827901	0.000000
H	-2.974844	4.702210	-0.889282
H	-2.974844	4.702210	0.889282
C	-0.163228	-1.857724	-4.800769
H	0.698461	-2.425719	-5.165587
H	-1.066968	-2.376334	-5.136632
H	-0.145164	-0.885714	-5.290634
C	4.903309	1.532596	0.000000
H	5.278824	2.064594	-0.879785
H	5.278824	2.064594	0.879785
H	5.346654	0.537854	0.000000
C	-0.163228	-1.857724	4.800769
H	-1.066968	-2.376334	5.136632
H	0.698461	-2.425719	5.165587
H	-0.145164	-0.885714	5.290634
C	-4.615096	2.281732	0.000000
H	-4.903311	2.865783	0.879645
H	-4.903311	2.865783	-0.879645
H	-5.207536	1.367858	0.000000
C	-0.139838	0.868306	3.003795
H	0.622354	1.462460	2.497534
H	-1.099866	1.333898	2.768483
H	0.021056	0.942201	4.079029
C	-3.093409	-0.591559	0.000000
H	-2.788765	-1.167485	-0.878553
H	-2.788765	-1.167485	0.878553
H	-4.182183	-0.539693	0.000000
C	2.958886	-1.065554	0.000000
H	2.571784	-1.589532	0.878433
H	2.571784	-1.589532	-0.878433
H	4.043011	-1.178148	0.000000
C	-0.139838	0.868306	-3.003795
H	-1.099866	1.333898	-2.768483
H	0.622354	1.462460	-2.497534
H	0.021056	0.942201	-4.079029

14a, protonated form
Stoichiometry: $C_{26}H_{38}BN_8^+$
Charge: +1
Multiplicity: 1
Point group: D_{2d}
DLPNO-CCSD(T), Hartree: -1473.9073738
Gibbs in HMPA, Hartree: -1473.3935618
Nuclear coordinates, Å:

B	0.000000	0.000000	0.000000
N	0.000000	-1.233637	0.932790
N	-1.233637	0.000000	-0.932790
N	1.233637	0.000000	-0.932790
N	0.000000	1.233637	0.932790
C	0.000000	0.000000	3.026233
C	0.000000	0.000000	-3.026233
C	0.000000	1.177847	2.287234
C	1.177847	0.000000	-2.287234
C	0.000000	-1.177847	2.287234
C	-1.177847	0.000000	-2.287234
C	0.000000	2.583033	0.556892
C	0.000000	-2.583033	0.556892
C	-2.583033	0.000000	-0.556892
C	2.583033	0.000000	-0.556892
H	0.000000	0.000000	-4.102377
H	0.000000	0.000000	4.102377
N	2.442898	0.000000	-2.760458
N	0.000000	-2.442898	2.760458
N	-2.442898	0.000000	-2.760458
N	0.000000	2.442898	2.760458
C	0.000000	-3.330513	1.685613
C	3.330513	0.000000	-1.685613
C	-3.330513	0.000000	-1.685613
C	0.000000	3.330513	1.685613
C	0.000000	-2.768849	4.162571
H	0.888132	-2.364466	4.653541
H	-0.888132	-2.364466	4.653541
H	0.000000	-3.848727	4.282534
C	-2.768849	0.000000	-4.162571
H	-2.364466	0.888132	-4.653541
H	-2.364466	-0.888132	-4.653541
H	-3.848727	0.000000	-4.282534
C	2.768849	0.000000	-4.162571
H	2.364466	-0.888132	-4.653541
H	2.364466	0.888132	-4.653541
H	3.848727	0.000000	-4.282534
C	0.000000	2.768849	4.162571
H	-0.888132	2.364466	4.653541
H	0.888132	2.364466	4.653541
H	0.000000	3.848727	4.282534
C	4.798428	0.000000	-1.878518
H	5.138958	-0.882913	-2.426560
H	5.138958	0.882913	-2.426560
H	5.302513	0.000000	-0.914189
C	0.000000	-4.798428	1.878518
H	0.882913	-5.138958	2.426560
H	-0.882913	-5.138958	2.426560
H	0.000000	-5.302513	0.914189
C	-4.798428	0.000000	-1.878518
H	-5.138958	0.882913	-2.426560
H	-5.138958	-0.882913	-2.426560
H	-5.302513	0.000000	-0.914189
C	0.000000	4.798428	1.878518
H	-0.882913	5.138958	2.426560
H	0.882913	5.138958	2.426560
H	0.000000	5.302513	0.914189
C	-3.032990	0.000000	0.855206
H	-2.678172	-0.879840	1.395939
H	-2.678172	0.879840	1.395939
H	-4.120794	0.000000	0.898440
C	0.000000	3.032990	-0.855206
H	0.879840	2.678172	-1.395939
H	-0.879840	2.678172	-1.395939
H	0.000000	4.120794	-0.898440
C	0.000000	-3.032990	-0.855206
H	-0.879840	-2.678172	-1.395939
H	0.879840	-2.678172	-1.395939
H	0.000000	-4.120794	-0.898440
C	3.032990	0.000000	0.855206
H	2.678172	0.879840	1.395939
H	2.678172	-0.879840	1.395939
H	4.120794	0.000000	0.898440

14a, diprotonated form
Stoichiometry: $C_{26}H_{39}BN_8^{2+}$
Charge: +2
Multiplicity: 1
Point group: C_s
DLPNO-CCSD(T), Hartree: -1474.2322835
Gibbs in HMPA, Hartree: -1473.8453471
Nuclear coordinates, Å:

B	0.000143	0.042213	0.000000
N	-0.063782	-0.923771	1.251464
N	1.286639	0.847285	0.000000

```
N   -1.171889    0.999822    0.000000
N   -0.063782   -0.923771   -1.251464
C   -0.043741   -3.087804    0.000000
C    0.185605    3.016056    0.000000
C   -0.070140   -2.252837   -1.223729
C   -1.034931    2.355025    0.000000
C   -0.070140   -2.252837    1.223729
C    1.316227    2.209996    0.000000
C   -0.098442   -0.521657   -2.581027
C   -0.098442   -0.521657    2.581027
C    2.619254    0.391181    0.000000
C   -2.548848    0.706454    0.000000
H    0.252043    4.090303    0.000000
H   -0.902458   -3.769040    0.000000
N   -2.268391    2.898541    0.000000
N   -0.103663   -2.720957    2.472076
N    2.606278    2.600255    0.000000
N   -0.103663   -2.720957   -2.472076
C   -0.122146   -1.649527    3.347767
C   -3.219684    1.882331    0.000000
C    3.427247    1.476850    0.000000
C   -0.122146   -1.649527   -3.347767
C   -0.119227   -4.113904    2.879421
H    0.747223   -4.328501    3.503878
H   -1.026860   -4.327869    3.442738
H   -0.088097   -4.758318    2.005286
C    3.021353    3.984151    0.000000
H    2.649044    4.495842   -0.889271
H    2.649044    4.495842    0.889271
H    4.106120    4.034939    0.000000
C   -2.513337    4.322501    0.000000
H   -2.081898    4.785518    0.889214
H   -2.081898    4.785518   -0.889214
H   -3.584045    4.503977    0.000000
C   -0.119227   -4.113904   -2.879421
H   -1.026860   -4.327869   -3.442738
H    0.747223   -4.328501   -3.503878
H   -0.088097   -4.758318   -2.005286
C   -4.672294    2.168766    0.000000
H   -4.971615    2.739367    0.882608
H   -4.971615    2.739367   -0.882608
H   -5.243575    1.243011    0.000000
C   -0.165810   -1.836512    4.815344
H   -1.067743   -2.368311    5.127874
H    0.696643   -2.401739    5.176447
H   -0.162567   -0.872596    5.318966
C    4.903716    1.586059    0.000000
H    5.269318    2.116373   -0.882770
H    5.269318    2.116373    0.882770
H    5.359881    0.598512    0.000000
C   -0.165810   -1.836512   -4.815344
H    0.696643   -2.401739   -5.176447
H   -1.067743   -2.368311   -5.127874
H   -0.162567   -0.872596   -5.318966
C    3.001712   -1.040713    0.000000
H    2.639463   -1.567140    0.887485
H    2.639463   -1.567140   -0.887485
H    4.086263   -1.129886    0.000000
C   -0.127452    0.893899   -3.018020
H   -1.073970    1.372510   -2.759939
H    0.674914    1.476374   -2.565417
H   -0.011041    0.948846   -4.098530
C   -0.127452    0.893899    3.018020
H    0.674914    1.476374    2.565417
H   -1.073970    1.372510    2.759939
H   -0.011041    0.948846    4.098530
C   -3.096203   -0.671450    0.000000
H   -2.795847   -1.237270   -0.886157
H   -2.795847   -1.237270    0.886157
H   -4.183795   -0.634180    0.000000
H    0.852818   -3.719970    0.000000
```

14a, triprotonated form
Stoichiometry: $C_{26}H_{40}BN_8^{3+}$
Charge: +3
Multiplicity: 1
Point group: D_{2d}
DLPNO-CCSD(T), Hartree: -1474.4531297
Gibbs in HMPA, Hartree: -1474.2855808
Nuclear coordinates, Å:

```
B    0.000000    0.000000    0.000000
N    0.000000   -1.252042    0.915900
N   -1.252042    0.000000   -0.915900
N    1.252042    0.000000   -0.915900
N    0.000000    1.252042    0.915900
C    0.000000    0.000000    3.085814
C    0.000000    0.000000   -3.085814
C    0.000000    1.224458    2.252365
C    1.224458    0.000000   -2.252365
C    0.000000   -1.224458    2.252365
C   -1.224458    0.000000   -2.252365
C    0.000000    2.590293    0.517676
C    0.000000   -2.590293    0.517676
C   -2.590293    0.000000   -0.517676
C    2.590293    0.000000   -0.517676
H    0.000000    0.876641   -3.744896
H    0.876641    0.000000    3.744896
N    2.469800    0.000000   -2.717068
N    0.000000   -2.469800    2.717068
N   -2.469800    0.000000   -2.717068
N    0.000000    2.469800    2.717068
C    0.000000   -3.351362    1.649122
C    3.351362    0.000000   -1.649122
C   -3.351362    0.000000   -1.649122
C    0.000000    3.351362    1.649122
C    0.000000   -2.879858    4.114757
H   -0.888043   -3.474983    4.323704
H    0.888043   -3.474983    4.323704
H    0.000000   -2.006810    4.761097
C   -2.879858    0.000000   -4.114757
H   -3.474983   -0.888043   -4.323704
H   -3.474983    0.888043   -4.323704
H   -2.006810    0.000000   -4.761097
C    2.879858    0.000000   -4.114757
H    3.474983    0.888043   -4.323704
H    3.474983   -0.888043   -4.323704
H    2.006810    0.000000   -4.761097
C    0.000000    2.879858    4.114757
H    0.888043    3.474983    4.323704
H   -0.888043    3.474983    4.323704
H    0.000000    2.006810    4.761097
C    4.817786    0.000000   -1.846627
H    5.147300   -0.882377   -2.400288
H    5.147300    0.882377   -2.400288
H    5.335549    0.000000   -0.890330
C    0.000000   -4.817786    1.846627
H    0.882377   -5.147300    2.400288
H   -0.882377   -5.147300    2.400288
H    0.000000   -5.335549    0.890330
C   -4.817786    0.000000   -1.846627
H   -5.147300    0.882377   -2.400288
H   -5.147300   -0.882377   -2.400288
H   -5.335549    0.000000   -0.890330
C    0.000000    4.817786    1.846627
H   -0.882377    5.147300    2.400288
H    0.882377    5.147300    2.400288
H    0.000000    5.335549    0.890330
C   -3.047930    0.000000    0.892414
H   -2.714653   -0.888820    1.431703
H   -2.714653    0.888820    1.431703
H   -4.135571    0.000000    0.921359
C    0.000000    3.047930   -0.892414
H    0.888820    2.714653   -1.431703
H   -0.888820    2.714653   -1.431703
H    0.000000    4.135571   -0.921359
C    0.000000   -3.047930   -0.892414
H   -0.888820   -2.714653   -1.431703
H    0.888820   -2.714653   -1.431703
H    0.000000   -4.135571   -0.921359
C    3.047930    0.000000    0.892414
H    2.714653    0.888820    1.431703
H    2.714653   -0.888820    1.431703
H    4.135571    0.000000    0.921359
H   -0.876641    0.000000    3.744896
H    0.000000   -0.876641   -3.744896
```

14b, deprotonated form
Stoichiometry: $C_{34}H_{60}BN_{16}^{-}$
Charge: −1
Multiplicity: 1
Point group: S_4
DLPNO-CCSD(T), Hartree: -2228.6465816
Gibbs in HMPA, Hartree: -2227.8709144
Nuclear coordinates, Å:

```
B    0.000000    0.000000    0.000000
N   -0.010509    1.219767    0.945316
N    1.219767    0.010509   -0.945316
N   -1.219767   -0.010509   -0.945316
N    0.010509   -1.219767    0.945316
C    0.000000    0.000000    3.119025
C    0.000000    0.000000   -3.119025
C    0.008061   -1.124862    2.322848
C   -1.124862   -0.008061   -2.322848
C   -0.008061    1.124862    2.322848
```

C	1.124862	0.008061	-2.322848
C	0.000000	-2.556516	0.575991
C	0.000000	2.556516	0.575991
C	2.556516	0.000000	-0.575991
C	-2.556516	0.000000	-0.575991
N	-2.431227	0.001521	-2.784871
N	0.001521	2.431227	2.784871
N	2.431227	-0.001521	-2.784871
N	-0.001521	-2.431227	2.784871
C	0.002626	3.313873	1.704852
C	-3.313873	0.002626	-1.704852
C	3.313873	-0.002626	-1.704852
C	-0.002626	-3.313873	1.704852
N	0.039385	2.947281	-0.767007
N	2.947281	-0.039385	0.767007
N	-0.039385	-2.947281	-0.767007
N	-2.947281	0.039385	0.767007
N	-0.018541	4.710200	1.799507
N	-4.710200	-0.018541	-1.799507
N	4.710200	0.018541	-1.799507
N	0.018541	-4.710200	1.799507
C	0.031292	2.731077	4.182382
H	0.976481	3.194077	4.488183
H	-0.792026	3.389912	4.475585
H	-0.068391	1.762126	4.682115
C	2.731077	-0.031292	-4.182382
H	3.389912	0.792026	-4.475585
H	3.194077	-0.976481	-4.488183
H	1.762126	0.068391	-4.682115
C	-2.731077	0.031292	-4.182382
H	-3.389912	-0.792026	-4.475585
H	-3.194077	0.976481	-4.488183
H	-1.762126	-0.068391	-4.682115
C	-0.031292	-2.731077	4.182382
H	-0.976481	-3.194077	4.488183
H	0.792026	-3.389912	4.475585
H	0.068391	-1.762126	4.682115
C	3.289003	1.220147	1.381545
H	3.254791	1.110179	2.468942
H	4.294314	1.577850	1.093739
H	2.556419	1.977627	1.105921
C	3.820073	-1.117121	1.156057
H	4.864297	-0.976619	0.830671
H	3.807122	-1.205584	2.246838
H	3.452739	-2.056512	0.741100
C	1.220147	-3.289003	-1.381545
H	1.577850	-4.294314	-1.093739
H	1.110179	-3.254791	-2.468942
H	1.977627	-2.556419	-1.105921
C	-1.117121	-3.820073	-1.156057
H	-1.205584	-3.807122	-2.246838
H	-0.976619	-4.864297	-0.830671
H	-2.056512	-3.452739	-0.741100
C	-1.220147	3.289003	-1.381545
H	-1.577850	4.294314	-1.093739
H	-1.110179	3.254791	-2.468942
H	-1.977627	2.556419	-1.105921
C	1.117121	3.820073	-1.156057
H	1.205584	3.807122	-2.246838
H	0.976619	4.864297	-0.830671
H	2.056512	3.452739	-0.741100
C	-3.820073	1.117121	1.156057
H	-4.864297	0.976619	0.830671
H	-3.807122	1.205584	2.246838
H	-3.452739	2.056512	0.741100
C	-3.289003	-1.220147	1.381545
H	-3.254791	-1.110179	2.468942
H	-4.294314	-1.577850	1.093739
H	-2.556419	-1.977627	1.105921
C	1.265908	-5.314224	2.208727
H	1.449436	-5.266226	3.295400
H	1.272747	-6.371216	1.920522
H	2.091782	-4.815461	1.702096
C	-5.349994	1.144101	-2.370798
H	-5.309741	1.180516	-3.471780
H	-6.405937	1.160309	-2.079700
H	-4.875807	2.045863	-1.982885
C	-5.314224	-1.265908	-2.208727
H	-6.371216	-1.272747	-1.920522
H	-5.266226	-1.449436	-3.295400
H	-4.815461	-2.091782	-1.702096
C	-1.265908	5.314224	2.208727
H	-1.449436	5.266226	3.295400
H	-1.272747	6.371216	1.920522
H	-2.091782	4.815461	1.702096
C	1.144101	5.349994	2.370798
H	1.160309	6.405937	2.079700
H	1.180516	5.309741	3.471780
H	2.045863	4.875807	1.982885
C	5.349994	-1.144101	-2.370798
H	5.309741	-1.180516	-3.471780
H	6.405937	-1.160309	-2.079700
H	4.875807	-2.045863	-1.982885
C	5.314224	1.265908	-2.208727
H	6.371216	1.272747	-1.920522
H	5.266226	1.449436	-3.295400
H	4.815461	2.091782	-1.702096
C	-1.144101	-5.349994	2.370798
H	-1.160309	-6.405937	2.079700
H	-1.180516	-5.309741	3.471780
H	-2.045863	-4.875807	1.982885

14b, neutral form
Stoichiometry: $C_{34}H_{61}BN_{16}$
Charge: 0
Multiplicity: 1
Point group: C_2
DLPNO-CCSD(T), Hartree: -2229.2694012
Gibbs in HMPA, Hartree: -2228.4190947
Gibbs in gas, Hartree: -2228.3967942
Nuclear coordinates, Å:

B	0.000000	0.000000	0.031233
N	1.178308	-0.388978	-0.937340
N	0.371520	1.154211	0.937300
N	-0.371520	-1.154211	0.937300
N	-1.178308	0.388978	-0.937340
C	0.000000	0.000000	-3.023477
C	0.000000	0.000000	3.113519
C	-1.113552	0.393362	-2.284756
C	-0.318920	-1.077217	2.319422
C	1.113552	-0.393362	-2.284756
C	0.318920	1.077217	2.319422
C	-2.430319	0.869744	-0.559182
C	2.430319	-0.869744	-0.559182
C	0.737409	2.441323	0.560579
C	-0.737409	-2.441323	0.560579
H	0.000000	0.000000	-4.099984
N	-0.673215	-2.336482	2.769561
N	2.290066	-0.853217	-2.770264
N	0.673215	2.336482	2.769561
N	-2.290066	0.853217	-2.770264
C	3.137455	-1.154100	-1.686369
C	-0.929839	-3.174552	1.686905
C	0.929839	3.174552	1.686905
C	-3.137455	1.154100	-1.686369
N	2.770982	-1.030459	0.778520
N	0.855520	2.804511	-0.791417
N	-2.770982	1.030459	0.778520
N	-0.855520	-2.804511	-0.791417
N	4.433562	-1.633486	-1.805287
N	-1.327165	-4.513151	1.768142
N	1.327165	4.513151	1.768142
N	-4.433562	1.633486	-1.805287
C	2.579073	-0.994532	-4.167098
H	1.928087	-1.740571	-4.632851
H	2.443354	-0.042612	-4.687748
H	3.611981	-1.308846	-4.287656
C	0.699732	2.651875	4.167945
H	1.664680	3.065424	4.470666
H	-0.089925	3.357347	4.443978
H	0.523583	1.702289	4.680418
C	-0.699732	-2.651875	4.167945
H	-1.664680	-3.065424	4.470666
H	0.089925	-3.357347	4.443978
H	-0.523583	-1.702289	4.680418
C	-2.579073	0.994532	-4.167098
H	-1.928087	1.740571	-4.632851
H	-2.443354	0.042612	-4.687748
H	-3.611981	1.308846	-4.287656
C	2.211279	2.942486	-1.278394
H	2.197973	3.006423	-2.370569
H	2.719793	3.838147	-0.888281
H	2.795208	2.064243	-1.001600
C	0.000000	3.890302	-1.217538
H	0.307853	4.867853	-0.818171
H	0.011201	3.947340	-2.310400
H	-1.024654	3.692316	-0.901107
C	-3.307713	2.313359	1.165294
H	-4.359893	2.454113	0.875817
H	-3.233719	2.407157	2.251669
H	-2.714005	3.112161	0.720009
C	-3.431533	-0.081958	1.427437
H	-3.322954	0.022475	2.508811
H	-4.504771	-0.134814	1.179457

H	-2.958080	-1.019199	1.140695
C	3.307713	-2.313359	1.165294
H	4.359893	-2.454113	0.875817
H	3.233719	-2.407157	2.251669
H	2.714005	-3.112161	0.720009
C	3.431533	0.081958	1.427437
H	3.322954	-0.022475	2.508811
H	4.504771	0.134814	1.179457
H	2.958080	1.019199	1.140695
C	0.000000	-3.890302	-1.217538
H	-0.307853	-4.867853	-0.818171
H	-0.011201	-3.947340	-2.310400
H	1.024654	-3.692316	-0.901107
C	-2.211279	-2.942486	-1.278394
H	-2.197973	-3.006423	-2.370569
H	-2.719793	-3.838147	-0.888281
H	-2.795208	-2.064243	-1.001600
C	-4.628882	2.989250	-2.267018
H	-4.624832	3.096898	-3.363433
H	-5.592336	3.361997	-1.907403
H	-3.845714	3.628855	-1.860621
C	-0.358117	-5.447615	2.301114
H	-0.278004	-5.426818	3.398592
H	-0.637656	-6.464450	2.009760
H	0.624916	-5.230998	1.882464
C	-2.676589	-4.761469	2.230662
H	-2.976399	-5.774202	1.945043
H	-2.795233	-4.676008	3.322031
H	-3.359742	-4.056664	1.756805
C	4.628882	-2.989250	-2.267018
H	4.624832	-3.096898	-3.363433
H	5.592336	-3.361997	-1.907403
H	3.845714	-3.628855	-1.860621
C	5.474411	-0.703629	-2.181245
H	6.442215	-1.082118	-1.839649
H	5.552197	-0.533292	-3.267408
H	5.295571	0.256699	-1.698580
C	0.358117	5.447615	2.301114
H	0.278004	5.426818	3.398592
H	0.637656	6.464450	2.009760
H	-0.624916	5.230998	1.882464
C	2.676589	4.761469	2.230662
H	2.976399	5.774202	1.945043
H	2.795233	4.676008	3.322031
H	3.359742	4.056664	1.756805
C	-5.474411	0.703629	-2.181245
H	-6.442215	1.082118	-1.839649
H	-5.552197	0.533292	-3.267408
H	-5.295571	-0.256699	-1.698580

14b, protonated form
Stoichiometry: $C_{34}H_{62}BN_{16}^{+}$
Charge: +1
Multiplicity: 1
Point group: D_{2d}
DLPNO-CCSD(T), Hartree: -2229.8054658
Gibbs in HMPA, Hartree: -2228.9619576
Gibbs in gas, Hartree: -2228.9191588
Nuclear coordinates, Å:

B	0.000000	0.000000	0.000000
N	0.000000	-1.230325	0.929992
N	-1.230325	0.000000	-0.929992
N	1.230325	0.000000	-0.929992
N	0.000000	1.230325	0.929992
C	0.000000	0.000000	3.021166
C	0.000000	0.000000	-3.021166
C	0.000000	1.178987	2.281510
C	1.178987	0.000000	-2.281510
C	0.000000	-1.178987	2.281510
C	-1.178987	0.000000	-2.281510
C	0.000000	2.569614	0.541788
C	0.000000	-2.569614	0.541788
C	-2.569614	0.000000	-0.541788
C	2.569614	0.000000	-0.541788
H	0.000000	0.000000	-4.097467
H	0.000000	0.000000	4.097467
N	2.446800	0.000000	-2.752493
N	0.000000	-2.446800	2.752493
N	-2.446800	0.000000	-2.752493
N	0.000000	2.446800	2.752493
C	0.000000	-3.337136	1.662616
C	3.337136	0.000000	-1.662616
C	-3.337136	0.000000	-1.662616
C	0.000000	3.337136	1.662616
N	0.000000	-2.928319	-0.809233
N	-2.928319	0.000000	0.809233
N	0.000000	2.928319	-0.809233
N	2.928319	0.000000	0.809233
N	0.000000	-4.720153	1.758309
N	4.720153	0.000000	-1.758309
N	-4.720153	0.000000	-1.758309
N	0.000000	4.720153	1.758309
C	0.000000	-2.778248	4.151435
H	0.888092	-2.376265	4.645286
H	-0.888092	-2.376265	4.645286
H	0.000000	-3.858083	4.262412
C	-2.778248	0.000000	-4.151435
H	-2.376265	0.888092	-4.645286
H	-2.376265	-0.888092	-4.645286
H	-3.858083	0.000000	-4.262412
C	2.778248	0.000000	-4.151435
H	2.376265	-0.888092	-4.645286
H	2.376265	0.888092	-4.645286
H	3.858083	0.000000	-4.262412
C	0.000000	2.778248	4.151435
H	-0.888092	2.376265	4.645286
H	0.888092	2.376265	4.645286
H	0.000000	3.858083	4.262412
C	-3.584158	-1.207503	1.273423
H	-3.635048	-1.185635	2.364488
H	-4.605855	-1.320022	0.884430
H	-2.999033	-2.080561	0.983092
C	-3.584158	1.207503	1.273423
H	-4.605855	1.320022	0.884430
H	-3.635048	1.185635	2.364488
H	-2.999033	2.080561	0.983092
C	-1.207503	3.584158	-1.273423
H	-1.320022	4.605855	-0.884430
H	-1.185635	3.635048	-2.364488
H	-2.080561	2.999033	-0.983092
C	1.207503	3.584158	-1.273423
H	1.185635	3.635048	-2.364488
H	1.320022	4.605855	-0.884430
H	2.080561	2.999033	-0.983092
C	1.207503	-3.584158	-1.273423
H	1.320022	-4.605855	-0.884430
H	1.185635	-3.635048	-2.364488
H	2.080561	-2.999033	-0.983092
C	-1.207503	-3.584158	-1.273423
H	-1.185635	-3.635048	-2.364488
H	-1.320022	-4.605855	-0.884430
H	-2.080561	-2.999033	-0.983092
C	3.584158	-1.207503	1.273423
H	4.605855	-1.320022	0.884430
H	3.635048	-1.185635	2.364488
H	2.999033	-2.080561	0.983092
C	3.584158	1.207503	1.273423
H	3.635048	1.185635	2.364488
H	4.605855	1.320022	0.884430
H	2.999033	2.080561	0.983092
C	-1.220980	5.369513	2.192216
H	-1.361462	5.376687	3.283289
H	-1.214409	6.409061	1.856004
H	-2.079966	4.873036	1.741078
C	5.369513	-1.220980	-2.192216
H	5.376687	-1.361462	-3.283289
H	6.409061	-1.214409	-1.856004
H	4.873036	-2.079966	-1.741078
C	5.369513	1.220980	-2.192216
H	6.409061	1.214409	-1.856004
H	5.376687	1.361462	-3.283289
H	4.873036	2.079966	-1.741078
C	1.220980	-5.369513	2.192216
H	1.361462	-5.376687	3.283289
H	1.214409	-6.409061	1.856004
H	2.079966	-4.873036	1.741078
C	-1.220980	-5.369513	2.192216
H	-1.214409	-6.409061	1.856004
H	-1.361462	-5.376687	3.283289
H	-2.079966	-4.873036	1.741078
C	-5.369513	1.220980	-2.192216
H	-5.376687	1.361462	-3.283289
H	-6.409061	1.214409	-1.856004
H	-4.873036	2.079966	-1.741078
C	-5.369513	-1.220980	-2.192216
H	-6.409061	-1.214409	-1.856004
H	-5.376687	-1.361462	-3.283289
H	-4.873036	-2.079966	-1.741078
C	1.220980	5.369513	2.192216
H	1.214409	6.409061	1.856004
H	1.361462	5.376687	3.283289
H	2.079966	4.873036	1.741078

14b, diprotonated form

Stoichiometry: $C_{34}H_{63}BN_{16}^{2+}$
Charge: +2
Multiplicity: 1
Point group: C_2
DLPNO-CCSD(T), Hartree: -2230.1509143
Gibbs in HMPA, Hartree: -2229.4195325
Nuclear coordinates, Å:

B	0.000000	0.000000	-0.035054
N	1.226479	-0.020855	-0.917488
N	0.018733	1.251152	0.923340
N	-0.018733	-1.251152	0.923340
N	-1.226479	0.020855	-0.917488
C	0.000000	0.000000	-3.011590
C	0.000000	0.000000	3.078529
C	-1.178650	0.029815	-2.274994
C	-0.002803	-1.224517	2.246545
C	1.178650	-0.029815	-2.274994
C	0.002803	1.224517	2.246545
C	-2.568472	0.060840	-0.524021
C	2.568472	-0.060840	-0.524021
C	0.000000	2.580780	0.516164
C	0.000000	-2.580780	0.516164
H	0.000000	0.000000	-4.087940
N	0.021088	-2.467950	2.724922
N	2.446302	-0.068746	-2.736454
N	-0.021088	2.467950	2.724922
N	-2.446302	0.068746	-2.736454
C	3.334683	-0.089352	-1.644636
C	0.021233	-3.359632	1.643283
C	-0.021233	3.359632	1.643283
C	-3.334683	0.089352	-1.644636
N	2.921699	-0.063775	0.834420
N	-0.019147	2.926167	-0.828600
N	-2.921699	0.063775	0.834420
N	0.019147	-2.926167	-0.828600
N	4.715422	-0.129244	-1.732231
N	0.036370	-4.732089	1.744669
N	-0.036370	4.732089	1.744669
N	-4.715422	0.129244	-1.732231
C	2.785553	-0.089154	-4.137903
H	2.371021	-0.975647	-4.622257
H	2.401915	0.801532	-4.639560
H	3.865214	-0.108241	-4.242730
C	-0.056822	2.837733	4.125787
H	-1.066305	2.745298	4.528779
H	0.623820	2.210189	4.699452
H	0.266576	3.869695	4.220487
C	0.056822	-2.837733	4.125787
H	1.066305	-2.745298	4.528779
H	-0.623820	-2.210189	4.699452
H	-0.266576	-3.869695	4.220487
C	-2.785553	0.089154	-4.137903
H	-2.371021	0.975647	-4.622257
H	-2.401915	-0.801532	-4.639560
H	-3.865214	0.108241	-4.242730
C	1.255804	3.364793	-1.376820
H	1.179290	3.389690	-2.464787
H	1.543448	4.366416	-1.030558
H	2.042518	2.659240	-1.110279
C	-1.125407	3.781391	-1.233178
H	-1.028441	4.811187	-0.870170
H	-1.163701	3.803549	-2.323361
H	-2.066162	3.365016	-0.872790
C	-3.619613	1.269943	1.262460
H	-4.624035	1.355312	0.831523
H	-3.719776	1.258551	2.350476
H	-3.041994	2.150768	0.980330
C	-3.621786	-1.139776	1.267275
H	-3.725234	-1.122959	2.354804
H	-4.625445	-1.225584	0.833984
H	-3.046123	-2.024236	0.992313
C	3.619613	-1.269943	1.262460
H	4.624035	-1.355312	0.831523
H	3.719776	-1.258551	2.350476
H	3.041994	-2.150768	0.980330
C	3.621786	1.139776	1.267275
H	3.725234	1.122959	2.354804
H	4.625445	1.225584	0.833984
H	3.046123	2.024236	0.992313
C	1.125407	-3.781391	-1.233178
H	1.028441	-4.811187	-0.870170
H	1.163701	-3.803549	-2.323361
H	2.066162	-3.365016	-0.872790
C	-1.255804	-3.364793	-1.376820
H	-1.179290	-3.389690	-2.464787
H	-1.543448	-4.366416	-1.030558
H	-2.042518	-2.659240	-1.110279
C	-5.327993	1.371003	-2.173611
H	-5.328836	1.505019	-3.263824
H	-6.366926	1.393822	-1.839140
H	-4.808874	2.217931	-1.724874
C	1.234834	-5.376554	2.252029
H	1.280577	-5.428202	3.348078
H	1.276082	-6.399352	1.873334
H	2.118919	-4.850271	1.891783
C	-1.211900	-5.421203	2.024318
H	-1.149329	-6.441903	1.642877
H	-1.451633	-5.481783	3.094907
H	-2.034144	-4.919562	1.515057
C	5.327993	-1.371003	-2.173611
H	5.328836	-1.505019	-3.263824
H	6.366926	-1.393822	-1.839140
H	4.808874	-2.217931	-1.724874
C	5.399322	1.074174	-2.175037
H	6.438487	1.035449	-1.842821
H	5.405597	1.207798	-3.265346
H	4.932478	1.950410	-1.725152
C	-1.234834	5.376554	2.252029
H	-1.280577	5.428202	3.348078
H	-1.276082	6.399352	1.873334
H	-2.118919	4.850271	1.891783
C	1.211900	5.421203	2.024318
H	1.149329	6.441903	1.642877
H	1.451633	5.481783	3.094907
H	2.034144	4.919562	1.515057
C	-5.399322	-1.074174	-2.175037
H	-6.438487	-1.035449	-1.842821
H	-5.405597	-1.207798	-3.265346
H	-4.932478	-1.950410	-1.725152
H	-0.877486	0.004831	3.736238
H	0.877486	-0.004831	3.736238

14b, triprotonated form
Stoichiometry: $C_{34}H_{64}BN_{16}^{3+}$
Charge: +3
Multiplicity: 1
Point group: D_2
DLPNO-CCSD(T), Hartree: -2230.3992763
Gibbs in HMPA, Hartree: -2229.8694232
Nuclear coordinates, Å:

B	0.000000	0.000000	0.000000
N	0.881760	0.883047	0.908927
N	0.881760	-0.883047	-0.908927
N	-0.881760	0.883047	-0.908927
N	-0.881760	-0.883047	0.908927
C	0.000000	0.000000	3.069829
C	0.000000	0.000000	-3.069829
C	-0.867902	-0.864705	2.238113
C	-0.867902	0.864705	-2.238113
C	0.867902	0.864705	2.238113
C	0.867902	-0.864705	-2.238113
C	-1.829278	-1.816625	0.496682
C	1.829278	1.816625	0.496682
C	1.829278	-1.816625	-0.496682
C	-1.829278	1.816625	-0.496682
N	-1.752340	1.738844	-2.706247
N	1.752340	1.738844	2.706247
N	1.752340	-1.738844	-2.706247
N	-1.752340	-1.738844	2.706247
C	2.387162	2.365659	1.620328
C	-2.387162	2.365659	-1.620328
C	2.387162	-2.365659	-1.620328
C	-2.387162	-2.365659	1.620328
N	2.072684	2.050070	-0.858246
N	2.072684	-2.050070	0.858246
N	-2.072684	-2.050070	-0.858246
N	-2.072684	2.050070	0.858246
N	3.365316	3.326390	1.708677
N	-3.365316	3.326390	-1.708677
N	3.365316	-3.326390	-1.708677
N	-3.365316	-3.326390	1.708677
C	2.031221	1.989414	4.110848
H	1.102072	2.124237	4.663146
H	2.601956	1.167186	4.543486
H	2.613979	2.901128	4.189435
C	2.031221	-1.989414	-4.110848
H	1.102072	-2.124237	-4.663146
H	2.601956	-1.167186	-4.543486
H	2.613979	-2.901128	-4.189435
C	-2.031221	1.989414	-4.110848
H	-1.102072	2.124237	-4.663146
H	-2.601956	1.167186	-4.543486
H	-2.613979	2.901128	-4.189435
C	-2.031221	-1.989414	4.110848

H	-1.102072	-2.124237	4.663146
H	-2.601956	-1.167186	4.543486
H	-2.613979	-2.901128	4.189435
C	3.434108	-1.716101	1.277884
H	3.496432	-1.796492	2.364233
H	4.185879	-2.384619	0.845606
H	3.669190	-0.689373	0.996097
C	1.686705	-3.387798	1.306711
H	2.323519	-4.174310	0.887768
H	1.769898	-3.433014	2.393747
H	0.650444	-3.589153	1.033372
C	-1.686705	-3.387798	-1.306711
H	-2.323519	-4.174310	-0.887768
H	-1.769898	-3.433014	-2.393747
H	-0.650444	-3.589153	-1.033372
C	-3.434108	-1.716101	-1.277884
H	-3.496432	-1.796492	-2.364233
H	-4.185879	-2.384619	-0.845606
H	-3.669190	-0.689373	-0.996097
C	1.686705	3.387798	-1.306711
H	2.323519	4.174310	-0.887768
H	1.769898	3.433014	-2.393747
H	0.650444	3.589153	-1.033372
C	3.434108	1.716101	-1.277884
H	3.496432	1.796492	-2.364233
H	4.185879	2.384619	-0.845606
H	3.669190	0.689373	-0.996097
C	-1.686705	3.387798	1.306711
H	-2.323519	4.174310	0.887768
H	-1.769898	3.433014	2.393747
H	-0.650444	3.589153	1.033372
C	-3.434108	1.716101	1.277884
H	-3.496432	1.796492	2.364233
H	-4.185879	2.384619	0.845606
H	-3.669190	0.689373	0.996097
C	-2.972410	-4.687016	2.053425
H	-2.885350	-4.859539	3.133496
H	-3.723265	-5.378949	1.669549
H	-2.018359	-4.929622	1.585876
C	-2.972410	4.687016	-2.053425
H	-2.885350	4.859539	-3.133496
H	-3.723265	5.378949	-1.669549
H	-2.018359	4.929622	-1.585876
C	-4.684643	2.923289	-2.178007
H	-5.422040	3.638046	-1.810361
H	-4.772057	2.889221	-3.270936
H	-4.938601	1.941951	-1.776879
C	2.972410	4.687016	2.053425
H	2.885350	4.859539	3.133496
H	3.723265	5.378949	1.669549
H	2.018359	4.929622	1.585876
C	4.684643	2.923289	2.178007
H	5.422040	3.638046	1.810361
H	4.772057	2.889221	3.270936
H	4.938601	1.941951	1.776879
C	2.972410	-4.687016	-2.053425
H	2.885350	-4.859539	-3.133496
H	3.723265	-5.378949	-1.669549
H	2.018359	-4.929622	-1.585876
C	4.684643	-2.923289	-2.178007
H	5.422040	-3.638046	-1.810361
H	4.772057	-2.889221	-3.270936
H	4.938601	-1.941951	-1.776879
C	-4.684643	-2.923289	2.178007
H	-5.422040	-3.638046	1.810361
H	-4.772057	-2.889221	3.270936
H	-4.938601	-1.941951	1.776879
H	0.619000	-0.619792	3.729682
H	0.619000	0.619792	-3.729682
H	-0.619000	-0.619792	-3.729682
H	-0.619000	0.619792	3.729682

14b, deprotonated form, tautomer
Stoichiometry: $C_{34}H_{60}BN_{16}^{-}$
Charge: −1
Multiplicity: 1
Point group: C_1
DLPNO-CCSD(T), Hartree: -2228.6427076
Gibbs in HMPA, Hartree: -2227.8680290
Nuclear coordinates, Å:

B	-0.010730	-0.017240	0.002736
N	0.846749	0.855886	-0.961161
N	-0.853246	0.845453	0.949460
N	0.868343	-0.880286	0.914781
N	-0.906431	-0.881207	-0.922552
C	-0.097406	0.019149	-3.036600
C	0.053444	-0.012218	3.105512
C	-0.931262	-0.837169	-2.285379
C	0.838697	-0.802031	2.294675
C	0.758239	0.822022	-2.322210
C	-0.767929	0.772484	2.326286
C	-1.853040	-1.799287	-0.521568
C	1.840903	1.760354	-0.611816
C	-1.793446	1.802890	0.593532
C	1.850850	-1.779475	0.525341
H	-0.170666	0.061467	-4.109240
N	1.803226	-1.691460	2.736173
N	1.679644	1.700530	-2.821292
N	-1.680096	1.699358	2.801966
N	-1.854288	-1.694639	-2.758501
C	2.364350	2.295683	-1.744717
C	2.430440	-2.290464	1.643513
C	-2.311930	2.334099	1.732050
C	-2.440352	-2.308070	-1.652193
N	2.177628	1.986539	0.722871
N	-2.062170	2.102786	-0.747776
N	-2.102232	-2.056570	0.830140
N	2.124949	-2.028942	-0.825622
N	3.363283	3.257628	-1.872462
N	3.430098	-3.266663	1.721849
N	-3.318056	3.299874	1.844847
N	-3.473919	-3.248054	-1.712661
C	1.879428	1.899370	-4.219674
H	2.165575	0.962411	-4.712965
H	0.962412	2.260678	-4.699293
H	2.665734	2.633882	-4.376090
C	-1.849749	1.932861	4.202649
H	-1.502370	2.928112	4.502423
H	-2.893415	1.818641	4.510580
H	-1.229287	1.177111	4.694214
C	2.105552	-1.838386	4.125989
H	2.080984	-2.886550	4.438019
H	3.084421	-1.417827	4.384896
H	1.329499	-1.270693	4.648489
C	-2.259841	-1.831363	-4.129050
H	-1.402033	-1.586294	-4.761513
H	-2.523212	-2.877069	-4.305630
C	-1.961214	3.491324	-1.122185
H	-1.930930	3.561457	-2.214030
H	-2.802076	4.105180	-0.761132
H	-1.033911	3.913328	-0.731284
C	-3.207809	1.441974	-1.336203
H	-4.162783	1.906354	-1.034594
H	-3.132698	1.473056	-2.426281
H	-3.222911	0.393361	-1.044471
C	-3.448031	-1.809045	1.288560
H	-4.171353	-2.565153	0.944031
H	-3.451606	-1.793349	2.382709
H	-3.779000	-0.829820	0.940552
C	-1.532284	-3.266515	1.372604
H	-1.560401	-3.216655	2.464765
H	-2.064463	-4.177877	1.048247
H	-0.486911	-3.348727	1.074937
C	3.563804	1.836433	1.081070
H	4.193998	2.686450	0.770963
H	3.637053	1.732889	2.167809
H	3.962916	0.926598	0.631440
C	1.531025	3.098565	1.378501
H	1.584455	2.956585	2.460929
H	1.992985	4.068897	1.122357
H	0.477649	3.129547	1.104046
C	3.508382	-1.945034	-1.221597
H	4.113507	-2.804471	-0.892069
H	3.560858	-1.887259	-2.313561
H	3.952453	-1.035996	-0.813633
C	1.437930	-3.153897	-1.421667
H	1.484890	-3.072791	-2.510865
H	1.875467	-4.122689	-1.123369
H	0.386545	-3.143426	-1.139245
C	-4.712585	-2.752882	-2.290378
H	-4.597517	-2.423867	-3.332119
H	-5.469379	-3.540721	-2.231976
H	-5.064603	-1.901749	-1.702605
C	4.702258	-2.865417	2.277198
H	4.715354	-2.821990	3.378319
H	5.476836	-3.575091	1.966878
H	4.969688	-1.879969	1.895081
C	3.015342	-4.589072	2.132648
H	3.775354	-5.319600	1.834795
H	2.866274	-4.690920	3.220637
H	2.080169	-4.845836	1.635840
C	4.649869	2.840845	-2.379208
H	4.705958	2.783153	-3.479248
H	5.420249	3.547094	-2.051556

H 4.895192 1.858176 -1.977078
C 2.968579 4.589456 -2.267081
H 3.733569 5.307630 -1.952880
H 2.827812 4.713509 -3.354239
H 2.032455 4.848922 -1.773505
C -4.623551 2.831561 2.252285
H -4.713652 2.652927 3.336833
H -5.380452 3.573736 1.976585
H -4.851957 1.900903 1.733771
C -2.953291 4.569238 2.430120
H -3.692853 5.326742 2.149114
H -2.897003 4.554456 3.530772
H -1.982685 4.881275 2.043721
C -3.101604 -4.575418 -2.154452
H -3.912504 -5.272616 -1.919055
H -2.890276 -4.650133 -3.231499
H -2.210753 -4.896207 -1.612158

14b, neutral form, tautomer 1
Stoichiometry: $C_{34}H_{61}BN_{16}$
Charge: 0
Multiplicity: 1
Point group: C_1
DLPNO-CCSD(T), Hartree: -2229.2584880
Gibbs in HMPA, Hartree: -2228.4128588
Nuclear coordinates, Å:
B -0.033402 -0.017795 -0.030370
N 0.162767 -1.236480 -0.919821
N 1.145269 0.134237 0.982988
N -1.308643 -0.166906 0.857666
N -0.130966 1.198927 -0.946867
C 0.186714 -0.006994 -3.027321
C -0.185337 -0.029927 3.004738
C -0.008247 1.144726 -2.309212
C -1.318525 -0.166724 2.207567
C 0.269506 -1.203231 -2.284139
C 1.021662 0.123378 2.326276
C -0.289721 2.530910 -0.576029
C 0.329424 -2.546134 -0.522508
C 2.488561 0.329258 0.666212
C -2.621052 -0.300021 0.409211
H -0.241668 -0.018145 4.079662
H 0.336002 -0.007323 -4.092906
N -2.599313 -0.299569 2.624803
N 0.496645 -2.443436 -2.756014
N 2.247677 0.304122 2.869356
N -0.105628 2.424304 -2.781258
C 0.532170 -3.298067 -1.651244
C -3.435140 -0.389503 1.494953
C 3.188005 0.432161 1.828107
C -0.275518 3.297956 -1.693455
N 0.286155 -2.918598 0.830506
N 2.912911 0.412305 -0.656538
N -0.431742 2.894862 0.770307
N -2.925133 -0.300502 -0.949821
N 0.778191 -4.671103 -1.706024
N -4.811417 -0.556300 1.539933
N 4.551513 0.605671 2.011245
N -0.408216 4.680570 -1.787489
C 0.807228 -2.777746 -4.099805
H 0.317074 -2.075285 -4.773726
H 0.481841 -3.793191 -4.313747
C 2.500149 0.344873 4.280132
H 2.033808 1.219824 4.743146
H 2.110925 -0.554027 4.765390
H 3.572240 0.390819 4.449362
C -2.990602 -0.338220 4.003563
H -2.442918 -1.120600 4.535639
H -2.798510 0.618552 4.498880
H -4.052844 -0.556859 4.067544
C 0.000651 2.742894 -4.171437
H 0.989254 2.478837 -4.563075
H -0.748003 2.191441 -4.749517
H -0.160799 3.807371 -4.316724
C 3.391444 -0.819310 -1.258334
H 3.356948 -0.733010 -2.346267
H 4.423483 -1.056080 -0.953914
H 2.745261 -1.649472 -0.979553
C 3.722192 1.559062 -0.998765
H 4.755560 1.491831 -0.627987
H 3.755431 1.648477 -2.087448
H 3.267273 2.467161 -0.600579
C 0.650190 3.687808 1.312722
H 0.666403 4.719068 0.928917
H 0.552196 3.730180 2.401325
H 1.603734 3.212831 1.079617
C -1.737973 3.395508 1.139462
H -1.809982 3.441485 2.229936
H -1.946010 4.399726 0.739427
H -2.506733 2.710381 0.780956
C -0.857376 -3.725143 1.206854
H -0.819779 -4.743958 0.793187
H -0.901879 -3.800514 2.297648
H -1.775126 -3.245239 0.864727
C 1.527400 -3.456730 1.352235
H 1.448539 -3.549891 2.439633
H 1.774606 -4.443740 0.936030
H 2.346020 -2.770741 1.130888
C -3.030647 -1.601940 -1.581290
H -3.976778 -2.110827 -1.336186
H -2.971797 -1.480455 -2.664659
H -2.199340 -2.235832 -1.277848
C -3.957734 0.617514 -1.373442
H -3.910930 0.716787 -2.460758
H -4.972270 0.292237 -1.100582
H -3.779274 1.602002 -0.939270
C 0.767609 5.440644 -2.155056
H 0.977446 5.449851 -3.235892
H 0.641988 6.479516 -1.836861
H 1.638873 5.030562 -1.644645
C -5.328986 -1.867321 1.864605
H -5.375588 -2.074892 2.945699
H -6.343528 -1.966349 1.468214
H -4.706041 -2.630131 1.398616
C -5.624457 0.550466 1.991832
H -6.636546 0.446401 1.590383
H -5.712727 0.626921 3.087120
H -5.207207 1.485714 1.618697
C -0.290479 -5.490765 -2.244675
H -0.417041 -5.407104 -3.332601
H -0.089936 -6.539061 -2.006167
H -1.233054 -5.209999 -1.772043
C 2.103866 -5.033671 -2.184900
H 2.222409 -6.117049 -2.104729
H 2.289523 -4.720080 -3.219810
H 2.856019 -4.563060 -1.547853
C 5.022581 1.872352 2.523794
H 4.979823 1.959076 3.621150
H 6.064558 2.020401 2.226183
H 4.430799 2.681618 2.095988
C 5.340378 -0.543658 2.396390
H 6.381425 -0.386369 2.100187
H 5.332746 -0.749355 3.478889
H 4.970341 -1.428377 1.879360
C -1.645639 5.198376 -2.331247
H -1.776454 6.236161 -2.011378
H -1.694470 5.184326 -3.431313
H -2.482590 4.615000 -1.948070

14b, neutral form, tautomer 2
Stoichiometry: $C_{34}H_{61}BN_{16}$
Charge: 0
Multiplicity: 1
Point group: C_1
DLPNO-CCSD(T), Hartree: -2229.2371413
Gibbs in HMPA, Hartree: -2228.3981428
Nuclear coordinates, Å:
B -0.029393 -0.029181 0.063877
N 0.918414 0.627658 -0.985480
N -0.825318 1.039020 0.825508
N 0.760228 -0.815749 1.097531
N -0.973223 -0.950834 -0.763535
C -0.003128 -0.475469 -2.938446
C 0.032164 0.472071 3.018372
C -0.921495 -1.106885 -2.104487
C 0.813317 -0.526480 2.408196
C 0.901313 0.378920 -2.311089
C -0.768038 1.210338 2.173886
C -2.021938 -1.725803 -0.273021
C 1.947667 1.529074 -0.720239
C -1.729309 1.961540 0.305831
C 1.668529 -1.873375 0.837556
H 0.044140 0.630056 4.082628
H 0.004386 -0.633963 -4.003088
N 1.704164 -1.345888 2.991712
N 1.886234 1.100085 -2.893019
N -1.625624 2.214449 2.503252
N -1.906104 -1.957884 -2.472711
C 2.559713 1.831438 -1.895717
C 2.273532 -2.194967 2.033915
C -2.230229 2.698018 1.329149
C -2.607345 -2.363984 -1.321341
N 2.198123 1.984338 0.573737
N -1.993909 2.019722 -1.067231

N	-2.335398	-1.744775	1.085045
N	1.890205	-2.293310	-0.473408
N	3.643050	2.666913	-2.120229
N	3.209707	-3.107640	2.407127
N	-3.152023	3.737336	1.273997
N	-3.665912	-3.259852	-1.314653
C	2.179539	1.067869	-4.297004
H	2.365991	0.042461	-4.626636
H	1.352920	1.478478	-4.884120
H	3.070246	1.659341	-4.488153
C	-1.819809	2.667645	3.848158
H	-2.223643	1.868112	4.477073
H	-0.873386	3.000759	4.284725
H	-2.516790	3.500723	3.852987
C	2.093919	-1.344713	4.377321
H	1.895114	-0.364391	4.810129
H	1.555978	-2.113293	4.937669
H	3.184583	-1.557530	4.351312
C	-2.140319	-2.370454	-3.826197
H	-2.449703	-1.526364	-4.449550
H	-1.235523	-2.807719	-4.256633
H	-2.925768	-3.120528	-3.839799
C	-1.360745	3.115487	-1.770917
H	-1.432388	2.939811	-2.847580
H	-1.821601	4.090808	-1.549813
H	-0.303802	3.161063	-1.508168
C	-3.377516	1.831819	-1.445807
H	-4.017299	2.690930	-1.197083
H	-3.431021	1.666874	-2.525544
H	-3.774683	0.945507	-0.950203
C	-3.726662	-1.532560	1.417385
H	-4.365550	-2.400784	1.202026
H	-3.800792	-1.310329	2.484993
H	-4.111513	-0.673032	0.867281
C	-1.731088	-2.816478	1.855852
H	-1.806965	-2.578656	2.918952
H	-2.223346	-3.785949	1.680989
H	-0.673909	-2.908838	1.610763
C	3.343538	1.388550	1.243123
H	4.301289	1.754342	0.844076
H	3.300558	1.632322	2.305909
H	3.320559	0.302638	1.161855
C	2.125330	3.418119	0.751258
H	2.101593	3.637235	1.821579
H	2.977594	3.956336	0.310664
H	1.203780	3.801393	0.310097
C	3.200988	-2.067003	-1.040165
H	3.942578	-2.823421	-0.746249
H	3.129474	-2.071057	-2.134378
H	3.575333	-1.094280	-0.723205
C	1.258752	-3.502357	-0.950286
H	1.169526	-3.459946	-2.041618
H	1.812684	-4.417026	-0.694913
H	0.254841	-3.584686	-0.533717
C	-4.928751	-2.837347	-1.877877
H	-4.994190	-2.949096	-2.971595
H	-5.738005	-3.429717	-1.441693
H	-5.105645	-1.790171	-1.632664
C	4.432416	-2.492810	2.930352
H	4.904490	-3.208711	3.618040
H	5.128640	-2.319222	2.088684
C	3.266133	-4.369379	1.714228
H	3.817730	-4.326022	0.760385
H	3.803933	-5.072126	2.354792
H	2.263895	-4.772359	1.522164
C	4.934497	2.058951	-2.360628
H	5.095259	1.751160	-3.405967
H	5.723634	2.770407	-2.102839
H	5.048800	1.181558	-1.725211
C	3.414656	3.915957	-2.810900
H	4.212708	4.620158	-2.560013
H	3.389596	3.826576	-3.908584
H	2.468430	4.346797	-2.483194
C	-4.477404	3.491391	1.801003
H	-4.551390	3.587687	2.895300
H	-5.180507	4.204887	1.362103
H	-4.797041	2.486116	1.526921
C	-2.662133	5.083008	1.484607
H	-3.363582	5.797356	1.044393
H	-2.536880	5.354518	2.544434
H	-1.699032	5.201143	0.988635
C	-3.368347	-4.667545	-1.468740
H	-4.179998	-5.259987	-1.037223
H	-3.247635	-4.986405	-2.516168
H	-2.450523	-4.906675	-0.932823

14b, neutral form, tautomer 3

Stoichiometry: $C_{34}H_{61}BN_{16}$
Charge: 0
Multiplicity: 1
Point group: C_1
DLPNO-CCSD(T), Hartree: -2229.2378996
Gibbs in HMPA, Hartree: -2228.3945804
Nuclear coordinates, Å:

B	0.058131	-0.061004	-0.011229
N	-0.758871	-0.968023	-0.941572
N	1.084927	-0.887081	0.803589
N	-0.833757	0.650964	1.021002
N	0.776433	0.952046	-0.933712
C	-0.178250	0.150183	-3.013888
C	0.299810	-0.209400	2.993335
C	0.622956	1.008428	-2.277855
C	-0.679447	0.567760	2.359355
C	-0.892206	-0.792394	-2.271206
C	1.146532	-0.915826	2.156668
C	1.632338	1.977977	-0.549491
C	-1.649690	-1.956882	-0.541133
C	2.105121	-1.689619	0.302031
C	-1.889721	1.530430	0.738043
H	0.402726	-0.234946	4.064662
H	-0.332387	0.277885	-4.071403
N	-1.589667	1.365392	2.941326
N	-1.808378	-1.669771	-2.725656
N	2.174321	-1.727560	2.514797
N	1.374635	2.035332	-2.745996
C	-2.307149	-2.404203	-1.637874
C	-2.350098	2.004598	1.930903
C	2.787690	-2.221961	1.348194
C	2.008395	2.663598	-1.659006
N	-1.803577	-2.288084	0.805706
N	2.303741	-1.833017	-1.073400
N	1.975953	2.169729	0.791837
N	-2.302376	1.772434	-0.557744
N	-3.391590	-3.263533	-1.693247
N	-3.444370	2.806201	2.183246
N	3.867934	-3.094862	1.324603
N	2.836946	3.774723	-1.751074
C	-2.306076	-1.676154	-4.070741
H	-2.748941	-0.707617	-4.317961
H	-1.506655	-1.896013	-4.783612
H	-3.071638	-2.441390	-4.160277
C	2.535127	-1.991383	3.875888
H	2.895381	-1.085693	4.374284
H	1.674470	-2.371868	4.433189
H	3.322088	-2.739788	3.901783
C	-1.752828	1.525271	4.355432
H	-1.733966	0.551845	4.850257
H	-0.970315	2.154987	4.791653
H	-2.718911	1.987499	4.544897
C	1.417756	2.415830	-4.127289
H	1.782607	1.590655	-4.745796
H	0.424028	2.702776	-4.483417
H	2.087685	3.262448	-4.246490
C	1.798985	-3.060427	-1.653280
H	1.781688	-2.960192	-2.741266
H	2.413234	-3.938030	-1.396796
H	0.776727	-3.236111	-1.319370
C	3.618309	-1.474767	-1.557445
H	4.396310	-2.205086	-1.290463
H	3.583361	-1.395057	-2.647200
H	3.902882	-0.500015	-1.159550
C	3.389357	2.105018	1.089421
H	3.951219	2.979524	0.728976
H	3.520485	2.038318	2.172925
H	3.818045	1.206417	0.643836
C	1.308355	3.278329	1.444675
H	1.439869	3.187298	2.526107
H	1.702993	4.257013	1.130193
H	0.240202	3.244637	1.229980
C	-3.089242	-1.917720	1.374259
H	-3.855011	-2.689418	1.203443
H	-2.979638	-1.774817	2.454371
H	-3.416173	-0.984208	0.897779
C	-1.359461	-3.608604	1.181480
H	-1.332649	-3.678448	2.272741
H	-2.017278	-4.408903	0.806135
H	-0.348712	-3.786722	0.808641
C	-3.414353	0.877583	-0.941754
H	-4.365477	1.407079	-0.727753
H	-3.372875	0.765935	-2.039400
C	-2.429617	3.171445	-0.910380
H	-2.518741	3.235312	-1.997750
H	-3.324440	3.645499	-0.476814
H	-1.546299	3.749159	-0.602455

C 4.146078 3.599699 -2.340071
H 4.150115 3.612708 -3.441546
H 4.806501 4.403719 -2.002845
H 4.570118 2.651131 -2.010685
C -4.739003 2.184377 2.331898
H -4.961372 1.869890 3.365915
H -5.520872 2.886196 2.024702
H -4.789694 1.314710 1.676642
C -3.277231 4.102481 2.783499
H -4.060831 4.780274 2.428494
H -3.325672 4.102571 3.885774
H -2.314073 4.520075 2.487902
C -4.697725 -2.640092 -1.800910
H -5.036975 -2.532504 -2.843431
H -5.442173 -3.243507 -1.271659
H -4.646830 -1.645509 -1.348604
C -3.250581 -4.546335 -2.333653
H -3.996537 -5.239048 -1.931932
H -3.387706 -4.522913 -3.428069
H -2.261467 -4.955467 -2.124314
C 5.159121 -2.589470 1.736715
H 5.309567 -2.574501 2.827606
H 5.947567 -3.214151 1.307439
H 5.289065 -1.574146 1.362284
C 3.624907 -4.480692 1.662567
H 4.413826 -5.103514 1.231402
H 3.602016 -4.680854 2.745574
H 2.671869 -4.796413 1.239216
C 2.218056 5.055273 -2.021297
H 2.870553 5.857099 -1.663921
H 2.022357 5.240176 -3.089150
H 1.270665 5.120696 -1.487197

14b, neutral form, tautomer 2, transition state
Stoichiometry: $C_{34}H_{61}BN_{16}$
Charge: 0
Multiplicity: 1
Point group: C_1
DLPNO-CCSD(T), Hartree: -2229.2029882
Gibbs in HMPA, Hartree: -2228.3573643
Nuclear coordinates, Å:
B -0.048994 -0.027683 0.048482
N 0.884130 0.664121 -0.987257
N -0.864671 1.006031 0.839627
N 0.754836 -0.825343 1.062962
N -0.981261 -0.947666 -0.796692
C -0.018362 -0.416986 -2.961553
C -0.062753 0.341947 3.027480
C -0.918661 -1.087549 -2.138289
C 0.728748 -0.633196 2.391343
C 0.867905 0.442885 -2.318464
C -0.833365 1.125935 2.195032
C -2.004075 -1.763431 -0.319538
C 1.895298 1.580334 -0.706319
C -1.759148 1.946782 0.338374
C 1.638441 -1.903064 0.807715
H -0.067312 0.460850 4.097474
H -0.001604 -0.562471 -4.027907
N 1.557499 -1.534400 2.956224
N 1.836802 1.196418 -2.887076
N -1.687015 2.127835 2.543822
N -1.874346 -1.964957 -2.520932
C 2.498956 1.918893 -1.876035
C 2.168365 -2.342486 1.995042
C -2.274130 2.650352 1.377603
C -2.565676 -2.407123 -1.377281
N 2.142063 2.009555 0.597255
N -2.006065 2.048367 -1.035837
N -2.321060 -1.810975 1.037501
N 1.832353 -2.322580 -0.519005
N 3.567879 2.777203 -2.084293
N 3.755220 -2.823694 2.565481
N -3.195115 3.691546 1.342382
N -3.594715 -3.337178 -1.383978
C 2.129011 1.195832 -4.291414
H 2.337266 0.180747 -4.639568
H 1.292615 1.598529 -4.870218
H 3.006304 1.809941 -4.473028
C -1.909876 2.527768 3.900629
H -2.336493 1.707593 4.486983
H -0.971168 2.832896 4.373035
H -2.598695 3.367373 3.923127
C 1.788363 -1.644574 4.372190
H 1.994877 -0.662932 4.804725
H 0.924795 -2.080890 4.883020
H 2.662241 -2.283850 4.490704
C -2.088586 -2.369916 -3.879916
H -2.426002 -1.530527 -4.495119
H -1.166764 -2.769054 -4.311152
H -2.846391 -3.147651 -3.905427
C -1.434570 3.209843 -1.683129
H -1.491317 3.079706 -2.767174
H -1.950272 4.146828 -1.421889
H -0.382678 3.301657 -1.411748
C -3.369749 1.787034 -1.442254
H -4.068365 2.589080 -1.161489
H -3.401437 1.671227 -2.529143
H -3.711320 0.852968 -0.995197
C -3.715219 -1.615277 1.367292
H -4.346427 -2.484684 1.133529
H -3.796145 -1.412892 2.438463
H -4.104774 -0.748974 0.831127
C -1.712529 -2.894064 1.788932
H -1.800095 -2.678882 2.856143
H -2.196164 -3.863489 1.590596
H -0.652305 -2.974080 1.550860
C 3.286909 1.398258 1.251400
H 4.244517 1.784186 0.870760
H 3.235809 1.607359 2.321907
H 3.275884 0.315445 1.130237
C 2.071394 3.438866 0.806634
H 2.046672 3.634195 1.881689
H 2.925040 3.985455 0.379303
H 1.151204 3.833867 0.373421
C 3.141563 -2.048739 -1.076432
H 3.932709 -2.694074 -0.669845
H 3.107022 -2.189630 -2.161777
H 3.421347 -1.014855 -0.873207
C 1.393688 -3.672706 -0.802396
H 1.431739 -3.846238 -1.882851
H 2.009895 -4.439606 -0.310097
H 0.361880 -3.802763 -0.472794
C -4.868673 -2.949422 -1.946743
H -4.925535 -3.045929 -3.042488
H -5.659008 -3.576104 -1.523920
H -5.082588 -1.912960 -1.685872
C 4.749838 -1.967506 2.256470
H 4.812131 -1.049154 2.836897
H 5.167071 -1.927884 1.242909
C 3.935868 -4.170321 2.089995
H 4.205061 -4.238695 1.021899
H 4.756789 -4.625589 2.651882
H 3.010737 -4.737215 2.241399
C 4.868964 2.195090 -2.336902
H 5.032917 1.908059 -3.387638
H 5.646549 2.915295 -2.068240
H 4.998885 1.308881 -1.716835
C 3.318088 4.034279 -2.752508
H 4.103341 4.747962 -2.488131
H 3.295419 3.964596 -3.851681
H 2.363990 4.442165 -2.418258
C -4.529256 3.427685 1.837525
H -4.621962 3.485628 2.933193
H -5.224922 4.156359 1.411858
H -4.843728 2.432721 1.522878
C -2.709152 5.028396 1.610085
H -3.402940 5.758715 1.184139
H -2.602204 5.261400 2.681107
H -1.737633 5.163920 1.135389
C -3.250065 -4.732017 -1.556016
H -4.041508 -5.357015 -1.132860
H -3.118301 -5.033069 -2.607423
H -2.324749 -4.946845 -1.022720

14b, neutral form, tautomer 3, transition state
Stoichiometry: $C_{34}H_{61}BN_{16}$
Charge: 0
Multiplicity: 1
Point group: C_1
DLPNO-CCSD(T), Hartree: -2229.2002209
Gibbs in HMPA, Hartree: -2228.3527675
Nuclear coordinates, Å:
B 0.078520 -0.027050 0.015876
N -0.784825 -0.861986 -0.962785
N 1.056424 -0.941508 0.787627
N -0.783178 0.689401 1.054729
N 0.843619 0.985461 -0.876060
C -0.137705 0.303216 -2.987863
C 0.212089 -0.411143 2.995686
C 0.718899 1.079636 -2.220150
C -0.697418 0.473104 2.390561
C -0.916368 -0.612108 -2.280930
C 1.067126 -1.076663 2.138425
C 1.761764 1.943311 -0.455992

C	-1.749695	-1.795188	-0.600106
C	2.057647	-1.750231	0.259708
C	-1.758777	1.662633	0.779471
H	0.253267	-0.548247	4.063441
H	-0.289411	0.491535	-4.036515
N	-1.589270	1.265730	2.996977
N	-1.908988	-1.387727	-2.766448
N	2.053524	-1.954328	2.464427
N	1.547237	2.058273	-2.656046
C	-2.460620	-2.125945	-1.707331
C	-2.256715	2.033021	1.994528
C	2.683909	-2.386097	1.282407
C	2.202809	2.622632	-1.545955
N	-1.861004	-2.242897	0.718471
N	2.288888	-1.809799	-1.118014
N	2.092280	2.085633	0.892252
N	-2.697853	1.497681	-0.756965
N	-3.537991	-2.992557	-1.820458
N	-3.280959	2.919539	2.288328
N	3.731869	-3.297046	1.223771
N	3.098261	3.681706	-1.605860
C	-2.419951	-1.285395	-4.103894
H	-2.934498	-0.331902	-4.251494
H	-1.606893	-1.371346	-4.828202
H	-3.122698	-2.095014	-4.280019
C	2.360298	-2.326363	3.812528
H	2.682401	-1.458284	4.396707
H	1.484707	-2.762973	4.302646
H	3.159478	-3.062150	3.811939
C	-1.762160	1.353881	4.417236
H	-2.187659	0.433769	4.829853
H	-0.808524	1.549659	4.916450
H	-2.441248	2.174813	4.634228
C	1.637458	2.467207	-4.027245
H	1.945760	1.631359	-4.661698
H	0.674257	2.840024	-4.387477
H	2.372373	3.262013	-4.116643
C	1.770203	-2.985549	-1.784636
H	1.787578	-2.821295	-2.864909
H	2.350877	-3.895024	-1.563412
H	0.733991	-3.149838	-1.490330
C	3.625065	-1.458556	-1.544874
H	4.376028	-2.225274	-1.303638
H	3.623452	-1.310732	-2.628237
H	3.922921	-0.518718	-1.078790
C	3.502942	2.055543	1.204496
H	4.037373	2.967831	0.901541
H	3.621270	1.933797	2.284614
H	3.971339	1.199381	0.716972
C	1.378318	3.139926	1.588756
H	1.468580	2.982184	2.666580
H	1.774471	4.140084	1.351535
H	0.320812	3.106757	1.325607
C	-3.076036	-1.855254	1.406926
H	-3.954394	-2.430352	1.078031
H	-2.940154	-2.015558	2.480026
H	-3.269140	-0.796677	1.238078
C	-1.495271	-3.623178	0.939185
H	-1.395658	-3.797588	2.014181
H	-2.234907	-4.337668	0.544885
H	-0.529453	-3.831186	0.476116
C	-4.024715	1.317433	-0.642981
H	-4.688025	2.165896	-0.434191
H	-4.364508	0.355348	-0.265372
C	-2.360411	2.717958	-1.455749
H	-2.639231	2.603997	-2.508836
H	-2.894501	3.599302	-1.066306
H	-1.287162	2.910772	-1.366554
C	4.402056	3.444670	-2.183502
H	4.424926	3.498920	-3.283586
H	5.107927	4.190674	-1.807051
H	4.759104	2.458920	-1.885408
C	-4.549117	2.390374	2.724842
H	-4.598332	2.173597	3.806562
H	-5.345468	3.106958	2.501755
H	-4.763635	1.476682	2.170545
C	-2.963213	4.247910	2.741194
H	-3.774542	4.934922	2.476425
H	-2.809448	4.332663	3.832453
H	-2.054814	4.589872	2.245431
C	-4.843005	-2.411014	-2.072068
H	-5.092827	-2.358490	-3.142843
H	-5.616190	-3.012817	-1.584394
H	-4.876828	-1.398055	-1.669820
C	-3.318954	-4.277980	-2.441101
H	-4.101027	-4.973430	-2.122750
H	-3.335135	-4.250342	-3.543316
H	-2.356809	-4.681449	-2.124951
C	5.031437	-2.865841	1.691280
H	5.158329	-2.921877	2.783849
H	5.806172	-3.492618	1.240474
H	5.205334	-1.835288	1.382242
C	3.430976	-4.689045	1.479092
H	4.206195	-5.316632	1.030224
H	3.376076	-4.947676	2.548447
H	2.476839	-4.944900	1.019059
C	2.562990	5.006711	-1.834538
H	3.264213	5.753502	-1.451036
H	2.382234	5.237746	-2.896208
H	1.620869	5.114937	-1.298381

14bH$^+$·**14b** complex
Stoichiometry: $C_{68}H_{123}B_2N_{32}^+$
Charge: +1
Multiplicity: 1
Point group: C_2
DLPNO-CCSD(T), Hartree: -4459.1134924
Gibbs in HMPA, Hartree: -4457.3774210
Nuclear coordinates, Å:

B	0.000000	0.000000	-4.803788
N	1.185393	0.357836	-5.763856
N	-0.352225	1.159290	-3.889210
N	0.352225	-1.159290	-3.889210
N	-1.185393	-0.357836	-5.763856
C	0.000000	0.000000	-7.851519
C	0.000000	0.000000	-1.709980
C	-1.134534	-0.323717	-7.112821
C	0.347274	-1.077853	-2.510736
C	1.134534	0.323717	-7.112821
C	-0.347274	1.077853	-2.510736
C	-2.481487	-0.703099	-5.387690
C	2.481487	0.703099	-5.387690
C	-0.789329	2.419102	-4.281964
C	0.789329	-2.419102	-4.281964
H	0.000000	0.000000	-8.927873
N	0.763469	-2.318946	-2.073807
N	2.357169	0.643131	-7.596855
N	-0.763469	2.318946	-2.073807
N	-2.357169	-0.643131	-7.596855
C	3.219926	0.891408	-6.513902
C	1.045878	-3.144346	-3.164996
C	-1.045878	3.144346	-3.164996
C	-3.219926	-0.891408	-6.513902
N	2.850571	0.786714	-4.046127
N	-0.907449	2.763866	-5.636595
N	-2.850571	-0.786714	-4.046127
N	0.907449	-2.763866	-5.636595
N	4.552679	1.260291	-6.626008
N	1.482077	-4.467159	-3.087195
N	-1.482077	4.467159	-3.087195
N	-4.552679	-1.260291	-6.626008
C	2.671009	0.704921	-8.996191
H	2.565556	-0.276608	-9.466708
H	2.011003	1.409548	-9.508692
H	3.696187	1.041560	-9.118269
C	-0.973810	2.634890	-0.693599
H	-0.688352	3.667426	-0.489389
H	-2.019726	2.497544	-0.394127
H	-0.362260	1.958506	-0.099313
C	0.973810	-2.634890	-0.693599
H	0.688352	-3.667426	-0.489389
H	2.019726	-2.497544	-0.394127
H	0.362260	-1.958506	-0.099313
C	-2.671009	-0.704921	-8.996191
H	-2.565556	0.276608	-9.466708
H	-2.011003	-1.409548	-9.508692
H	-3.696187	-1.041560	-9.118269
C	0.127116	3.639089	-6.147229
H	0.068694	3.665666	-7.238705
H	0.040273	4.671415	-5.774778
H	1.109263	3.252173	-5.874387
C	-2.232744	3.165745	-6.058731
H	-2.532557	4.149263	-5.669667
H	-2.261156	3.206423	-7.151203
H	-2.961540	2.423685	-5.731041
C	-4.004144	-0.009976	-3.652965
H	-4.958354	-0.443978	-3.984887
H	-4.020591	0.064542	-2.562195
H	-3.921591	1.000578	-4.053493
C	-2.833207	-2.112214	-3.465562
H	-2.861077	-2.024042	-2.377164
H	-3.686516	-2.729750	-3.788945
H	-1.908279	-2.623688	-3.728490
C	4.004144	0.009976	-3.652965

H	4.958354	0.443978	-3.984887
H	4.020591	-0.064542	-2.562195
H	3.921591	-1.000578	-4.053493
C	2.833207	2.112214	-3.465562
H	2.861077	2.024042	-2.377164
H	3.686516	2.729750	-3.788945
H	1.908279	2.623688	-3.728490
C	2.232744	-3.165745	-6.058731
H	2.532557	-4.149263	-5.669667
H	2.261156	-3.206423	-7.151203
H	2.961540	-2.423685	-5.731041
C	-0.127116	-3.639089	-6.147229
H	-0.068694	-3.665666	-7.238705
H	-0.040273	-4.671415	-5.774778
H	-1.109263	-3.252173	-5.874387
C	-5.499391	-0.268304	-7.089751
H	-5.541131	-0.163441	-8.184676
H	-6.502884	-0.538259	-6.750014
H	-5.247734	0.703264	-6.664867
C	2.803198	-4.700170	-2.544812
H	2.845274	-4.672782	-1.444980
H	3.156990	-5.684799	-2.862871
H	3.493599	-3.950514	-2.932060
C	0.507298	-5.467679	-2.709313
H	0.853674	-6.452820	-3.034334
H	0.322726	-5.525748	-1.624527
H	-0.440654	-5.259449	-3.204813
C	5.499391	0.268304	-7.089751
H	5.541131	0.163441	-8.184676
H	6.502884	0.538259	-6.750014
H	5.247734	-0.703264	-6.664867
C	4.849348	2.618579	-7.028707
H	5.857267	2.882760	-6.697539
H	4.806603	2.782911	-8.116682
H	4.145422	3.301515	-6.553801
C	-2.803198	4.700170	-2.544812
H	-2.845274	4.672782	-1.444980
H	-3.156990	5.684799	-2.862871
H	-3.493599	3.950514	-2.932060
C	-0.507298	5.467679	-2.709313
H	-0.853674	6.452820	-3.034334
H	-0.322726	5.525748	-1.624527
H	0.440654	5.259449	-3.204813
C	-4.849348	-2.618579	-7.028707
H	-5.857267	-2.882760	-6.697539
H	-4.806603	-2.782911	-8.116682
H	-4.145422	-3.301515	-6.553801
H	0.000000	0.000000	0.720371
C	0.000000	0.000000	1.809728
C	-1.137222	-0.296942	2.548058
C	1.137222	0.296942	2.548058
N	-1.194333	-0.290961	3.904892
N	-2.362210	-0.640669	2.078221
N	1.194333	0.290961	3.904892
N	2.362210	0.640669	2.078221
B	0.000000	0.000000	4.826332
C	-2.484706	-0.651337	4.291847
C	-3.217208	-0.865731	3.168880
C	-2.677848	-0.837300	0.683252
C	2.484706	0.651337	4.291847
C	3.217208	0.865731	3.168880
C	2.677848	0.837300	0.683252
N	-0.288656	1.197808	5.765306
N	0.288656	-1.197808	5.765306
N	-2.834689	-0.749116	5.642691
N	-4.560394	-1.213889	3.079603
H	-2.807508	-1.899468	0.460714
H	-3.599001	-0.312964	0.426649
H	-1.869128	-0.456426	0.050435
N	2.834689	0.749116	5.642691
N	4.560394	1.213889	3.079603
H	2.807508	1.899468	0.460714
H	3.599001	0.312964	0.426649
H	1.869128	0.456426	0.050435
C	-0.261728	1.149705	7.115815
C	-0.569722	2.507353	5.379188
C	0.261728	-1.149705	7.115815
C	0.569722	-2.507353	5.379188
C	-3.621196	0.353342	6.160723
C	-3.348041	-2.040168	6.056561
C	-5.490548	-0.176222	2.683033
C	-4.881612	-2.524870	2.553122
C	3.348041	2.040168	6.056561
C	3.621196	-0.353342	6.160723
C	5.490548	0.176222	2.683033
C	4.881612	2.524870	2.553122
C	0.000000	0.000000	7.855595
N	-0.523759	2.389141	7.589781
C	-0.723326	3.258466	6.501104
N	-0.642095	2.861779	4.030743
N	0.523759	-2.389141	7.589781
C	0.723326	-3.258466	6.501104
N	0.642095	-2.861779	4.030743
H	-4.655256	0.354927	5.785300
H	-3.654418	0.286703	7.250914
H	-3.152432	1.301674	5.897461
H	-3.386096	-2.071374	7.148406
H	-4.354519	-2.249858	5.669906
H	-2.673053	-2.828072	5.721135
H	-5.524517	-0.002612	1.596892
H	-6.498148	-0.454305	3.002778
H	-5.225244	0.760828	3.172058
H	-5.879466	-2.814588	2.892587
H	-4.878088	-2.574310	1.454644
H	-4.169150	-3.257109	2.933345
H	3.386096	2.071374	7.148406
H	4.354519	2.249858	5.669906
H	2.673053	2.828072	5.721135
H	4.655256	-0.354927	5.785300
H	3.654418	-0.286703	7.250914
H	3.152432	-1.301674	5.897461
H	5.524517	0.002612	1.596892
H	6.498148	0.454305	3.002778
H	5.225244	-0.760828	3.172058
H	5.879466	2.814588	2.892587
H	4.878088	2.574310	1.454644
H	4.169150	3.257109	2.933345
H	0.000000	0.000000	8.931908
C	-0.576235	2.714250	8.988296
N	-1.019757	4.609226	6.601985
C	-1.980803	3.048662	3.507180
C	0.279699	3.894979	3.604724
C	0.576235	-2.714250	8.988296
N	1.019757	-4.609226	6.601985
C	1.980803	-3.048662	3.507180
C	-0.279699	-3.894979	3.604724
H	-1.329551	2.108180	9.497701
H	0.392030	2.543066	9.465928
H	-0.840268	3.761210	9.101945
C	-2.355932	4.981317	7.019460
C	0.028705	5.503647	7.048195
H	-2.458385	3.970249	3.872292
H	-1.932895	3.095095	2.416951
H	-2.607681	2.198367	3.775652
H	0.297208	3.921743	2.512441
H	0.009825	4.895422	3.970650
H	1.285895	3.654269	3.948916
H	1.329551	-2.108180	9.497701
H	-0.392030	-2.543066	9.465928
H	0.840268	-3.761210	9.101945
C	2.355932	-4.981317	7.019460
C	-0.028705	-5.503647	7.048195
H	2.458385	-3.970249	3.872292
H	1.932895	-3.095095	2.416951
H	2.607681	-2.198367	3.775652
H	-0.297208	-3.921743	2.512441
H	-0.009825	-4.895422	3.970650
H	-1.285895	-3.654269	3.948916
H	-2.508763	4.954054	8.109052
H	-2.567925	5.999718	6.684414
H	-3.082596	4.313826	6.556801
H	-0.193792	6.518213	6.708359
H	0.151182	5.542196	8.141017
H	0.979146	5.200314	6.609440
H	2.508763	-4.954054	8.109052
H	2.567925	-5.999718	6.684414
H	3.082596	-4.313826	6.556801
H	0.193792	-6.518213	6.708359
H	-0.151182	-5.542196	8.141017
H	-0.979146	-5.200314	6.609440

14bH$^+$·**14b** complex, transition state

Stoichiometry: $C_{68}H_{123}B_2N_{32}^+$

Charge: +1

Multiplicity: 1

Point group: C_1

DLPNO-CCSD(T), Hartree: -4459.0638008

Gibbs in HMPA, Hartree: -4457.3290523

Nuclear coordinates, Å:

B	-5.018247	0.019311	-0.133793
N	-5.895053	-1.205322	-0.516110
N	-3.808928	0.148997	-1.063181
N	-4.457954	-0.116099	1.284215
N	-5.958150	1.250599	-0.257961

C	-7.932232	0.032543	-0.970887
C	-2.114363	0.013623	0.664779
C	-7.253442	1.201477	-0.639547
C	-3.132319	-0.113272	1.589433
C	-7.194215	-1.144095	-0.882446
C	-2.499461	0.146130	-0.686766
C	-5.631327	2.579572	0.007226
C	-5.501781	-2.542559	-0.524995
C	-3.837080	0.293491	-2.446718
C	-5.183066	-0.262358	2.464673
H	-1.077931	0.019364	0.955413
H	-8.969839	0.035960	-1.256831
N	-3.020395	-0.256620	2.937994
N	-7.628782	-2.402168	-1.123488
N	-1.710913	0.280620	-1.759833
N	-7.750318	2.459507	-0.623677
C	-6.565898	-3.297942	-0.905303
C	-4.308682	-0.349068	3.497021
C	-2.540073	0.371273	-2.864834
C	-6.732500	3.343352	-0.220213
N	-4.207030	-2.919034	-0.165167
N	-5.048624	0.340489	-3.151045
N	-4.356039	2.937963	0.446360
N	-6.582294	-0.300862	2.451857
N	-6.636922	-4.673325	-1.068843
N	-4.585047	-0.499205	4.850021
N	-2.119580	0.526722	-4.192881
N	-6.871548	4.718200	-0.102782
C	-8.967816	-2.719203	-1.534592
H	-9.690821	-2.455991	-0.757693
H	-9.226525	-2.180293	-2.449396
H	-9.038882	-3.784958	-1.729909
C	0.175442	0.238826	-1.679408
H	0.262052	1.113896	-1.064027
H	0.217783	-0.725854	-1.211021
H	0.302856	0.321303	-2.737737
C	-1.764833	-0.290428	3.626465
H	-1.140564	-1.110063	3.258797
H	-1.216076	0.645892	3.488571
H	-1.940165	-0.436986	4.688273
C	-9.104260	2.787463	-0.972731
H	-9.338603	2.430098	-1.978494
H	-9.811715	2.340077	-0.269201
H	-9.227469	3.866071	-0.952090
C	-5.294960	-0.785888	-4.028643
H	-6.333674	-0.754384	-4.368695
H	-4.643849	-0.791770	-4.914835
H	-5.149009	-1.718381	-3.482059
C	-5.342137	1.610041	-3.785284
H	-4.690606	1.822209	-4.645227
H	-6.378241	1.604224	-4.134527
H	-5.237153	2.417735	-3.059890
C	-3.471390	3.470726	-0.571169
H	-3.741035	4.494143	-0.874556
H	-2.450739	3.484484	-0.182302
H	-3.482198	2.827159	-1.450214
C	-4.304524	3.687315	1.684025
H	-3.273266	3.696304	2.045732
H	-4.641560	4.727844	1.577509
H	-4.919230	3.196246	2.438929
C	-4.107970	-3.904045	0.891013
H	-4.393032	-4.916092	0.571356
H	-3.074736	-3.934610	1.245924
H	-4.740472	-3.611044	1.729422
C	-3.303630	-3.182527	-1.267717
H	-2.280808	-3.223694	-0.886146
H	-3.522119	-4.133430	-1.777867
H	-3.354026	-2.372121	-1.994227
C	-7.169095	-1.560744	2.860187
H	-7.064610	-1.758314	3.936864
H	-8.234708	-1.552794	2.616975
H	-6.705182	-2.377832	2.306861
C	-7.235721	0.844340	3.052080
H	-8.300861	0.815347	2.808623
H	-7.134812	0.874670	4.146899
H	-6.821831	1.763268	2.635902
C	-6.921130	5.500863	-1.320215
H	-7.915469	5.533325	-1.791432
H	-6.629487	6.531028	-1.099924
H	-6.214877	5.094889	-2.043969
C	-4.303937	-1.782020	5.459780
H	-3.249883	-1.922582	5.745029
H	-4.905014	-1.891498	6.366336
H	-4.578620	-2.580987	4.771369
C	-4.364022	0.640724	5.714977
H	-4.962783	0.527916	6.622629
H	-3.316007	0.771768	6.025758
H	-4.683935	1.550701	5.207708
C	-7.409773	-5.435012	-0.110086
H	-8.490802	-5.451294	-0.315535
H	-7.062641	-6.471484	-0.107526
H	-7.259322	-5.025142	0.888541
C	-6.658177	-5.195494	-2.419415
H	-6.316968	-6.233930	-2.410940
H	-7.653197	-5.179296	-2.890025
H	-5.976906	-4.618842	-3.044531
C	-1.491426	1.787876	-4.521720
H	-0.454764	1.876960	-4.162264
H	-1.476796	1.912077	-5.607753
H	-2.070129	2.607228	-4.094069
C	-1.479541	-0.617311	-4.805348
H	-1.479767	-0.493346	-5.891582
H	-0.435780	-0.768416	-4.489059
H	-2.040353	-1.521447	-4.566881
C	-7.678029	5.232209	0.984413
H	-7.384408	6.263632	1.195794
H	-8.759431	5.233871	0.780279
H	-7.501948	4.639677	1.882115
C	2.434162	0.169875	-1.561694
C	3.127514	1.258818	-1.046769
C	3.070034	-1.016277	-1.230596
N	4.237369	1.242160	-0.239883
N	2.841889	2.584523	-1.268585
N	4.191212	-1.179831	-0.450282
N	2.711546	-2.280800	-1.641463
B	4.968517	-0.027451	0.175147
C	4.625140	2.547289	0.038517
C	3.771925	3.387443	-0.598328
C	1.888202	3.031080	-2.245306
C	4.511834	-2.532744	-0.378415
C	3.607904	-3.220862	-1.120077
C	1.728021	-2.527343	-2.658629
N	6.452374	-0.013179	-0.312750
N	5.064910	-0.167563	1.725029
N	5.713176	2.820175	0.880312
N	3.767494	4.780710	-0.578949
H	0.916663	3.273574	-1.805476
H	2.263781	3.915613	-2.757942
H	1.753746	2.225607	-2.967834
N	5.591843	-2.986416	0.391967
N	3.535364	-4.597548	-1.320029
H	2.049014	-3.348248	-3.298478
H	0.746479	-2.776036	-2.245079
H	1.635047	-1.619544	-3.255638
C	7.537799	-0.110300	0.485289
C	6.890295	0.131098	-1.628645
C	6.212466	-0.258602	2.430852
C	4.000314	-0.203105	2.619651
C	6.844295	3.466822	0.248677
C	5.364381	3.423350	2.150358
C	4.319262	5.479647	-1.721518
C	2.605060	5.422259	-0.003684
C	6.732835	-3.470749	-0.357386
C	5.239052	-3.859274	1.493195
C	2.344158	-5.266915	-0.843949
C	4.057752	-5.134544	-2.559878
C	7.486342	-0.244885	1.870163
N	8.645220	-0.038812	-0.288881
C	8.249719	0.111847	-1.630952
N	5.986494	0.281784	-2.679336
N	5.899535	-0.349881	3.744792
C	4.499866	-0.319591	3.878202
N	2.680619	-0.114314	2.175942
H	6.657099	4.521245	-0.002337
H	7.702367	3.426462	0.925190
H	7.111164	2.931884	-0.663322
H	6.244564	3.425252	2.798764
H	5.006635	4.458701	2.052505
H	4.590689	2.829894	2.638471
H	3.614904	5.591537	-2.560391
H	4.625875	6.484600	-1.418241
H	5.199738	4.950587	-2.084197
H	2.856034	6.451846	0.264720
H	1.737419	5.458595	-0.680339
H	2.309504	4.896609	0.904740
H	7.587536	-3.570333	0.317007
H	6.555320	-4.448332	-0.830313
H	6.999457	-2.751938	-1.132714
H	4.893821	-4.850186	1.165389
H	6.115014	-3.995849	2.132797
H	4.454899	-3.397348	2.094039
H	1.475338	-5.156807	-1.510885
H	2.547068	-6.336231	-0.742214
H	2.073881	-4.878108	0.138295

H 4.313814 -6.188255 -2.418723
H 3.352422 -5.078668 -3.403566
H 4.964622 -4.598855 -2.837864
H 8.376647 -0.305014 2.472049
C 9.984775 -0.104789 0.224332
N 9.114834 0.211669 -2.710222
C 6.138540 1.477203 -3.480425
C 5.735216 -0.903830 -3.472029
C 6.877440 -0.453584 4.791122
N 3.808892 -0.402950 5.079234
C 1.954746 -1.363831 2.117597
C 1.904122 0.979066 2.713916
H 10.199646 0.747461 0.874863
H 10.133174 -1.025224 0.794535
H 10.685723 -0.095499 -0.604948
C 9.886596 1.428096 -2.855162
C 9.784371 -0.991483 -3.157838
H 7.010157 1.445265 -4.149675
H 5.241137 1.605890 -4.090421
H 6.226670 2.347633 -2.829691
H 4.836829 -0.744598 -4.072452
H 6.568404 -1.149169 -4.148978
H 5.551043 -1.756247 -2.818427
H 7.517121 -1.326115 4.635789
H 7.508062 0.438900 4.828435
H 6.371214 -0.561759 5.745605
C 3.758412 -1.690090 5.740839
C 3.862349 0.735612 5.972662
H 1.673669 -1.743592 3.113252
H 1.042701 -1.222777 1.532605
H 2.555506 -2.118825 1.611367
H 1.003534 1.106344 2.107272
H 1.599976 0.825020 3.760296
H 2.476835 1.904249 2.650633
H 10.799019 1.457637 -2.240331
H 10.192181 1.539977 -3.898605
H 9.269248 2.286219 -2.589798
H 10.092930 -0.867343 -4.199056
H 10.683284 -1.247250 -2.575874
H 9.094635 -1.833590 -3.106529
H 4.654596 -1.924430 6.335570
H 2.901200 -1.715044 6.418961
H 3.628304 -2.476931 4.998174
H 3.004828 0.704479 6.650033
H 4.769688 0.777341 6.594087
H 3.804165 1.657101 5.393581

14bH^+F^- ion pair
Stoichiometry: $C_{34}H_{62}BN_{16}F$
Charge: 0
Multiplicity: 1
Point group: C_2
DLPNO-CCSD(T), Hartree: -2329.6699281
Gibbs in HMPA, Hartree: -2328.8148285
Gibbs in gas, Hartree: -2328.7780681
Nuclear coordinates, Å:
B 0.000000 0.000000 -0.168538
N -0.568444 1.125979 0.720368
N -1.096614 -0.645497 -1.044558
N 0.568444 -1.125979 0.720368
N 1.096614 0.645497 -1.044558
C 1.015010 2.825582 0.022992
C -1.015010 -2.825582 0.022992
C 1.504523 1.933244 -0.917104
C 0.000000 -2.342599 0.856061
C 0.000000 2.342599 0.856061
C -1.504523 -1.933244 -0.917104
C 1.814429 0.061470 -2.088385
C -1.570072 0.997514 1.692955
C -1.814429 -0.061470 -2.088385
C 1.570072 -0.997514 1.692955
H -1.408807 -3.822311 0.123800
H 1.408807 3.822311 0.123800
N 0.588847 -2.973375 1.887374
N -0.588847 2.973375 1.887374
N -2.450852 -2.171044 -1.857211
N 2.450852 2.171044 -1.857211
C -1.597290 2.142867 2.419814
C 1.597290 -2.142867 2.419814
C -2.664089 -0.992727 -2.595862
C 2.664089 0.992727 -2.595862
N -2.317594 -0.170622 1.809707
N -1.598370 1.263218 -2.473636
N 1.598370 -1.263218 -2.473636
N 2.317594 0.170622 1.809707
N -2.389478 2.471680 3.501713
N 2.389478 -2.471680 3.501713
N -3.590052 -0.859055 -3.621083
N 3.590052 0.859055 -3.621083
C -0.193338 4.260576 2.383656
H 0.885769 4.284363 2.550547
H -0.463297 5.060283 1.687556
H -0.692153 4.435058 3.332574
C -3.121870 -3.429036 -2.003234
H -2.414702 -4.223073 -2.260957
H -3.630090 -3.706878 -1.075357
H -3.863217 -3.351028 -2.793150
C 0.193338 -4.260576 2.383656
H -0.885769 -4.284363 2.550547
H 0.463297 -5.060283 1.687556
H 0.692153 -4.435058 3.332574
C 3.121870 3.429036 -2.003234
H 2.414702 4.223073 -2.260957
H 3.630090 3.706878 -1.075357
H 3.863217 3.351028 -2.793150
C -2.433488 2.254102 -1.820664
H -2.015092 3.247336 -1.998768
H -3.468687 2.242423 -2.196886
H -2.450077 2.086927 -0.745740
C -1.483094 1.501426 -3.895667
H -2.447021 1.471857 -4.422294
H -1.037761 2.487906 -4.051754
H -0.825047 0.756990 -4.342772
C 1.483094 -1.501426 -3.895667
H 2.447021 -1.471857 -4.422294
H 1.037761 -2.487906 -4.051754
H 0.825047 -0.756990 -4.342772
C 2.433488 -2.254102 -1.820664
H 2.015092 -3.247336 -1.998768
H 3.468687 -2.242423 -2.196886
H 2.450077 -2.086927 -0.745740
C -2.432430 -0.736024 3.147749
H -3.257007 -0.278049 3.713051
H -2.646985 -1.806136 3.047684
H -1.494678 -0.586188 3.706107
C -3.578893 -0.166519 1.102975
H -3.969591 -1.187470 1.069303
H -4.334434 0.467533 1.594904
H -3.452389 0.179276 0.076432
C 2.432430 0.736024 3.147749
H 3.257007 0.278049 3.713051
H 2.646985 1.806136 3.047684
H 1.494678 0.586188 3.706107
C 3.578893 0.166519 1.102975
H 3.969591 1.187470 1.069303
H 4.334434 -0.467533 1.594904
H 3.452389 -0.179276 0.076432
C 3.334558 1.542602 -4.869745
H 3.655995 2.595811 -4.880255
H 3.864974 1.033064 -5.679384
H 2.267734 1.512104 -5.090516
C 1.821820 -2.344101 4.835582
H 1.321367 -3.268051 5.170957
H 2.631629 -2.140386 5.543358
H 1.106145 -1.505631 4.858344
C 3.381329 -3.502870 3.332045
H 4.183084 -3.356866 4.062166
H 2.997470 -4.527636 3.475187
H 3.817513 -3.440897 2.333482
C -1.821820 2.344101 4.835582
H -1.321367 3.268051 5.170957
H -2.631629 2.140386 5.543358
H -1.106145 1.505631 4.858344
C -3.381329 3.502870 3.332045
H -4.183084 3.356866 4.062166
H -2.997470 4.527636 3.475187
H -3.817513 3.440897 2.333482
C -3.334558 -1.542602 -4.869745
H -3.655995 -2.595811 -4.880255
H -3.864974 -1.033064 -5.679384
H -2.267734 -1.512104 -5.090516
C -4.988007 -0.749856 -3.263970
H -5.531727 -0.251094 -4.071454
H -5.479977 -1.718760 -3.084180
H -5.087714 -0.146567 -2.362264
C 4.988007 0.749856 -3.263970
H 5.531727 0.251094 -4.071454
H 5.479977 1.718760 -3.084180
H 5.087714 0.146567 -2.362264
F 0.000000 0.000000 4.586163

15a, neutral form
Stoichiometry: $C_{12}H_{30}P_4Pt$
Charge: 0

Multiplicity: 1
Point group: C_3
DLPNO-CCSD(T), Hartree: -1957.4134916
Gibbs in HMPA, Hartree: -1957.0815938
Nuclear coordinates, Å:

C	1.197293	-2.460558	-1.312620
H	0.281967	-3.059085	-1.359466
H	2.029279	-3.136679	-1.536561
C	1.532259	2.267165	-1.312620
H	1.701804	3.325746	-1.536561
H	2.508262	1.773733	-1.359466
C	-2.729552	0.193393	-1.312620
H	-2.790229	1.285352	-1.359466
H	-3.731083	-0.189068	-1.536561
C	1.121854	-1.336421	-2.352040
H	2.102465	-0.867505	-2.478459
H	0.823019	-1.726378	-3.329759
C	0.596447	1.639764	-2.352040
H	-0.299951	2.254541	-2.478459
H	1.083578	1.575945	-3.329759
C	-1.718301	-0.303344	-2.352040
H	-1.802514	-1.387036	-2.478459
H	-1.906597	0.150434	-3.329759
P	1.290928	-1.878593	0.479949
P	0.981446	2.057273	0.479949
P	-2.272374	-0.178680	0.479949
Pt	0.000000	0.000000	0.503601
P	0.000000	0.000000	-1.732720
C	0.000000	3.598680	0.729055
H	-0.294514	3.659962	1.777427
H	0.561164	4.497468	0.458621
H	-0.909398	3.545635	0.129075
C	3.116548	-1.799340	0.729055
H	3.316877	-1.574924	1.777427
H	3.614339	-2.734716	0.458621
H	3.525309	-0.985256	0.129075
C	-3.116548	-1.799340	0.729055
H	-3.022363	-2.085037	1.777427
H	-4.175503	-1.762751	0.458621
H	-2.615911	-2.560379	0.129075
C	0.948383	-3.488690	1.308411
H	1.572133	-4.299568	0.920802
H	1.132688	-3.374552	2.377241
H	-0.103761	-3.742472	1.174992
C	2.547102	2.565669	1.308411
H	2.937468	3.511291	0.920802
H	2.356104	2.668213	2.377241
H	3.292956	1.781376	1.174992
C	-3.495485	0.923021	1.308411
H	-4.509601	0.788276	0.920802
H	-3.488792	0.706339	2.377241
H	-3.189195	1.961096	1.174992

15a, protonated form
Stoichiometry: $C_{12}H_{31}P_4Pt^+$
Charge: +1
Multiplicity: 1
Point group: C_3
DLPNO-CCSD(T), Hartree: -1957.8894944
Gibbs in HMPA, Hartree: -1957.5877633
Nuclear coordinates, Å:

C	0.574761	-2.678049	-1.380737
H	-0.458002	-3.028282	-1.463419
H	1.218463	-3.528871	-1.618122
C	2.031877	1.836782	-1.380737
H	2.446860	2.819655	-1.618122
H	2.851570	1.117500	-1.463419
C	-2.606639	0.841266	-1.380737
H	-2.393568	1.910782	-1.463419
H	-3.665323	0.709216	-1.618122
C	0.819494	-1.525996	-2.352839
H	1.886785	-1.296738	-2.418656
H	0.481900	-1.771114	-3.362770
C	0.911804	1.472701	-2.352839
H	0.179616	2.282372	-2.418656
H	1.292879	1.302895	-3.362770
C	-1.731298	0.053295	-2.352839
H	-2.066400	-0.985635	-2.418656
H	-1.774780	0.468219	-3.362770
P	0.797629	-2.176534	0.397396
P	1.486119	1.779034	0.397396
P	-2.283748	0.397500	0.397396
Pt	0.000000	0.000000	0.543999
P	0.000000	0.000000	-1.724212
C	0.880347	3.474454	0.719994
H	0.559291	3.541865	1.759989
H	1.657955	4.218814	0.536542
H	0.019390	3.688221	0.086061
C	2.568792	-2.499629	0.719994
H	2.787700	-2.255293	1.759989
H	2.824623	-3.545239	0.536542
H	3.184398	-1.860902	0.086061
C	-3.449138	-0.974824	0.719994
H	-3.346991	-1.286572	1.759989
H	-4.482578	-0.673576	0.536542
H	-3.203788	-1.827318	0.086061
C	0.000000	-3.541695	1.307662
H	0.415604	-4.509749	1.020764
H	0.157593	-3.389246	2.375831
H	-1.073101	-3.533583	1.118523
C	3.067198	1.770847	1.307662
H	3.697755	2.614798	1.020764
H	2.856377	1.831103	2.375831
H	3.596723	0.837459	1.118523
C	-3.067198	1.770847	1.307662
H	-4.113359	1.894951	1.020764
H	-3.013970	1.558143	2.375831
H	-2.523622	2.696124	1.118523
H	0.000000	0.000000	2.150734

15b, neutral form
Stoichiometry: $C_{24}H_{54}P_4Pt$
Charge: 0
Multiplicity: 1
Point group: C_3
DLPNO-CCSD(T), Hartree: -2428.2816901
Gibbs in HMPA, Hartree: -2427.6189894
Nuclear coordinates, Å:

C	-2.685195	0.594329	-1.729814
H	-2.614861	1.685496	-1.768207
H	-3.721207	0.341445	-1.975143
C	0.827894	-2.622612	-1.729814
H	1.564903	-3.393383	-1.975143
H	-0.152252	-3.107285	-1.768207
C	1.857301	2.028283	-1.729814
H	2.767113	1.421788	-1.768207
H	2.156304	3.051937	-1.975143
P	-2.301571	0.158889	0.072977
P	1.013184	-2.072664	0.072977
P	1.288388	1.913775	0.072977
Pt	0.000000	0.000000	0.097388
P	0.000000	0.000000	-2.139385
C	-3.213534	1.580976	0.901143
H	-2.639698	2.434182	0.524127
C	0.237602	-3.573491	0.901143
H	-0.788215	-3.503137	0.524127
C	2.975933	1.992514	0.901143
H	3.427913	1.068954	0.524127
C	0.622925	3.652593	0.337916
H	1.478488	4.330667	0.258768
C	2.851776	-2.365765	0.337916
H	3.011224	-3.445742	0.258768
C	-3.474701	-1.286828	0.337916
H	-4.489712	-0.884925	0.258768
C	3.684653	-1.688707	-0.742088
H	3.542658	-2.146472	-1.722113
H	3.431302	-0.629164	-0.824845
H	4.748604	-1.758658	-0.498389
C	-0.379863	4.035356	-0.742088
H	0.087571	4.141268	-1.722113
H	-1.170779	3.286177	-0.824845
H	-0.851259	4.991741	-0.498389
C	-3.304789	-2.346649	-0.742088
H	-3.630228	-1.994795	-1.722113
H	-2.260523	-2.657013	-0.824845
H	-3.897344	-3.233082	-0.498389
C	0.174306	-3.419963	2.416052
H	-0.091408	-2.399666	2.700219
H	-0.567280	-4.105280	2.836599
H	1.132593	-3.663042	2.879779
C	-3.048928	1.559028	2.416052
H	-2.032468	1.278995	2.700219
H	-3.271637	2.543919	2.836599
H	-3.738584	0.850667	2.879779
C	2.874622	1.860935	2.416052
H	2.123876	1.120671	2.700219
H	3.838917	1.561361	2.836599
H	2.605991	2.812375	2.879779
C	0.790283	-4.939604	0.515071
H	1.804378	-5.085748	0.894155
H	0.172305	-5.729228	0.953662
H	0.806616	-5.099823	-0.564068
C	-4.672964	1.785397	0.515071
H	-5.306576	0.980237	0.894155

H	-5.047809	2.715394	0.953662
H	-4.819884	1.851362	-0.564068
C	3.882681	3.154207	0.515071
H	3.502198	4.105511	0.894155
H	4.875505	3.013834	0.953662
H	4.013268	3.248461	-0.564068
C	3.287908	-1.898275	1.719052
H	2.797911	-2.450207	2.520106
H	4.368389	-2.023610	1.838925
H	3.044894	-0.844186	1.859986
C	0.000000	3.796549	1.719052
H	0.722986	3.648166	2.520106
H	-0.431697	4.794941	1.838925
H	-0.791360	3.059049	1.859986
C	-3.287908	-1.898275	1.719052
H	-3.520897	-1.197959	2.520106
H	-3.936692	-2.771331	1.838925
H	-2.253534	-2.214863	1.859986
C	0.863962	1.506217	-2.775893
H	1.368991	1.325654	-3.729736
H	0.080281	2.244605	-2.962284
C	0.872441	-1.501322	-2.775893
H	0.463555	-1.848409	-3.729736
H	1.903744	-1.191828	-2.962284
C	-1.736403	-0.004895	-2.775893
H	-1.832546	0.522754	-3.729736
H	-1.984025	-1.052777	-2.962284

15b, protonated form
Stoichiometry: $C_{24}H_{55}P_4Pt^+$
Charge: +1
Multiplicity: 1
Point group: C_3
DLPNO-CCSD(T), Hartree: -2428.7632523
Gibbs in HMPA, Hartree: -2428.1276804
Nuclear coordinates, Å:

C	0.387586	2.720858	-1.781509
H	1.478980	2.713354	-1.849732
H	0.066030	3.731965	-2.040903
C	-2.550125	-1.024770	-1.781509
H	-3.264992	-1.808799	-2.040903
H	-3.089324	-0.075843	-1.849732
C	2.162540	-1.696088	-1.781509
H	1.610344	-2.637511	-1.849732
H	3.198962	-1.923167	-2.040903
P	0.000000	2.355670	0.009319
P	-2.040070	-1.177835	0.009319
P	2.040070	-1.177835	0.009319
Pt	0.000000	0.000000	0.159730
P	0.000000	0.000000	-2.106666
C	1.362354	3.298339	0.866532
H	2.247936	2.762787	0.509613
C	-3.537623	-0.469337	0.866532
H	-3.516612	0.565376	0.509613
C	2.175269	-2.829003	0.866532
H	1.268676	-3.328164	0.509613
C	3.682870	-0.355166	0.335091
H	4.421631	-1.161801	0.300267
C	-2.149018	-3.011876	0.335091
H	-3.216965	-3.248344	0.300267
C	-1.533852	3.367042	0.335091
H	-1.204666	4.410145	0.300267
C	-1.453529	-3.826790	-0.746967
H	-1.942310	-3.738199	-1.717940
H	-0.408315	-3.528867	-0.859612
H	-1.464343	-4.884738	-0.475525
C	4.040862	0.654602	-0.746967
H	4.208531	0.187010	-1.717940
H	3.260246	1.410822	-0.859612
H	4.962479	1.174210	-0.475525
C	-2.587333	3.172188	-0.746967
H	-2.266221	3.551190	-1.717940
H	-2.851931	2.118045	-0.859612
H	-3.498136	3.710527	-0.475525
C	-3.396160	-0.438600	2.384276
H	-2.400285	-0.125204	2.701056
H	-4.124081	0.257310	2.807483
H	-3.602651	-1.416442	2.821093
C	1.318242	3.160461	2.384276
H	1.091713	2.141309	2.701056
H	2.284878	3.442904	2.807483
H	0.574651	3.828208	2.821093
C	2.077919	-2.721861	2.384276
H	1.308572	-2.016106	2.701056
H	1.839203	-3.700214	2.807483
H	3.028000	-2.411766	2.821093
C	-4.870089	-1.085691	0.455804
H	-4.966189	-2.115247	0.805424
H	-5.685932	-0.519949	0.912387
H	-5.032659	-1.074705	-0.622632
C	1.494809	4.760467	0.455804
H	0.651237	5.358469	0.805424
H	2.392677	5.184136	0.912387
H	1.585608	4.895763	-0.622632
C	3.375281	-3.674776	0.455804
H	4.314952	-3.243222	0.805424
H	3.293255	-4.664187	0.912387
H	3.447051	-3.821058	-0.622632
C	-1.607846	-3.368195	1.713105
H	-2.156427	-2.885186	2.519435
H	-1.673349	-4.447742	1.868055
H	-0.562818	-3.073422	1.808059
C	3.720866	0.291662	1.713105
H	3.576858	-0.424927	2.519435
H	4.688532	0.774708	1.868055
H	2.943071	1.049296	1.808059
C	-2.113019	3.076533	1.713105
H	-1.420431	3.310113	2.519435
H	-3.015183	3.673033	1.868055
H	-2.380252	2.024126	1.808059
H	0.000000	0.000000	1.766380
C	-0.200500	1.708241	-2.759986
H	0.252771	1.800162	-3.750467
H	-1.273956	1.865918	-2.882367
C	-1.379130	-1.027759	-2.759986
H	-1.685371	-0.681175	-3.750467
H	-0.978955	-2.036237	-2.882367
C	1.579630	-0.680482	-2.759986
H	1.432601	-1.118987	-3.750467
H	2.252910	0.170319	-2.882367

15c, neutral form
Stoichiometry: $C_{60}H_{78}O_{18}P_4Pt$
Charge: 0
Multiplicity: 1
Point group: C_3
DLPNO-CCSD(T), Hartree: -5163.9842853
Gibbs in HMPA, Hartree: -5162.8477157
Nuclear coordinates, Å:

C	2.397409	1.384145	2.468350
H	2.079354	2.425734	2.526366
H	3.461997	1.365870	2.690671
C	0.000000	-2.768290	2.468350
H	-0.548120	-3.681112	2.690671
H	1.061070	-3.013641	2.526366
C	-2.397409	1.384145	2.468350
H	-3.140425	0.587907	2.526366
H	-2.913876	2.315242	2.690671
C	1.627244	0.569679	3.510800
H	2.186768	-0.326948	3.773793
H	1.512112	1.159974	4.426318
C	-0.320265	-1.694074	3.510800
H	-1.376529	-1.730323	3.773793
H	0.248511	-1.889514	4.426318
C	-1.306979	1.124395	3.510800
H	-0.810239	2.057271	3.773793
H	-1.760623	0.729540	4.426318
P	2.122437	0.971286	0.627167
P	-0.220060	-2.323727	0.627167
P	-1.902377	1.352441	0.627167
Pt	0.000000	0.000000	0.651673
P	0.000000	0.000000	2.864243
C	-1.768804	-3.268759	0.164394
C	-1.913190	-4.015926	-1.021746
C	-2.930107	-3.154187	0.938639
C	-3.101973	-4.645348	-1.372062
C	-4.137955	-3.771474	0.607254
C	-4.212478	-4.520831	-0.551745
H	-3.193938	-5.227985	-2.276249
H	-5.002383	-3.645599	1.237179
C	3.715231	0.102551	0.164394
C	4.434489	0.351091	-1.021746
C	4.196660	-0.960454	0.938639
C	5.573976	-0.363713	-1.372062
C	5.335170	-1.697837	0.607254
C	6.021393	-1.387697	-0.551745
H	6.124536	-0.152039	-2.276249
H	5.658373	-2.509391	1.237179
C	-1.946427	3.166209	0.164394
C	-2.521298	3.664834	-1.021746
C	-1.266553	4.114641	0.938639
C	-2.472003	5.009062	-1.372062
C	-1.197215	5.469311	0.607254
C	-1.808915	5.908528	-0.551745

H	-2.930598	5.380024	-2.276249
H	-0.655990	6.154990	1.237179
C	2.399558	2.677108	-0.077324
C	3.310550	3.624023	0.419392
C	1.649684	3.083113	-1.192429
C	3.353307	4.935368	-0.041904
C	1.681134	4.391735	-1.683072
C	2.512963	5.316888	-1.078861
H	4.039681	5.666501	0.357756
H	1.053987	4.670680	-2.513397
C	1.118664	-3.416632	-0.077324
C	1.483221	-4.679032	0.419392
C	1.845213	-2.970225	-1.192429
C	2.597501	-5.371733	-0.041904
C	2.962787	-3.651772	-1.683072
C	3.348078	-4.834734	-1.078861
H	2.887493	-6.331716	0.357756
H	3.517934	-3.248120	-2.513397
C	-3.518222	0.739525	-0.077324
C	-4.793771	1.055009	0.419392
C	-3.494896	-0.112889	-1.192429
C	-5.950808	0.436365	-0.041904
C	-4.643921	-0.739963	-1.683072
C	-5.861041	-0.482154	-1.078861
H	-6.927174	0.665216	0.357756
H	-4.571921	-1.422560	-2.513397
O	0.928731	2.134929	-1.807790
O	1.384537	-1.871769	-1.807790
O	-2.313268	-0.263160	-1.807790
O	4.187255	3.190487	1.357389
O	0.669416	-5.221513	1.357389
O	-4.856670	2.031025	1.357389
O	-0.660173	3.652736	2.058888
O	-2.833276	-2.398094	2.058888
O	3.493448	-1.254642	2.058888
O	-3.106810	2.763810	-1.840623
O	-0.840125	-4.072481	-1.840623
O	3.946934	1.308671	-1.840623
O	-1.789219	7.199149	-0.977456
O	-5.340036	-5.149083	-0.977456
O	7.129255	-2.050065	-0.977456
O	2.591628	6.618904	-1.453595
O	4.436325	-5.553868	-1.453595
O	-7.027953	-1.065036	-1.453595
C	-1.163856	8.149814	-0.151166
H	-1.281389	9.110788	-0.649071
H	-1.636774	8.197449	0.835329
H	-0.096291	7.938998	-0.026362
C	-6.476018	-5.082835	-0.151166
H	-7.249480	-5.665109	-0.649071
H	-6.280812	-5.516213	0.835329
H	-6.827229	-4.052890	-0.026362
C	7.639873	-3.066978	-0.151166
H	8.530869	-3.445679	-0.649071
H	7.917586	-2.681236	0.835329
H	6.923520	-3.886108	-0.026362
C	0.118736	4.540292	2.819323
H	0.939143	4.960262	2.228885
H	-0.483986	5.352279	3.240333
H	0.535293	3.954712	3.637969
C	-3.991377	-2.167318	2.819323
H	-4.765284	-1.666809	2.228885
H	-4.393217	-3.095284	3.240333
H	-3.692527	-1.513779	3.637969
C	3.872640	-2.372975	2.819323
H	3.826141	-3.293452	2.228885
H	4.877203	-2.256996	3.240333
H	3.157234	-2.440933	3.637969
C	-6.107600	2.387572	1.882962
H	-6.780609	2.764595	1.106036
H	-6.590391	1.547629	2.394135
H	-5.916584	3.180858	2.603908
C	5.121498	4.095551	1.882962
H	5.784514	4.489882	1.106036
H	4.635481	4.933631	2.394135
H	5.712996	3.533483	2.603908
C	0.986102	-6.483123	1.882962
H	0.203588	-6.714341	2.603908
H	0.996095	-7.254477	1.106036
H	1.954909	-6.481260	2.394135
C	-6.998470	-1.944745	-2.551763
H	-6.662801	-1.437682	-3.462537
H	-6.352327	-2.808234	-2.364649
H	-8.022146	-2.287370	-2.693503
C	1.815036	7.033225	-2.551763
H	0.744161	6.905393	-2.364649
H	2.030152	8.091068	-2.693503
H	2.086331	6.488996	-3.462537
C	5.183434	-5.088480	-2.551763
H	5.608165	-4.097159	-2.364649
H	5.991994	-5.803697	-2.693503
H	4.576470	-5.051313	-3.462537
C	-3.785300	3.211210	-2.982401
H	-4.580766	3.919841	-2.729937
H	-3.106616	3.680847	-3.703797
H	-4.227998	2.324083	-3.432814
C	-0.888340	-4.883772	-2.982401
H	-1.104299	-5.926980	-2.729937
H	-1.634398	-4.530832	-3.703797
H	0.101284	-4.823595	-3.432814
C	4.673640	1.672561	-2.982401
H	5.685065	2.007139	-2.729937
H	4.741015	0.849985	-3.703797
H	4.126714	2.499512	-3.432814
C	-2.209543	-1.102479	-2.926500
H	-2.567540	-2.112768	-2.714272
H	-2.759881	-0.694857	-3.783416
H	-1.146954	-1.153470	-3.156254
C	0.149997	2.464760	-2.926500
H	-0.545941	3.279939	-2.714272
H	0.778177	2.737556	-3.783416
H	-0.425458	1.570026	-3.156254
C	2.059546	-1.362281	-2.926500
H	3.113481	-1.167171	-2.714272
H	1.981704	-2.042699	-3.783416
H	1.572411	-0.416556	-3.156254

15c, protonated form
Stoichiometry: $C_{60}H_{79}O_{18}P_4Pt^+$
Charge: +1
Multiplicity: 1
Point group: C_3
DLPNO-CCSD(T), Hartree: -5164.4972348
Gibbs in HMPA, Hartree: -5163.3612960
Nuclear coordinates, Å:

C	-1.584802	-2.248133	2.547436
H	-0.839181	-3.028542	2.707758
H	-2.554431	-2.689045	2.757868
C	-1.154539	2.496546	2.547436
H	-1.051566	3.556725	2.757868
H	-2.203204	2.241023	2.707758
C	2.739341	-0.248413	2.547436
H	3.042385	0.787519	2.707758
H	3.605997	-0.867680	2.757868
C	-1.315693	-1.081069	3.482779
H	-2.203802	-0.460154	3.580683
H	-1.051304	-1.440208	4.481683
C	-0.278387	1.679958	3.482779
H	0.703396	2.138625	3.580683
H	-0.721605	1.630560	4.481683
C	1.594080	-0.598889	3.482779
H	1.500406	-1.678472	3.580683
H	1.772909	-0.190352	4.481683
P	-1.447922	-1.899503	0.697237
P	-0.921057	2.203689	0.697237
P	2.368979	-0.304185	0.697237
Pt	0.000000	0.000000	0.559732
P	0.000000	0.000000	2.821142
H	0.000000	0.000000	-1.030252
C	0.000000	3.700407	0.107147
C	-0.325676	4.388241	-1.071593
C	1.100087	4.204524	0.825189
C	0.320467	5.565820	-1.454806
C	1.767267	5.360774	0.454086
C	1.361284	6.050092	-0.679268
H	0.023902	6.073283	-2.358087
H	2.601990	5.748201	1.016997
C	-3.204647	-1.850204	0.107147
C	-3.637490	-2.476164	-1.071593
C	-4.191268	-1.149559	0.825189
C	-4.980375	-2.505377	-1.454806
C	-5.526200	-1.149889	0.454086
C	-5.920176	-1.846140	-0.679268
H	-5.271568	-3.015942	-2.358087
H	-6.279083	-0.620712	1.016997
C	3.204647	-1.850204	0.107147
C	3.963166	-1.912077	-1.071593
C	3.091182	-3.054965	0.825189
C	4.659907	-3.060443	-1.454806
C	3.758933	-4.210886	0.454086
C	4.558892	-4.203953	-0.679268
H	5.247666	-3.057341	-2.358087
H	3.677094	-5.127490	1.016997
C	-0.886034	-3.562671	0.118406

C	-1.380321	-4.768290	0.653884
C	0.028055	-3.689075	-0.938691
C	-0.873286	-6.005044	0.281877
C	0.547508	-4.922913	-1.332528
C	0.113502	-6.073781	-0.694227
H	-1.232214	-6.926878	0.713109
H	1.281856	-4.971491	-2.119233
C	-2.642346	2.548663	0.118406
C	-3.439299	3.579538	0.653884
C	-3.208860	1.820241	-0.938691
C	-4.763878	3.758810	0.281877
C	-4.537122	1.987300	-1.332528
C	-5.316799	2.938595	-0.694227
H	-5.382746	4.530568	0.713109
H	-4.946366	1.375626	-2.119233
C	3.528380	1.014007	0.118406
C	4.819621	1.188752	0.653884
C	3.180805	1.868834	-0.938691
C	5.637164	2.246234	0.281877
C	3.989613	2.935613	-1.332528
C	5.203297	3.135186	-0.694227
H	6.614960	2.396311	0.713109
H	3.664510	3.595865	-2.119233
O	0.347669	-2.562385	-1.588858
O	-2.392925	0.980103	-1.588858
O	2.045256	1.582283	-1.588858
O	-2.403424	-4.670129	1.531200
O	-2.842738	4.416491	1.531200
O	5.246162	0.253638	1.531200
O	2.287746	-3.029752	1.912206
O	1.479969	3.496122	1.912206
O	-3.767715	-0.466370	1.912206
O	3.959419	-0.820208	-1.861544
O	-1.269388	3.839061	-1.861544
O	-2.690031	-3.018853	-1.861544
O	5.175180	-5.370242	-0.968649
O	2.063176	7.166958	-0.968649
O	-7.238356	-1.796716	-0.968649
O	0.574415	-7.309043	-0.972504
O	-6.617024	3.157063	-0.972504
O	6.042609	4.151980	-0.972504
C	6.052055	-5.405372	-2.071624
H	6.471157	-6.408850	-2.093711
H	5.524221	-5.217155	-3.012084
H	6.862791	-4.679364	-1.960477
C	1.655161	7.943920	-2.071624
H	2.314649	8.808612	-2.093711
H	1.756078	7.392693	-3.012084
H	0.621052	8.283034	-1.960477
C	-7.707217	-2.538548	-2.071624
H	-8.785806	-2.399761	-2.093711
H	-7.280299	-2.175539	-3.012084
H	-7.483843	-3.603670	-1.960477
C	2.075383	-4.235551	2.613410
H	1.616953	-4.991468	1.970564
H	3.005802	-4.623362	3.037569
H	1.391405	-3.998342	3.427122
C	2.630403	3.915110	2.613410
H	3.514261	3.896057	1.970564
H	2.501048	4.914782	3.037569
H	2.766963	3.204162	3.427122
C	-4.705786	0.320441	2.613410
H	-5.131215	1.095411	1.970564
H	-5.506850	-0.291419	3.037569
H	-4.158368	0.794179	3.427122
C	6.542235	0.365430	2.072022
H	7.305750	0.339303	1.289540
H	6.658363	1.282070	2.657915
H	6.670122	-0.495720	2.724812
C	-2.954645	-5.848457	2.072022
H	-3.359030	-6.496616	1.289540
H	-2.218877	-6.407346	2.657915
H	-3.764367	-5.528635	2.724812
C	-3.587589	5.483026	2.072022
H	-2.905754	6.024355	2.724812
H	-3.946720	6.157314	1.289540
H	-4.439486	5.125277	2.657915
C	5.646108	5.101605	-1.936809
H	5.525426	4.643558	-2.923522
H	4.715075	5.599379	-1.648973
H	6.447025	5.836186	-1.982509
C	1.595066	-7.440475	-1.936809
H	2.491667	-6.883064	-1.648973
H	1.830773	-8.501380	-1.982509
H	1.258726	-7.106939	-2.923522
C	-7.241173	2.338870	-1.936809
H	-7.206742	1.283685	-1.648973
H	-8.277797	2.665194	-1.982509
H	-6.784152	2.463380	-2.923522
C	4.843482	-0.748958	-2.953091
H	5.880138	-0.909584	-2.643477
H	4.582382	-1.469411	-3.735308
H	4.745155	0.259505	-3.350534
C	-1.773124	4.569057	-2.953091
H	-2.152346	5.547141	-2.643477
H	-1.018643	4.703165	-3.735308
H	-2.597316	3.979672	-3.350534
C	-3.070358	-3.820099	-2.953091
H	-3.727791	-4.637557	-2.643477
H	-3.563738	-3.233753	-3.735308
H	-2.147839	-4.239177	-3.350534
C	1.726533	2.271571	-2.770811
H	1.591333	3.342018	-2.597093
H	2.495064	2.120891	-3.536690
H	0.782014	1.850031	-3.109368
C	1.103972	-2.631007	-2.770811
H	2.098606	-3.049144	-2.597093
H	0.589214	-3.221235	-3.536690
H	1.211167	-1.602260	-3.109368
C	-2.830505	0.359436	-2.770811
H	-3.689939	-0.292874	-2.597093
H	-3.084278	1.100343	-3.536690
H	-1.993181	-0.247771	-3.109368

15d, neutral form
Stoichiometry: $C_{66}H_{96}N_6O_{12}P_4Pt$
Charge: 0
Multiplicity: 1
Point group: C_3
DLPNO-CCSD(T), Hartree: -5280.2711179
Gibbs in HMPA, Hartree: -5278.9109969
Nuclear coordinates, Å:

C	-2.096793	1.795046	2.556243
H	-2.959068	1.132354	2.646255
H	-2.445413	2.802292	2.772091
C	2.602952	0.918353	2.556243
H	3.649563	0.716644	2.772091
H	2.460182	1.996451	2.646255
C	-0.506159	-2.713399	2.556243
H	0.498886	-3.128805	2.646255
H	-1.204150	-3.518936	2.772091
C	-1.037700	1.372867	3.575106
H	-0.358314	2.200543	3.776118
H	-1.521262	1.109183	4.522276
C	1.707788	0.212241	3.575106
H	2.084883	-0.789962	3.776118
H	1.721212	0.762860	4.522276
C	-0.670088	-1.585108	3.575106
H	-1.726569	-1.410580	3.776118
H	-0.199949	-1.872043	4.522276
P	-1.663344	1.652137	0.703436
P	2.262465	0.614429	0.703436
P	-0.599121	-2.266567	0.703436
Pt	0.000000	0.000000	0.716752
P	0.000000	0.000000	2.928254
C	3.704236	-0.469221	0.204485
C	4.452869	-0.291298	-0.970539
C	4.035736	-1.618070	0.936617
C	5.490192	-1.139996	-1.347968
C	5.056575	-2.490911	0.574121
C	5.799884	-2.266064	-0.583105
H	6.036124	-0.932477	-2.253104
H	5.256945	-3.352542	1.187208
C	-1.445761	3.442573	0.204485
C	-1.974163	4.001947	-0.970539
C	-0.616578	4.304085	0.936617
C	-1.757830	5.324644	-1.347968
C	-0.371096	5.624578	0.574121
C	-0.937473	6.155879	-0.583105
H	-2.210514	5.693675	-2.253104
H	0.274914	6.228918	1.187208
C	-2.258475	-2.973352	0.204485
C	-2.478706	-3.710649	-0.970539
C	-3.419158	-2.686015	0.936617
C	-3.732362	-4.184647	-1.347968
C	-4.685480	-3.133667	0.574121
C	-4.862411	-3.889815	-0.583105
H	-3.825611	-4.761199	-2.253104
H	-5.531859	-2.876377	1.187208
C	-3.384321	1.307251	0.072884
C	-4.562455	1.871740	0.576341
C	-3.562594	0.428380	-1.009636
C	-5.832511	1.467090	0.171093
C	-4.819336	0.008276	-1.437041

C	-5.973754	0.484762	-0.814424
H	-6.702811	1.928027	0.607132
H	-4.889086	-0.700011	-2.244901
C	2.824273	2.277282	0.072884
C	3.902202	3.015332	0.576341
C	2.152285	2.871107	-1.009636
C	4.186793	4.317557	0.171093
C	2.416835	4.169530	-1.437041
C	3.406693	4.931042	-0.814424
H	5.021126	4.840791	0.607132
H	1.838316	4.584078	-2.244901
C	0.560048	-3.584533	0.072884
C	0.660253	-4.887072	0.576341
C	1.410309	-3.299487	-1.009636
C	1.645718	-5.784648	0.171093
C	2.402501	-4.177805	-1.437041
C	2.567061	-5.415804	-0.814424
H	1.681685	-6.768819	0.607132
H	3.050770	-3.884067	-2.244901
O	-2.447961	0.048418	-1.651098
O	1.265911	2.095787	-1.651098
O	1.182049	-2.144205	-1.651098
O	-4.415776	2.882581	1.470972
O	4.704276	2.382883	1.470972
O	-0.288501	-5.265464	1.470972
O	-3.248568	-1.935682	2.053020
O	3.300634	-1.845501	2.053020
O	-0.052066	3.781183	2.053020
O	-1.403982	-3.919180	-1.765795
O	4.096100	0.743706	-1.765795
O	-2.692118	3.175474	-1.765795
C	-4.384263	-1.489670	2.747735
H	-5.036828	-0.890696	2.104969
H	-4.952685	-2.322347	3.176889
H	-4.016367	-0.864267	3.560509
C	3.482223	-3.052048	2.747735
H	3.289779	-3.916673	2.104969
H	4.487554	-3.127977	3.176889
H	2.756660	-3.046142	3.560509
C	0.902040	4.541718	2.747735
H	1.747049	4.807369	2.104969
H	0.465131	5.450325	3.176889
H	1.259706	3.910409	3.560509
C	-0.236987	-6.550666	2.025021
H	-0.336806	-7.328852	1.260446
H	0.689790	-6.719657	2.585053
H	-1.081628	-6.617890	2.709352
C	-5.554550	3.480569	2.025021
H	-6.178569	3.956109	1.260446
H	-6.164288	2.762452	2.585053
H	-5.190447	4.245662	2.709352
C	5.791536	3.070096	2.025021
H	6.272075	2.372228	2.709352
H	6.515375	3.372743	1.260446
H	5.474498	3.957204	2.585053
C	-1.522806	-4.732219	-2.898358
H	-1.875012	-5.737814	-2.643760
H	-2.196233	-4.298224	-3.647288
H	-0.520869	-4.804247	-3.319470
C	4.859625	1.047321	-2.898358
H	5.906598	1.245099	-2.643760
H	4.820487	0.247118	-3.647288
H	4.421034	1.951038	-3.319470
C	-3.336819	3.684898	-2.898358
H	-4.031587	4.492715	-2.643760
H	-2.624254	4.051105	-3.647288
H	-3.900166	2.853209	-3.319470
C	1.944023	-1.804922	-2.776962
H	3.014234	-1.781031	-2.555827
H	1.760889	-2.499460	-3.606414
H	1.623045	-0.802284	-3.054620
C	-2.535120	-0.781112	-2.776962
H	-3.049535	-1.719887	-2.555827
H	-3.045040	-0.275245	-3.606414
H	-1.506321	-1.004457	-3.054620
C	0.591097	2.586034	-2.776962
H	0.035301	3.500919	-2.555827
H	1.284151	2.774705	-3.606414
H	-0.116725	1.806740	-3.054620
N	-0.661293	7.456041	-0.986906
C	0.000000	8.335453	-0.057746
C	-1.535025	8.074134	-1.950860
H	-0.576468	8.505474	0.863949
H	0.977743	7.936741	0.222391
H	0.166417	9.298862	-0.538406
H	-1.518990	7.534513	-2.900711
H	-2.580120	8.130001	-1.612465
H	-1.185288	9.086440	-2.149574
N	-6.126474	-4.300717	-0.986906
C	-6.224893	-5.366438	-1.950860
C	-7.218714	-4.167726	-0.057746
H	-5.765584	-5.082740	-2.900711
H	-5.750728	-6.299450	-1.612465
H	-7.276444	-5.569710	-2.149574
H	-7.077722	-4.751973	0.863949
H	-7.362291	-3.121620	0.222391
H	-8.136260	-4.505310	-0.538406
N	6.787767	-3.155324	-0.986906
C	7.218714	-4.167726	-0.057746
C	7.759918	-2.707697	-1.950860
H	7.654190	-3.753501	0.863949
H	6.384548	-4.815121	0.222391
H	7.969842	-4.793553	-0.538406
H	7.284574	-2.451772	-2.900711
H	8.330847	-1.830552	-1.612465
H	8.461732	-3.516730	-2.149574
N	3.616324	6.253703	-1.167295
C	4.844237	6.891239	-0.770982
C	2.963563	6.769362	-2.343618
H	4.949344	6.899020	0.316473
H	5.738635	6.408620	-1.192622
H	4.829339	7.928696	-1.102599
H	3.274701	6.260223	-3.268116
H	1.878448	6.683599	-2.253911
H	3.200718	7.828093	-2.443502
N	-7.224028	0.004977	-1.167295
C	-8.390106	0.749613	-0.770982
C	-7.344221	-0.818160	-2.343618
H	-8.449399	0.836748	0.316473
H	-8.419345	1.765494	-1.192622
H	-9.281122	0.217982	-1.102599
H	-7.058863	-0.294137	-3.268116
H	-6.727390	-1.715016	-2.253911
H	-8.379686	-1.142143	-2.443502
N	3.607703	-6.258680	-1.167295
C	3.545870	-7.640852	-0.770982
C	4.380658	-5.951202	-2.343618
H	3.500055	-7.735768	0.316473
H	2.680710	-8.174113	-1.192622
H	4.451783	-8.146678	-1.102599
H	3.784162	-5.966086	-3.268116
H	4.848942	-4.968583	-2.253911
H	5.178968	-6.685950	-2.443502

15d, protonated form
Stoichiometry: $C_{66}H_{97}N_6O_{12}P_4Pt^+$
Charge: +1
Multiplicity: 1
Point group: C_3
DLPNO-CCSD(T), Hartree: -5280.7964767
Gibbs in HMPA, Hartree: -5279.4287651
Nuclear coordinates, Å:

C	-0.379683	-2.726971	2.591365
H	0.641622	-3.081985	2.736662
H	-1.040098	-3.562225	2.805029
C	-2.171785	1.692301	2.591365
H	-2.564929	2.681863	2.805029
H	-2.989889	0.985331	2.736662
C	2.551468	1.034670	2.591365
H	2.348266	2.096654	2.736662
H	3.605026	0.880362	2.805029
C	-0.660825	-1.570513	3.537262
H	-1.733203	-1.428118	3.653169
H	-0.245163	-1.772727	4.528940
C	-1.029691	1.357548	3.537262
H	-0.370185	2.215057	3.653169
H	-1.412645	1.098681	4.528940
C	1.690516	0.212965	3.537262
H	2.103389	-0.786939	3.653169
H	1.657808	0.674046	4.528940
P	-0.438298	-2.345769	0.743101
P	-1.812346	1.552462	0.743101
P	2.250644	0.793307	0.743101
Pt	0.000000	0.000000	0.615072
P	0.000000	0.000000	2.873256
H	0.000000	0.000000	-0.973010
C	-1.649072	3.304622	0.168278
C	-2.229043	3.791556	-1.016586
C	-0.893497	4.242413	0.887997
C	-2.148071	5.125663	-1.399136
C	-0.787230	5.575978	0.519856
C	-1.420742	6.043431	-0.634690
H	-2.633340	5.442560	-2.306899
H	-0.200012	6.247334	1.121937

C	-2.037351	-3.080449	0.168278
C	-2.169063	-3.826186	-1.016586
C	-3.227289	-2.894997	0.887997
C	-3.364918	-4.423115	-1.399136
C	-4.435324	-3.469750	0.519856
C	-4.523393	-4.252114	-0.634690
H	-3.396725	-5.001819	-2.306899
H	-5.310344	-3.296882	1.121937
C	3.686422	-0.224173	0.168278
C	4.398106	0.034630	-1.016586
C	4.120786	-1.347416	0.887997
C	5.512989	-0.702547	-1.399136
C	5.222554	-2.106228	0.519856
C	5.944135	-1.791317	-0.634690
H	6.030065	-0.440741	-2.306899
H	5.510356	-2.950451	1.121937
C	0.812050	-3.561670	0.139886
C	0.940590	-4.860228	0.658135
C	1.660963	-3.260489	-0.940691
C	1.950554	-5.730868	0.265056
C	2.671998	-4.116836	-1.357091
C	2.865377	-5.348873	-0.724535
H	2.010052	-6.710127	0.708802
H	3.310919	-3.817691	-2.170251
C	-3.490522	1.077579	0.139886
C	-4.679376	1.615539	0.658135
C	-3.654148	0.191808	-0.940691
C	-5.938354	1.176204	0.265056
C	-4.901283	-0.255601	-1.357091
C	-6.064948	0.192947	-0.724535
H	-6.816167	1.614308	0.708802
H	-4.961677	-0.958494	-2.170251
C	2.678472	2.484091	0.139886
C	3.738786	3.244689	0.658135
C	1.993185	3.068681	-0.940691
C	3.987800	4.554664	0.265056
C	2.229285	4.372436	-1.357091
C	3.199571	5.155925	-0.724535
H	4.806115	5.095819	0.708802
H	1.650758	4.776185	-2.170251
O	1.408837	-2.114600	-1.588271
O	-2.535716	-0.162789	-1.588271
O	1.126879	2.277389	-1.588271
O	0.000000	-5.250583	1.551177
O	-4.547138	2.625291	1.551177
O	4.547138	2.625291	1.551177
O	3.400355	-1.662549	1.991626
O	-0.260368	3.776068	1.991626
O	-3.139987	-2.113519	1.991626
O	3.924479	1.020851	-1.805868
O	-2.846322	2.888273	-1.805868
O	-1.078157	-3.909124	-1.805868
C	3.722244	-2.839604	2.694351
H	3.625226	-3.722389	2.056345
H	4.730304	-2.795417	3.117887
H	3.005196	-2.912338	3.511429
C	0.598047	4.643360	2.694351
H	1.411071	5.000732	2.056345
H	0.055750	5.494272	3.117887
H	1.019561	4.058745	3.511429
C	-4.320291	-1.803756	2.694351
H	-5.036296	-1.278343	2.056345
H	-4.786054	-2.698855	3.117887
H	-4.024757	-1.146407	3.511429
C	5.645652	3.320236	2.086179
H	6.346938	3.628099	1.304784
H	5.332461	4.201025	2.656010
H	6.145059	2.622567	2.756021
C	0.052583	-6.549396	2.086179
H	-0.031444	-7.310659	1.304784
H	0.971964	-6.718559	2.656010
H	-0.801319	-6.633061	2.756021
C	-5.698235	3.229160	2.086179
H	-5.343739	4.010493	2.756021
H	-6.315495	3.682560	1.304784
H	-6.304425	2.517534	2.656010
C	4.663314	1.430386	-2.927609
H	5.678864	1.732846	-2.654589
H	4.713971	0.646189	-3.690801
H	4.134261	2.290727	-3.333831
C	-3.570408	3.323356	-2.927609
H	-4.340121	4.051618	-2.654589
H	-2.916602	3.759324	-3.690801
H	-4.050958	2.435011	-3.333831
C	-1.092907	-4.753742	-2.927609
H	-1.338744	-5.784464	-2.654589
H	-1.797369	-4.405514	-3.690801
H	-0.083302	-4.725738	-3.333831
C	0.510298	2.732150	-2.764671
H	-0.097260	3.623759	-2.589754
H	1.250502	2.943283	-3.544574
H	-0.141345	1.921325	-3.085322
C	2.110963	-1.808006	-2.764671
H	3.186897	-1.727650	-2.589754
H	1.923706	-2.554608	-3.544574
H	1.734589	-0.838254	-3.085322
C	-2.621261	-0.924144	-2.764671
H	-3.089637	-1.896109	-2.589754
H	-3.174209	-0.388675	-3.544574
H	-1.593244	-1.083071	-3.085322
N	-1.301601	7.356667	-1.026065
C	-0.708598	8.310627	-0.122538
C	-2.148090	7.857765	-2.079741
H	-1.265691	8.408582	0.818879
H	0.321415	8.035402	0.119752
H	-0.680898	9.286402	-0.603513
H	-1.969589	7.326168	-3.018283
H	-3.216763	7.775802	-1.841156
H	-1.918198	8.907149	-2.252811
N	7.021861	-2.551114	-1.026065
C	7.551514	-3.541649	-0.122538
C	7.879069	-2.068582	-2.079741
H	7.914891	-3.108170	0.818879
H	6.798155	-4.296054	0.119752
H	8.382709	-4.053526	-0.603513
H	7.329442	-1.957370	-3.018283
H	8.342424	-1.102103	-1.841156
H	8.672917	-2.792366	-2.252811
N	-5.720260	-4.805553	-1.026065
C	-6.842915	-4.768978	-0.122538
C	-5.730979	-5.789183	-2.079741
H	-6.649200	-5.300412	0.818879
H	-7.119570	-3.739348	0.119752
H	-7.701811	-5.232876	-0.603513
H	-5.359853	-5.368798	-3.018283
H	-5.125661	-6.673699	-1.841156
H	-6.754719	-6.114783	-2.252811
N	3.384252	6.471808	-1.074043
C	4.545787	7.177137	-0.594799
C	2.705295	6.999351	-2.231076
H	4.574739	7.190914	0.497578
H	5.488426	6.742643	-0.954450
H	4.499231	8.210753	-0.932214
H	3.013403	6.511742	-3.166180
H	1.622160	6.892214	-2.132131
H	2.924361	8.062160	-2.316574
N	3.912624	-6.166752	-1.074043
C	4.708969	-5.842530	-2.231076
C	3.942689	-7.525336	-0.594799
H	4.132632	-5.865554	-3.166180
H	5.157752	-4.850938	-2.132131
H	5.519855	-6.563651	-2.316574
H	3.940145	-7.557297	0.497578
H	3.095087	-8.124438	-0.954450
H	4.861105	-8.001825	-0.932214
N	-7.296876	-0.305056	-1.074043
C	-8.488476	0.348199	-0.594799
C	-7.414263	-1.156822	-2.231076
H	-8.514884	0.366383	0.497578
H	-8.583513	1.381795	-0.954450
H	-9.360336	-0.208928	-0.932214
H	-7.146035	-0.646187	-3.166180
H	-6.779912	-2.041275	-2.132131
H	-8.444216	-1.498509	-2.316574

[Pt(dmpe)$_2$] complex
Stoichiometry: $C_{12}H_{32}P_4Pt$
Charge: 0
Multiplicity: 1
Point group: S_4
DLPNO-CCSD(T), Hartree: -1958.6487046
Gibbs in HMPA, Hartree: -1958.2958520
Nuclear coordinates, Å:

Pt	0.000000	0.000000	0.000000
P	-0.613419	1.457344	1.651393
P	0.613419	-1.457344	1.651393
P	-1.457344	-0.613419	-1.651393
P	1.457344	0.613419	-1.651393
C	-0.029965	-0.760039	3.252925
C	0.029965	0.760039	3.252925
C	0.760039	-0.029965	-3.252925
C	-0.760039	0.029965	-3.252925
C	2.389220	-1.741706	2.062378
H	2.515118	-2.353388	2.960257

H	2.872432	-2.238980	1.220086
H	2.879622	-0.778265	2.205744
C	-2.389220	1.741706	2.062378
H	-2.515118	2.353388	2.960257
H	-2.872432	2.238980	1.220086
H	-2.879622	0.778265	2.205744
C	0.000000	3.188971	1.779868
H	-0.420592	3.776717	0.962718
H	-0.273490	3.654701	2.731025
H	1.085305	3.189850	1.673803
C	0.000000	-3.188971	1.779868
H	0.420592	-3.776717	0.962718
H	0.273490	-3.654701	2.731025
H	-1.085305	-3.189850	1.673803
C	-1.741706	-2.389220	-2.062378
H	-2.238980	-2.872432	-1.220086
H	-2.353388	-2.515118	-2.960257
H	-0.778265	-2.879622	-2.205744
C	-3.188971	0.000000	-1.779868
H	-3.654701	-0.273490	-2.731025
H	-3.776717	-0.420592	-0.962718
H	-3.189850	1.085305	-1.673803
C	1.741706	2.389220	-2.062378
H	2.238980	2.872432	-1.220086
H	2.353388	2.515118	-2.960257
H	0.778265	2.879622	-2.205744
C	3.188971	0.000000	-1.779868
H	3.654701	0.273490	-2.731025
H	3.776717	0.420592	-0.962718
H	3.189850	-1.085305	-1.673803
H	-0.512862	1.183979	4.103655
H	1.068405	1.096203	3.329229
H	-1.068405	-1.096203	3.329229
H	0.512862	-1.183979	4.103655
H	-1.183979	-0.512862	-4.103655
H	-1.096203	1.068405	-3.329229
H	1.096203	-1.068405	-3.329229
H	1.183979	0.512862	-4.103655

$[HPt(dmpe)_2]^+$ complex
Stoichiometry: $C_{12}H_{33}P_4Pt^+$
Charge: +1
Multiplicity: 1
Point group: C_1
DLPNO-CCSD(T), Hartree: -1959.0954858
Gibbs in HMPA, Hartree: -1958.7756067
Nuclear coordinates, Å:

H	0.366838	0.931880	-1.556213
Pt	-0.065154	0.099844	-0.266474
P	1.558252	1.536196	0.779081
P	1.760566	-1.235079	-0.934414
P	-1.139034	-1.128047	1.390832
P	-2.162528	0.699832	-0.925009
C	3.262067	-0.471569	-0.165149
C	3.139063	1.042079	-0.045271
C	-3.399283	-0.254199	0.057492
C	-2.873705	-0.505206	1.460788
C	2.147991	-1.239408	-2.718855
H	3.082336	-1.767620	-2.919210
H	1.336179	-1.723916	-3.261625
H	2.225285	-0.212843	-3.076499
C	1.996024	1.475432	2.557283
H	2.871506	2.094806	2.763577
H	1.160199	1.842189	3.153787
H	2.207044	0.449681	2.859738
C	1.502156	3.335501	0.468654
H	0.668851	3.772413	1.019708
H	2.427829	3.823770	0.780625
H	1.339945	3.512992	-0.594433
C	1.970004	-2.996736	-0.495671
H	1.190771	-3.591626	-0.971972
H	2.944365	-3.360294	-0.828809
H	1.899030	-3.124347	0.584600
C	-1.353053	-2.920934	1.109551
H	-0.387003	-3.421894	1.157279
H	-2.011597	-3.353713	1.864833
H	-1.773874	-3.090052	0.118729
C	-0.614659	-1.075921	3.138165
H	-1.319743	-1.622297	3.767291
H	0.371282	-1.529726	3.242643
H	-0.558511	-0.042129	3.476867
C	-2.641953	2.445533	-0.673350
H	-1.967513	3.085592	-1.242792
H	-3.667744	2.625112	-1.001216
H	-2.545679	2.704029	0.381205
C	-2.660153	0.411323	-2.654526
H	-3.710011	0.669083	-2.807966
H	-2.043133	1.025163	-3.310995
H	-2.496962	-0.635070	-2.909976
H	3.990729	1.461763	0.496262
H	3.127102	1.505332	-1.035886
H	3.360569	-0.922887	0.826887
H	4.153930	-0.751798	-0.731671
H	-3.502091	-1.211464	2.009068
H	-2.848522	0.424392	2.035761
H	-3.560263	-1.198243	-0.469462
H	-4.355001	0.274971	0.072815

dmpe free ligand
Stoichiometry: $C_6H_{16}P_2$
Charge: 0
Multiplicity: 1
Point group: C_i
DLPNO-CCSD(T), Hartree: -919.6571994
Gibbs in HMPA, Hartree: -919.4919393
Nuclear coordinates, Å:

P	2.174171	0.634707	0.001969
P	-2.174171	-0.634707	-0.001969
C	-0.586314	0.228676	-0.428071
C	0.586314	-0.228676	0.428071
C	2.599733	-0.231476	-1.572030
H	2.543968	-1.319138	-1.473876
H	1.926556	0.089492	-2.368209
C	3.306272	-0.264798	1.149192
H	3.209027	-1.350325	1.062231
H	4.338556	0.015529	0.932976
H	3.091424	0.029039	2.178033
C	-3.306272	0.264798	-1.149192
H	-3.209027	1.350325	-1.062231
H	-3.091424	-0.029039	-2.178033
C	-2.599733	0.231476	1.572030
H	-2.543968	1.319138	1.473876
H	-3.612815	-0.045469	1.869013
H	-1.926556	-0.089492	2.368209
H	-0.719260	1.313499	-0.350879
H	-0.393628	0.005310	-1.482098
H	0.719260	-1.313499	0.350879
H	0.393628	-0.005310	1.482098
H	-4.338556	-0.015529	-0.932976
H	3.612815	0.045469	-1.869013

15a, free ligand
Stoichiometry: $C_{12}H_{30}P_4$
Charge: 0
Multiplicity: 1
Point group: C_3
DLPNO-CCSD(T), Hartree: -1838.1171220
Gibbs in HMPA, Hartree: -1837.7870205
Nuclear coordinates, Å:

C	0.484549	2.760910	0.659087
H	0.350192	2.863335	1.740303
H	1.552012	2.584872	0.486322
C	-2.633293	-0.960823	0.659087
H	-3.014571	0.051646	0.486322
H	-2.654817	-1.128393	1.740303
C	2.148743	-1.800087	0.659087
H	2.304625	-1.734942	1.740303
H	1.462559	-2.636518	0.486322
C	-0.356292	1.599392	0.147210
H	-1.415726	1.819313	0.308598
H	-0.221402	1.459518	-0.931223
C	-1.206969	-1.108254	0.147210
H	-0.867708	-2.135711	0.308598
H	-1.153278	-0.921498	-0.931223
C	1.563260	-0.491139	0.147210
H	2.283434	0.316398	0.308598
H	1.374680	-0.538019	-0.931223
P	0.000000	4.388079	-0.092706
P	-3.800188	-2.194040	-0.092706
P	3.800188	-2.194040	-0.092706
P	0.000000	0.000000	1.023971
C	-4.028961	-1.449349	-1.766946
H	-4.834641	-1.973569	-2.284011
H	-4.274994	-0.384960	-1.716544
H	-3.121630	-1.577780	-2.358861
C	0.759307	4.213857	-1.766946
H	0.708160	5.173706	-2.284011
H	1.804112	3.894733	-1.716544
H	0.194417	3.492301	-2.358861
C	3.269654	-2.764508	-1.766946
H	4.126481	-3.200138	-2.284011
H	2.470882	-3.509773	-1.716544
H	2.927213	-1.914521	-2.358861
C	1.274543	5.462674	0.698646

H	2.285574	5.063255	0.581457
H	1.235184	6.458514	0.254028
H	1.055040	5.564846	1.762694
C	-5.368086	-1.627550	0.698646
H	-5.527695	-0.552262	0.581457
H	-6.210829	-2.159556	0.254028
H	-5.346818	-1.868732	1.762694
C	4.093543	-3.835124	0.698646
H	3.242121	-4.510993	0.581457
H	4.975645	-4.298957	0.254028
H	4.291778	-3.696114	1.762694

15b, free ligand
Stoichiometry: $C_{24}H_{54}P_4$
Charge: 0
Multiplicity: 1
Point group: C_3
DLPNO-CCSD(T), Hartree: -2308.9760270
Gibbs in HMPA, Hartree: -2308.3264726
Nuclear coordinates, Å:

C	-2.670919	0.848984	0.386499
H	-2.882402	0.596145	1.429737
H	-2.267443	1.865174	0.389031
C	0.600218	-2.737576	0.386499
H	-0.481567	-2.896250	0.389031
H	0.924924	-2.794306	1.429737
C	2.070701	1.888592	0.386499
H	1.957478	2.198161	1.429737
H	2.749010	1.031076	0.389031
P	-4.236250	0.957699	-0.614407
P	1.288733	-4.147550	-0.614407
P	2.947517	3.189851	-0.614407
P	0.000000	0.000000	0.687305
C	-5.073107	-0.693615	-0.298799
H	-6.130988	-0.456625	-0.453890
C	3.137242	-4.046632	-0.298799
H	3.460943	-5.081279	-0.453890
C	1.935865	4.740247	-0.298799
H	2.670045	5.537904	-0.453890
C	4.435992	3.495663	0.480491
H	4.089921	3.779939	1.480658
C	0.809337	-5.589513	0.480491
H	1.228563	-5.431945	1.480658
C	-5.245329	2.093850	0.480491
H	-5.318484	1.652006	1.480658
C	-0.708287	-5.682597	0.598456
H	-1.139913	-4.832382	1.127566
H	-1.174228	-5.738363	-0.389517
H	-0.989237	-6.585587	1.147602
C	5.275417	2.227904	0.598456
H	4.754922	1.428997	1.127566
H	5.556682	1.852270	-0.389517
H	6.197904	2.436089	1.147602
C	-4.567130	3.454693	0.598456
H	-3.615009	3.403385	1.127566
H	-4.382454	3.886093	-0.389517
H	-5.208667	4.149498	1.147602
C	3.560541	-3.624522	1.100447
H	3.098932	-4.236663	1.877703
H	4.645065	-3.714421	1.213496
H	3.302177	-2.582096	1.301221
C	-4.919199	-1.271258	1.100447
H	-5.218524	-0.565422	1.877703
H	-5.539315	-2.165534	1.213496
H	-3.887249	-1.568721	1.301221
C	1.358658	4.895780	1.100447
H	2.119592	4.802085	1.877703
H	0.894251	5.879955	1.213496
H	0.585072	4.150817	1.301221
C	3.822627	-3.204897	-1.371750
H	3.554224	-2.149241	-1.294084
H	4.910102	-3.272136	-1.273994
H	3.549109	-3.539349	-2.373748
C	-4.686836	-1.708043	-1.371750
H	-3.638409	-2.003428	-1.294084
H	-5.288804	-2.616205	-1.273994
H	-4.839721	-1.303944	-2.373748
C	0.864209	4.912941	-1.371750
H	0.084185	4.152669	-1.294084
H	0.378702	5.888341	-1.273994
H	1.290612	4.843293	-2.373748
C	1.363792	-6.889296	-0.091657
H	2.453934	-6.900997	-0.134135
H	1.052607	-7.737579	0.524435
H	0.988554	-7.057608	-1.104569
C	5.284410	4.625726	-0.091657
H	4.749472	5.575667	-0.134135
H	6.174637	4.780374	0.524435
H	5.617791	4.384917	-1.104569
C	-6.648201	2.263570	-0.091657
H	-7.203406	1.325330	-0.134135
H	-7.227243	2.957205	0.524435
H	-6.606345	2.672691	-1.104569
C	0.725347	1.468653	-0.192574
H	0.811229	1.253475	-1.263336
H	0.000000	2.279415	-0.091277
C	0.909217	-1.362495	-0.192574
H	0.679927	-1.329282	-1.263336
H	1.974031	-1.139707	-0.091277
C	-1.634564	-0.106158	-0.192574
H	-1.491156	0.075807	-1.263336
H	-1.974031	-1.139707	-0.091277

15c, free ligand
Stoichiometry: $C_{60}H_{78}O_{18}P_4$
Charge: 0
Multiplicity: 1
Point group: C_3
DLPNO-CCSD(T), Hartree: -5044.6825227
Gibbs in HMPA, Hartree: -5043.5673004
Nuclear coordinates, Å:

C	-0.474320	-2.745508	-0.312748
H	-0.422693	-2.801578	0.772593
H	-1.520021	-2.599380	-0.584742
C	2.614840	0.961981	-0.312748
H	3.011139	-0.016687	-0.584742
H	2.637584	1.034726	0.772593
C	-2.140520	1.783527	-0.312748
H	-2.214891	1.766852	0.772593
H	-1.491119	2.616067	-0.584742
C	0.380038	-1.598193	-0.838021
H	1.437603	-1.803390	-0.647221
H	0.266482	-1.483133	-1.921516
C	1.194057	1.128219	-0.838021
H	0.842980	2.146695	-0.647221
H	1.151189	0.972347	-1.921516
C	-1.574095	0.469974	-0.838021
H	-2.280583	-0.343306	-0.647221
H	-1.417672	0.510786	-1.921516
P	0.069143	-4.331545	-1.118541
P	3.716656	2.225652	-1.118541
P	-3.785800	2.105893	-1.118541
P	0.000000	0.000000	0.025180
C	5.381595	2.106553	-0.322988
C	6.296415	3.096461	-0.697305
C	5.880001	1.083443	0.498692
C	7.623670	3.111714	-0.267027
C	7.198530	1.069873	0.936938
C	8.062420	2.087124	0.556533
H	8.287909	3.903859	-0.572069
H	7.581561	0.287699	1.574677
C	-0.866469	-5.713874	-0.322988
C	-0.466593	-7.001085	-0.697305
C	-2.001711	-5.633952	0.498692
C	-1.117011	-8.158149	-0.267027
C	-2.672728	-6.769047	0.936938
C	-2.223708	-8.025823	0.556533
H	-0.763113	-9.129469	-0.572069
H	-3.541626	-6.709674	1.574677
C	-4.515125	3.607321	-0.322988
C	-5.829821	3.904624	-0.697305
C	-3.878290	4.550508	0.498692
C	-6.506659	5.046434	-0.267027
C	-4.525802	5.699173	0.936938
C	-5.838713	5.938699	0.556533
H	-7.524796	5.225610	-0.572069
H	-4.039935	6.421975	1.574677
C	1.738847	-4.508755	-0.350877
C	1.971335	-4.615356	1.030666
C	2.864752	-4.509547	-1.180133
C	3.252745	-4.717282	1.556926
C	4.164688	-4.609695	-0.676114
C	4.341769	-4.712198	0.694709
H	3.438275	-4.800810	2.617227
H	5.011367	-4.599614	-1.342570
C	3.035272	3.760263	-0.350877
C	3.011348	4.014904	1.030666
C	2.473006	4.735722	-1.180133
C	2.458914	5.175601	1.556926
C	1.909769	5.911573	-0.676114
C	1.909999	6.116181	0.694709
H	2.438485	5.378038	2.617227
H	1.477699	6.639778	-1.342570
C	-4.774120	0.748491	-0.350877

```
C   -4.982683    0.600452    1.030666
C   -5.337759   -0.226175   -1.180133
C   -5.711659   -0.458318    1.556926
C   -6.074457   -1.301878   -0.676114
C   -6.251768   -1.403983    0.694709
H   -5.876761   -0.577229    2.617227
H   -6.489066   -2.040164   -1.342570
O    2.632455   -4.401573   -2.506465
O    2.495647    4.480559   -2.506465
O   -5.128102   -0.078986   -2.506465
O    0.877420   -4.607842    1.820288
O    3.551799    3.063789    1.820288
O   -4.429218    1.544054    1.820288
O   -2.595351    4.294516    0.840656
O    5.016835    0.100382    0.840656
O   -2.421484   -4.394898    0.840656
O   -6.415303    2.997174   -1.513840
O    5.803280    4.057228   -1.513840
O    0.612023   -7.054402   -1.513840
O    1.381560    7.213478    1.290267
O    5.556275   -4.803205    1.290267
O   -6.937835   -2.410273    1.290267
O   -6.390335    7.080180    1.039371
O   -2.936449   -9.074283    1.039371
O    9.326784    1.994102    1.039371
C   -1.901226    5.226461    1.630385
H   -1.849116    6.207027    1.146347
H   -2.361028    5.342364    2.617585
H   -0.892475    4.834510    1.746358
C    5.476861   -0.966721    1.630385
H    6.300001   -1.502133    1.146347
H    5.807137   -0.626472    2.617585
H    4.633046   -1.644349    1.746358
C   -3.575635   -4.259740    1.630385
H   -4.450885   -4.704895    1.146347
H   -3.446109   -4.715892    2.617585
H   -3.740571   -3.190161    1.746358
C   -4.616942    1.460131    3.209960
H   -5.677478    1.507647    3.476725
H   -4.182431    0.543401    3.621613
H   -4.103204    2.320886    3.633739
C    1.043961   -4.728455    3.209960
H    1.533078   -5.670664    3.476725
H    1.620617   -3.893792    3.621613
H    0.041656   -4.713922    3.633739
C    3.572982    3.268324    3.209960
H    4.061548    2.393036    3.633739
H    4.144400    4.163017    3.476725
H    2.561814    3.350392    3.621613
C   -7.714181    3.234873   -1.985274
H   -8.442956    3.266559   -1.168178
H   -7.774065    4.166943   -2.557339
H   -7.953058    2.399397   -2.640812
C    6.658573    5.063240   -1.985274
H    7.050401    5.678535   -1.168178
H    7.495711    4.649066   -2.557339
H    6.054468    5.687851   -2.640812
C    1.055608   -8.298114   -1.985274
H    1.392555   -8.945094   -1.168178
H    0.278354   -8.816009   -2.557339
H    1.898590   -8.087249   -2.640812
C   -5.615528   -1.056494   -3.387561
H   -5.188365   -2.042646   -3.177403
H   -6.708692   -1.124534   -3.357387
H   -5.307876   -0.741737   -4.382995
C    3.722715   -4.334942   -3.387561
H    4.363166   -3.471933   -3.177403
H    4.328221   -5.247630   -3.357387
H    3.296301   -4.225887   -4.382995
C    1.892813    5.391437   -3.387561
H    0.825199    5.514579   -3.177403
H    2.380471    6.372165   -3.357387
H    2.011575    4.967624   -4.382995
C   -2.527675  -10.368697    0.678608
H   -2.574529  -10.520305   -0.405098
H   -1.511191  -10.583243    1.025566
H   -3.220523  -11.054082    1.163472
C   -7.715717    7.373379    0.678608
H   -7.823587    7.489760   -0.405098
H   -8.409762    6.600352    1.025566
H   -7.962854    8.316096    1.163472
C   10.243392    2.995318    0.678608
H   10.398116    3.030545   -0.405098
H    9.920953    3.982892    1.025566
H   11.183377    2.737986    1.163472
C    0.845287    8.219091    0.466550
H    1.601333    8.631181   -0.209632
H    0.000000    7.850680   -0.124119
H    0.497010    9.005390    1.133675
C    6.695298   -4.841585    0.466550
H    6.674155   -5.702386   -0.209632
H    6.798888   -3.925340   -0.124119
H    7.550392   -4.933118    1.133675
C   -7.540585   -3.377506    0.466550
H   -8.275488   -2.928795   -0.209632
H   -6.798888   -3.925340   -0.124119
H   -8.047401   -4.072272    1.133675
```

15d, free ligand

Stoichiometry: $C_{66}H_{96}N_6O_{12}P_4$

Charge: 0

Multiplicity: 1

Point group: C_3

DLPNO-CCSD(T), Hartree: -5160.9679917

Gibbs in HMPA, Hartree: -5159.6319863

Nuclear coordinates, Å:

```
C   -0.349984    2.759170    0.587877
H   -0.373942    2.805842   -0.499038
H   -1.379475    2.668788    0.935432
C    2.564503   -1.076490    0.587877
H    3.000976   -0.139734    0.935432
H    2.616901   -1.079078   -0.499038
C   -2.214519   -1.682680    0.587877
H   -2.242959   -1.726764   -0.499038
H   -1.621501   -2.529054    0.935432
C    0.479818    1.572637    1.063580
H    1.535457    1.738183    0.827935
H    0.408960    1.451496    2.149956
C    1.122035   -1.201853    1.063580
H    0.737582   -2.198836    0.827935
H    1.052552   -1.079918    2.149956
C   -1.601852   -0.370784    1.063580
H   -2.273039    0.460654    0.827935
H   -1.461512   -0.371578    2.149956
P    0.332297    4.319543    1.335650
P    3.574685   -2.447549    1.335650
P   -3.906983   -1.871993    1.335650
P    0.000000    0.000000    0.202857
C    5.281320   -2.328506    0.638140
C    6.155880   -3.359339    1.008471
C    5.855178   -1.291557   -0.105816
C    7.499660   -3.385631    0.657236
C    7.200304   -1.286487   -0.465869
C    8.045815   -2.331487   -0.081335
H    8.113209   -4.218782    0.956947
H    7.586161   -0.467439   -1.049244
C   -0.624114    5.738011    0.638140
C   -0.168667    7.010818    1.008471
C   -1.809068    5.716512   -0.105816
C   -0.817787    8.187712    0.657236
C   -2.486021    6.878890   -0.465869
C   -2.003781    8.133624   -0.081335
H   -0.403032    9.135636    0.956947
H   -3.388266    6.803528   -1.049244
C   -4.657206   -3.409504    0.638140
C   -5.987213   -3.651479    1.008471
C   -4.046111   -4.424954   -0.105816
C   -6.681873   -4.802080    0.657236
C   -4.714282   -5.592402   -0.465869
C   -6.042034   -5.802137   -0.081335
H   -7.710177   -4.916854    0.956947
H   -4.197895   -6.336089   -1.049244
C    1.927830    4.439693    0.418004
C    2.043174    4.547933   -0.973553
C    3.131503    4.414674    1.134527
C    3.272563    4.626484   -1.620567
C    4.374642    4.489274    0.513207
C    4.458780    4.584867   -0.879545
H    3.305793    4.717376   -2.693324
H    5.270455    4.469634    1.110604
C    2.880972   -3.889396    0.418004
C    2.917038   -4.043407   -0.973553
C    2.257468   -4.919298    1.134527
C    2.370371   -5.147365   -1.620567
C    1.700505   -6.033188    0.513207
C    1.741221   -6.153850   -0.879545
H    2.432471   -5.221588   -2.693324
H    1.235589   -6.799165    1.110604
C   -4.808802   -0.550297    0.418004
C   -4.960212   -0.504526   -0.973553
C   -5.388971    0.504624    1.134527
C   -5.642934    0.520881   -1.620567
C   -6.075146    1.543913    0.513207
C   -6.200001    1.568983   -0.879545
```

H	-5.738263	0.504213	-2.693324
H	-6.506044	2.329531	1.110604
O	3.020603	4.311916	2.477834
O	2.223927	-4.771877	2.477834
O	-5.244530	0.459961	2.477834
O	0.881112	4.571763	-1.663406
O	3.518707	-3.048947	-1.663406
O	-4.399819	-1.522816	-1.663406
O	-2.756111	-4.222787	-0.466457
O	5.035096	-0.275469	-0.466457
O	-2.278985	4.498256	-0.466457
O	-6.564409	-2.665626	1.736835
O	5.590705	-4.352132	1.736835
O	0.973704	7.017758	1.736835
C	-2.062748	-5.233421	-1.149336
H	-2.035593	-6.165757	-0.575284
H	-2.501448	-5.434508	-2.133451
H	-1.044591	-4.870599	-1.277941
C	5.563650	0.830318	-1.149336
H	6.357499	1.320004	-0.575284
H	5.957146	0.550937	-2.133451
H	4.740358	1.530657	-1.277941
C	-3.500901	4.403103	-1.149336
H	-4.321906	4.845753	-0.575284
H	-3.455698	4.883571	-2.133451
H	-3.695767	3.339942	-1.277941
C	-4.518359	-1.552202	-3.060197
H	-5.565267	-1.606162	-3.377521
H	-4.044713	-0.681598	-3.526669
H	-4.002181	-2.454029	-3.384845
C	0.914933	4.689114	-3.060197
H	1.391656	5.622744	-3.377521
H	1.432075	3.843623	-3.526669
H	-0.124161	4.693005	-3.384845
C	3.603425	-3.136913	-3.060197
H	4.126342	-2.238976	-3.384845
H	4.173611	-4.016582	-3.377521
H	2.612638	-3.162025	-3.526669
C	-7.871948	-2.836640	2.209988
H	-8.593870	-2.917106	1.389784
H	-7.959313	-3.719949	2.851914
H	-8.098143	-1.946927	2.795205
C	6.392576	-5.398987	2.209988
H	6.823223	-5.983957	1.389784
H	7.201227	-5.032993	2.851914
H	5.735160	-6.039734	2.795205
C	1.479372	8.235627	2.209988
H	1.770647	8.901063	1.389784
H	0.758086	8.752942	2.851914
H	2.362983	7.986662	2.795205
C	-5.743871	1.515612	3.254250
H	-5.285234	2.472494	2.983032
H	-6.833057	1.604308	3.171559
H	-5.485178	1.277688	4.284527
C	4.184494	4.216532	3.254250
H	4.783860	3.340900	2.983032
H	4.805900	5.115447	3.171559
H	3.849099	4.111459	4.284527
C	1.559377	-5.732144	3.254250
H	0.501374	-5.813394	2.983032
H	2.027157	-6.719755	3.171559
H	1.636078	-5.389147	4.284527
N	-2.687701	9.288649	-0.411668
C	-3.750401	9.220677	-1.378170
C	-2.048401	10.561319	-0.210476
H	-3.414324	8.869336	-2.364787
H	-4.548619	8.554557	-1.039793
H	-4.185088	10.211666	-1.499982
H	-1.797385	10.712993	0.842553
H	-1.125461	10.678042	-0.797260
H	-2.736542	11.355093	-0.497229
N	-6.700356	-6.971941	-0.411668
C	-8.122170	-7.054627	-0.210476
C	-6.110140	-7.858281	-1.378170
H	-8.379032	-6.913077	0.842553
H	-8.684725	-6.313699	-0.797260
H	-8.465529	-8.047461	-0.497229
H	-5.973908	-7.391559	-2.364787
H	-5.134154	-8.216498	-1.039793
H	-6.751018	-8.730225	-1.499982
N	9.388056	-2.316707	-0.411668
C	9.860541	-1.362396	-1.378170
C	10.170571	-3.506693	-0.210476
H	9.388232	-1.477777	-2.364787
H	9.682773	-0.338059	-1.039793
H	10.936106	-1.481440	-1.499982
H	10.176417	-3.799916	0.842553
H	9.810186	-4.364343	-0.797260
H	11.202070	-3.307632	-0.497229
N	1.154579	-7.234292	-1.511937
C	1.428893	-7.461236	-2.907079
C	0.750446	-8.369691	-0.723893
H	1.100452	-6.614647	-3.515091
H	2.495083	-7.632190	-3.113713
H	0.872689	-8.335736	-3.241114
H	1.587816	-8.844436	-0.192935
H	0.000000	-8.086382	0.018387
H	0.296223	-9.112751	-1.377530
N	5.687791	4.617041	-1.511937
C	5.747174	4.968076	-2.907079
C	6.873142	4.834751	-0.723893
H	5.178227	4.260343	-3.515091
H	5.362129	5.976900	-3.113713
H	6.782615	4.923638	-3.241114
H	6.865598	5.797307	-0.192935
H	7.003012	4.043191	0.018387
H	7.743762	4.812913	-1.377530
N	-6.842371	2.617251	-1.511937
C	-7.176067	2.493161	-2.907079
C	-7.623588	3.534940	-0.723893
H	-6.278678	2.354304	-3.515091
H	-7.857212	1.655290	-3.113713
H	-7.655303	3.412097	-3.241114
H	-8.453414	3.047129	-0.192935
H	-7.003012	4.043191	0.018387
H	-8.039986	4.299839	-1.377530

15b·PF_3 complex
Stoichiometry: $C_{24}H_{54}F_3P_5Pt$
Charge: 0
Multiplicity: 1
Point group: C_1
DLPNO-CCSD(T), Hartree: -3068.5139555
Gibbs in gas, Hartree: -3067.8410305
Nuclear coordinates, Å:

C	2.296957	-1.239204	-2.400613
H	1.994784	-2.290929	-2.395808
H	3.339053	-1.218360	-2.731955
C	-0.453690	2.630080	-2.322553
H	-1.030103	3.530444	-2.553106
H	0.597801	2.886478	-2.484022
C	-2.419011	-1.681806	-1.989985
H	-3.179427	-0.895378	-2.021558
H	-2.944793	-2.627074	-2.151789
P	2.169746	-0.704251	-0.589601
P	-0.561526	2.232112	-0.475791
P	-1.732337	-1.599127	-0.228811
Pt	-0.037700	-0.026075	-0.381238
P	-0.224271	-0.113587	-2.614246
C	2.860808	-2.281449	0.177268
H	2.072092	-2.998098	-0.075625
C	0.580166	3.597525	0.139631
H	1.542864	3.256873	-0.255648
C	-3.367863	-1.301518	0.658081
H	-3.615554	-0.294683	0.306061
C	-1.410286	-3.416309	0.131581
H	-2.388111	-3.902109	0.207172
C	-2.262691	2.910462	-0.051899
H	-2.193725	3.999915	-0.132087
C	3.626359	0.477470	-0.457464
H	4.531964	-0.129769	-0.552406
C	-3.321168	2.432224	-1.036695
H	-3.176371	2.843321	-2.036842
H	-3.317549	1.342264	-1.115123
H	-4.315475	2.736308	-0.697610
C	-0.636902	-4.085582	-0.996391
H	-1.218116	-4.152928	-1.917170
H	0.283384	-3.538703	-1.215828
H	-0.357103	-5.103209	-0.709538
C	3.624748	1.497550	-1.588137
H	3.815572	1.041476	-2.560669
H	2.666822	2.020374	-1.641561
H	4.402423	2.247685	-1.419177
C	0.691179	3.617819	1.657708
H	0.786741	2.613498	2.069098
H	1.565357	4.199404	1.963789
H	-0.182404	4.089558	2.112441
C	2.932277	-2.200949	1.695459
H	2.029769	-1.750553	2.110235
H	3.047364	-3.200957	2.123221
H	3.789337	-1.609570	2.023523
C	-3.196501	-1.225937	2.168584
H	-2.316163	-0.641830	2.437952
H	-4.072295	-0.758904	2.627587

H   -3.096357   -2.221285   2.606663
C   0.322059   5.001706   -0.394314
H   -0.625585   5.405404   -0.030301
H   1.107791   5.678107   -0.044069
H   0.313561   5.049656   -1.483664
C   4.173925   -2.806903   -0.390821
H   5.009723   -2.142958   -0.158556
H   4.407841   -3.777268   0.057899
H   4.144181   -2.947331   -1.471776
C   -4.525868   -2.224851   0.298249
H   -4.344815   -3.250058   0.629440
H   -5.436768   -1.886978   0.801972
H   -4.735492   -2.247669   -0.771540
C   -2.656483   2.539689   1.370468
H   -1.971376   2.940756   2.115398
H   -3.658947   2.916104   1.595970
H   -2.657470   1.456032   1.493636
C   -0.667346   -3.578383   1.449820
H   -1.234671   -3.202362   2.300191
H   -0.444887   -4.633812   1.634089
H   0.271791   -3.024216   1.422565
C   3.636642   1.180658   0.891893
H   3.741052   0.489025   1.726243
H   4.465149   1.893790   0.940840
H   2.703105   1.722983   1.046448
C   -1.405862   -1.441554   -3.112079
H   -1.916528   -1.213228   -4.052556
H   -0.800685   -2.335131   -3.282545
C   -0.842601   1.495454   -3.273848
H   -0.479725   1.697180   -4.286295
H   -1.929234   1.398765   -3.334414
C   1.414100   -0.469762   -3.386545
H   1.319208   -1.016197   -4.329832
H   1.854786   0.502417   -3.620050
P   0.293294   -0.185492   3.785009
F   1.618722   0.668621   3.626078
F   -0.724035   1.029243   3.822189
F   0.399259   -0.355173   5.367786

15b·PF_3 complex, transition state
Stoichiometry: $C_{24}H_{54}F_3P_5Pt$
Charge: 0
Multiplicity: 1
Point group: C_1
DLPNO-CCSD(T), Hartree: -3068.4892257
Gibbs in gas, Hartree: -3067.8163567
Nuclear coordinates, Å:

C   -0.855395   2.496517   -2.224349
H   0.104318   3.022006   -2.235618
H   -1.604695   3.208542   -2.581835
C   -1.520932   -2.208013   -2.184686
H   -1.719983   -3.248341   -2.455636
H   -2.451906   -1.658625   -2.352689
C   2.921443   -0.476522   -2.015283
H   2.817357   -1.565683   -2.041435
H   3.949521   -0.259983   -2.317309
P   -1.169286   2.089905   -0.412650
P   -1.215993   -2.073219   -0.330595
P   2.657792   -0.010363   -0.221834
Pt -0.248299   -0.013075   -0.102712
P   0.214829   -0.052620   -2.374352
C   -0.436533   3.665286   0.313868
H   0.572021   3.634521   -0.116161
C   -2.993443   -2.311578   0.234907
H   -3.478947   -1.472941   -0.276801
C   3.603806   -1.421647   0.579224
H   2.964375   -2.276178   0.331645
C   3.813575   1.456477   0.004478
H   4.839837   1.075429   0.035643
C   -0.426052   -3.746650   0.004671
H   -1.212211   -4.482058   -0.192226
C   -3.021679   2.376556   -0.331790
H   -3.173065   3.426604   -0.601742
C   0.726045   -4.031376   -0.949915
H   0.394516   -4.161636   -1.980620
H   1.463137   -3.225296   -0.927336
H   1.235673   -4.951878   -0.651988
C   3.713328   2.455873   -1.140059
H   4.047632   2.041897   -2.092424
H   2.686052   2.811188   -1.262985
H   4.334344   3.330463   -0.926269
C   -3.755177   1.517831   -1.353230
H   -3.526537   1.802440   -2.381173
H   -3.502812   0.462540   -1.225106
H   -4.835281   1.620706   -1.217903
C   -3.188919   -2.098428   1.728357
H   -2.729834   -1.172425   2.067236
H   -4.258011   -2.059570   1.956975
H   -2.763336   -2.916053   2.312934
C   -0.280483   3.632661   1.826210
H   0.276127   2.755509   2.158531
H   0.268055   4.518131   2.160311
H   -1.241632   3.629628   2.341012
C   3.620573   -1.293640   2.098063
H   2.638305   -1.047461   2.504613
H   3.945924   -2.234432   2.550346
H   4.322335   -0.522983   2.423910
C   -3.671390   -3.599840   -0.217736
H   -3.282564   -4.467292   0.320181
H   -4.741627   -3.546414   0.001916
H   -3.567165   -3.788526   -1.287254
C   -1.102366   4.962735   -0.129277
H   -2.085569   5.082266   0.331127
H   -0.496197   5.815951   0.188752
H   -1.224198   5.032395   -1.211460
C   5.002450   -1.704858   0.046797
H   5.687830   -0.879507   0.253992
H   5.414828   -2.590959   0.539244
H   5.015783   -1.892557   -1.027633
C   0.041955   -3.901032   1.443605
H   -0.753459   -3.738320   2.168514
H   0.438866   -4.909146   1.595319
H   0.832707   -3.185836   1.674233
C   3.495521   2.154310   1.323005
H   3.541915   1.485898   2.181875
H   4.197190   2.975492   1.496740
H   2.487137   2.571907   1.297696
C   -3.584319   2.131657   1.059464
H   -3.128417   2.767745   1.817238
H   -4.661027   2.324941   1.062624
H   -3.418537   1.098249   1.365201
C   1.933978   0.143650   -3.009160
H   2.067130   -0.287990   -4.006572
H   2.105062   1.218822   -3.099747
C   -0.425573   -1.630412   -3.081769
H   -0.794163   -1.499280   -4.104099
H   0.428559   -2.308810   -3.134696
C   -0.777801   1.290562   -3.157121
H   -0.373113   1.576785   -4.132493
H   -1.771804   0.872627   -3.331173
P   0.008084   0.197572   2.588038
F   -1.451740   0.781905   2.902508
F   -0.309402   -1.326944   2.970883
F   0.501121   0.520322   4.138536
